# Contribution of proteasome-catalyzed peptide *cis*-splicing to viral targeting by CD8^+^ T cells in HIV-1 infection

**DOI:** 10.1073/pnas.1911622116

**Published:** 2019-11-20

**Authors:** Wayne Paes, German Leonov, Thomas Partridge, Takayuki Chikata, Hayato Murakoshi, Anna Frangou, Simon Brackenridge, Annalisa Nicastri, Andrew G. Smith, Gerald H. Learn, Yingying Li, Robert Parker, Shinichi Oka, Pierre Pellegrino, Ian Williams, Barton F. Haynes, Andrew J. McMichael, George M. Shaw, Beatrice H. Hahn, Masafumi Takiguchi, Nicola Ternette, Persephone Borrow

**Affiliations:** ^a^Nuffield Department of Clinical Medicine, University of Oxford, Oxford OX3 7FZ, United Kingdom;; ^b^York Cross-Disciplinary Centre for Systems Analysis, University of York, York YO10 5DD, United Kingdom;; ^c^Centre for AIDS Research, Kumamoto University, Kumamoto 860-0811, Japan;; ^d^Big Data Institute, University of Oxford, Oxford OX3 7LF, United Kingdom;; ^e^Nuffield Department of Medicine, University of Oxford, Oxford OX3 7BN, United Kingdom;; ^f^Department of Medicine, Perelman School of Medicine, University of Pennsylvania, Philadelphia, PA 19104;; ^g^Department of Microbiology, Perelman School of Medicine, University of Pennsylvania, Philadelphia, PA 19104;; ^h^AIDS Clinical Centre, National Centre for Global Health and Medicine, Tokyo 162-8655, Japan;; ^i^Centre for Sexual Health and HIV Research, University College London, London WC1E 6JB, United Kingdom;; ^j^Department of Medicine, Duke University School of Medicine, Durham, NC 27710;; ^k^Duke Human Vaccine Institute, Duke University School of Medicine, Durham, NC 27710

**Keywords:** peptide splicing, proteasome, immunopeptidome, T cell epitope, human immunodeficiency virus

## Abstract

Peptides generated by proteasome-catalyzed splicing of noncontiguous amino acid sequences have been shown to constitute a source of nontemplated human leukocyte antigen class I (HLA-I) epitopes, but their role in pathogen-specific immunity remains unknown. CD8^+^ T cells are key mediators of HIV type 1 (HIV-1) control, and identification of novel epitopes to enhance targeting of infected cells is a priority for prophylactic and therapeutic strategies. To explore the contribution of proteasome-catalyzed peptide splicing (PCPS) to HIV-1 epitope generation, we developed a broadly applicable mass spectrometry-based discovery workflow that we employed to identify spliced HLA-I–bound peptides on HIV-infected cells. We demonstrate that HIV-1–derived spliced peptides comprise a relatively minor component of the HLA-I–bound viral immunopeptidome. Although spliced HIV-1 peptides may elicit CD8^+^ T cell responses relatively infrequently during infection, CD8^+^ T cells primed by partially overlapping contiguous epitopes in HIV-infected individuals were able to cross-recognize spliced viral peptides, suggesting a potential role for PCPS in restricting HIV-1 escape pathways. Vaccine-mediated priming of responses to spliced HIV-1 epitopes could thus provide a novel means of exploiting epitope targets typically underutilized during natural infection.

## Significance

CD8^+^ T cells target virus-infected and tumor cells by recognition of peptides presented on human leukocyte antigen (HLA)-I molecules. Many of these peptides are generated by proteasome-mediated protein degradation. Proteasomes can also “cut-and-paste” noncontiguous amino acid sequences to generate spliced peptides. However, the contribution of spliced epitopes to T cell-mediated viral control is unknown. Here, we developed a mass spectrometry-based workflow for identification of spliced HLA-I–bound peptides on HIV-infected cells and analyzed the role of responses to the spliced viral peptides detected in HIV targeting in infected individuals. We show that although spliced peptides comprise a minor fraction of the viral targets on HIV-infected cells they enhance the available epitope breadth and may limit viral escape, facilitating HIV control.

Peptides presented by human leukocyte antigen (HLA) class I and II molecules were originally thought to derive solely from contiguous protein sequences. Two seminal studies then reported T cell responses to noncontiguous (spliced/fusion) peptides in renal cell carcinoma and melanoma patients, although identification of these tumor-derived spliced peptides involved labor-intensive in vitro approaches, and validation relied on the serendipitous availability of epitope-specific T cells (1, 2). Thus, only a handful of *cis*-spliced epitopes were described in more than a decade of subsequent research (3–7). In addition to epitopes generated by *cis*-splicing, which involves ligation of noncontiguous peptide fragments within the same polypeptide, recognition of HLA-II–restricted epitopes generated by fusion of peptide fragments from 2 different proteins (*trans*-splicing) by CD4^+^ T cells from patients with type I diabetes has also been reported (8), although whether the peptide fusion event leading to generation of these *trans*-spliced epitopes was catalyzed by the proteasome or other enzymes was not explored.

More recently, the increased sensitivity of mass spectrometry (MS)-based approaches for characterizing the repertoire of HLA-bound peptides (the immunopeptidome) has provided an opportunity for the discovery of spliced peptides on a much larger scale than was previously possible (9–11). Although proteasome-catalyzed peptide splicing (PCPS) had widely been assumed to be a relatively rare event, MS-based profiling of immunopeptidomic datasets has questioned this assumption. However, the proportion of HLA-I–bound peptides generated by PCPS remains controversial (9–11). Furthermore, the extent to which peptides generated by PCPS are targeted by CD8^+^ T cell responses and the contribution of spliced epitope recognition to immune control of infections or tumors remain unclear. The biological significance of PCPS in the in vivo immune response thus represents a key unanswered question.

Virus-specific CD8^+^ T cells are known to play an important role in the control of HIV type 1 (HIV-1) replication, prompting the development of strategies to harness their activity for HIV-1 prophylaxis and therapy (12, 13). During HIV-1 infection, CD8^+^ T cell antiviral efficacy is undermined by viral evasion strategies, including the ability of HIV to evolve mutations in/around HLA-I–restricted epitopes that confer escape from epitope-specific CD8^+^ T cells (14). This highlights the need for codominant targeting of a great breadth of functionally constrained HLA-I epitopes to limit viral escape and the challenges in achieving this in chronically infected individuals where the viral reservoir may contain preexisting escape mutations (14, 15). Recent studies have illustrated the potential utility of vaccine-induced CD8^+^ T cell-mediated targeting of previously unrecognized viral epitopes (16, 17), underscoring the urgent need for a more comprehensive understanding of the repertoire of HIV-1 peptides presented by HLA-I on infected cells to inform the rational design of CD8^+^ T cell-based strategies to combat HIV.

Here, we sought to determine whether peptides generated by proteasomal *cis*-splicing of viral sequences are presented with HLA-I on HIV-infected cells and to evaluate the contribution that this poorly characterized method of epitope generation may be making to CD8^+^ T cell-mediated control of HIV-1 replication in infected individuals. We developed a broadly applicable MS-based discovery workflow to enable identification of *cis*-spliced HLA-I–bound peptides on HIV-infected cells and employed this to analyze datasets generated by in-depth MS-based profiling of the HIV-1 immunopeptidome in 15 HIV-infected monoallelic cell lines and one multiallelic line. Using this approach, we show that in addition to canonical contiguous HIV-1–derived peptides, posttranslationally spliced viral peptides are presented on infected cells. Notably, we estimate that only a small percentage of the peptides within the MS-detectable HIV-1 immunopeptidome are generated by PCPS.

Screening of infected individuals for responses to 3 experimentally validated spliced HIV-1 peptides suggested that spliced peptides may prime CD8^+^ T cell responses relatively infrequently during infection, putatively because epitopes generated by splicing may be less abundant than contiguous epitopes. However, CD8^+^ T cell responses primed by contiguous epitopes in infected individuals were able to cross-recognize partially overlapping spliced peptides, indicating a potential role for PCPS as a host-mediated mechanism of diversifying the antigenic repertoire of HLA-I–bound peptides and restricting routes of viral escape during natural infection. Together, these results suggest that vaccine-induced targeting of spliced HIV-1 peptides could provide a novel strategy for enhancing CD8^+^ T cell response breadth beyond that achieved during natural infection to facilitate HIV prophylaxis and therapy.

## Results

### Development of a Generalizable de Novo Sequencing Workflow for Spliced Peptide Identification.

**Fig. 1.**
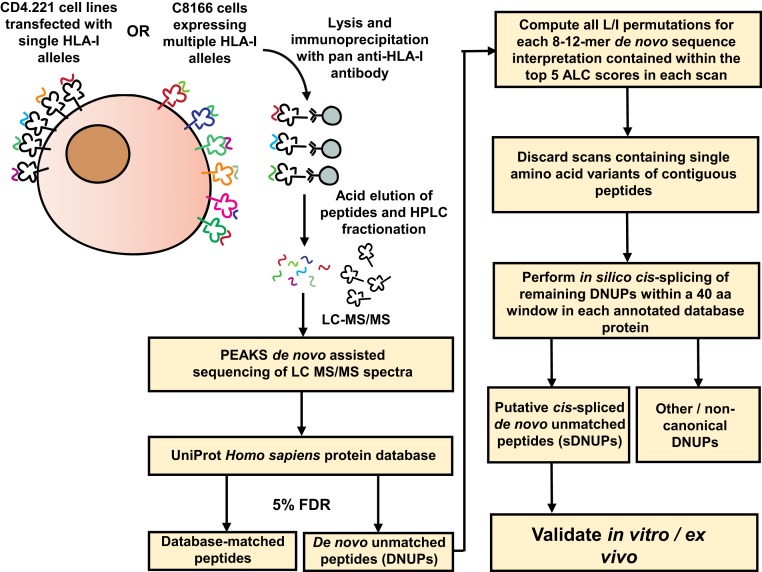
Workflow for identification of HLA-I–bound spliced peptides by de novo sequencing. HLA class Ia (HLA-Ia)-deficient CD4.221 cells individually transfected with a panel of HLA-I alleles, or the C8166 cell line (expressing 5 distinct HLA-Ia alleles), were lysed and HLA-I–peptide complexes isolated using the pan anti–HLA-I antibody W6/32. Peptides were eluted and then separated by high-performance liquid chromatography fractionation prior to LC-MS/MS sequencing. Following spectral assignment to contiguous peptide sequences in the UniProt database, sequence interpretations (including all L/I permutations) for each DNUP residing within the top 5 ALC scores in each scan were considered. Scans containing single amino acid variants of contiguous sequences within the canonical proteome were removed and the remainder fragmented in silico into 2 splice partners and matched to the annotated UniProt proteome to create a list of sDNUPs. Post hoc database matching with artificial spliced proteins was not implemented (see also *Materials and Methods* and *SI Appendix*, Fig. S1).

To determine whether spliced HIV-1 epitopes were presented by HLA-I on infected cells, constituting previously undescribed targets for CD8^+^ T cell recognition, we developed a robust method to interrogate the MS-detectable immunopeptidome for spliced peptides. For method development, sample datasets were generated by immunoprecipitation of HLA-I–peptide complexes from 3 non-HIV-infected single HLA-I allele-transfected CD4.221 cell lines (A*11:01, B*57:03, and C*03:03) and the C8166 cell line expressing A*01:01, B*08:01, B*44:02, C*05:01, and C*07:01 (Fig. 1). Following liquid chromatography tandem MS (LC-MS/MS), de novo assisted sequencing of HLA-I bound peptides was performed using PEAKS software (18). Sequenced spectra were first matched to the annotated UniProt *Homo sapiens* database (19), allowing for all common posttranslational modifications (PTMs) with a false discovery rate (FDR) of 5%. After identification of database-matched contiguous peptides, the remaining pool of unassigned (non-database-matched) spectra were termed “de novo unmatched peptides” (DNUPs).

Each DNUP sequence interpretation was assigned an average local confidence (ALC) score by PEAKS. This is the mean value of the local confidence score at each amino acid position and is expressed as a percentage. Higher ALC scores reflect greater confidence in the overall de novo sequence for a given spectrum. For identification of spliced peptides, an ALC score of 50% was set as the default minimum for reporting of DNUP sequences. Detailed methodological descriptions outlining the computation of leucine/isoleucine (L/I) permutations (which was required because LC-MS/MS is unable to distinguish between L and I due to their identical mass) and down-selection of DNUP candidates (as depicted in Fig. 1) are presented in *Materials and Methods*.

We only sought to identify peptides originating from splicing of 2 distinct fragments within the same protein (i.e., *cis*-spliced). Because an experimentally validated precedent exists for *cis*-splicing of 2 constituent noncontiguous peptide fragments to occur over a distance of 40 amino acids (aa) within a protein (20), this was the maximum window within which a *cis*-splicing event was allowed to occur in silico for each sDNUP. *Trans*-spliced peptides (that may originate from 2 distinct proteins) were not defined as part of the analysis. Although a HLA-I–bound peptide containing a single amino acid ligated to a longer peptide fragment has previously been described (4), 1-mer splice partners were not considered in our analysis due to the potential occurrence of single amino acid variants or the possibility of mistranslation and RNA editing (21, 22). The final list of DNUPs fulfilling the above splicing criteria were termed “putative *cis*-spliced” DNUPs (sDNUPs). The remaining DNUPs not assigned as *cis*-spliced peptides (and which did not match the human proteome as nonspliced peptides) were termed other/noncanonical peptides.

Importantly, in contrast to the few MS-based approaches for spliced peptide discovery described to date (9–11, 23), we did not implement a post hoc database-matching step on artificially created spliced protein databases following in silico splicing of DNUPs. Our decision to omit this step was based on analysis of the effects of post hoc spectral matching against databases created by appending artificial spliced proteins to the annotated UniProt proteome (*SI Appendix*, Fig. S1 *A* and *B*), which showed that this limited the breadth of epitope discovery.

### ALC Threshold Determines the Breadth of sDNUP Discovery.

**Fig. 2.**
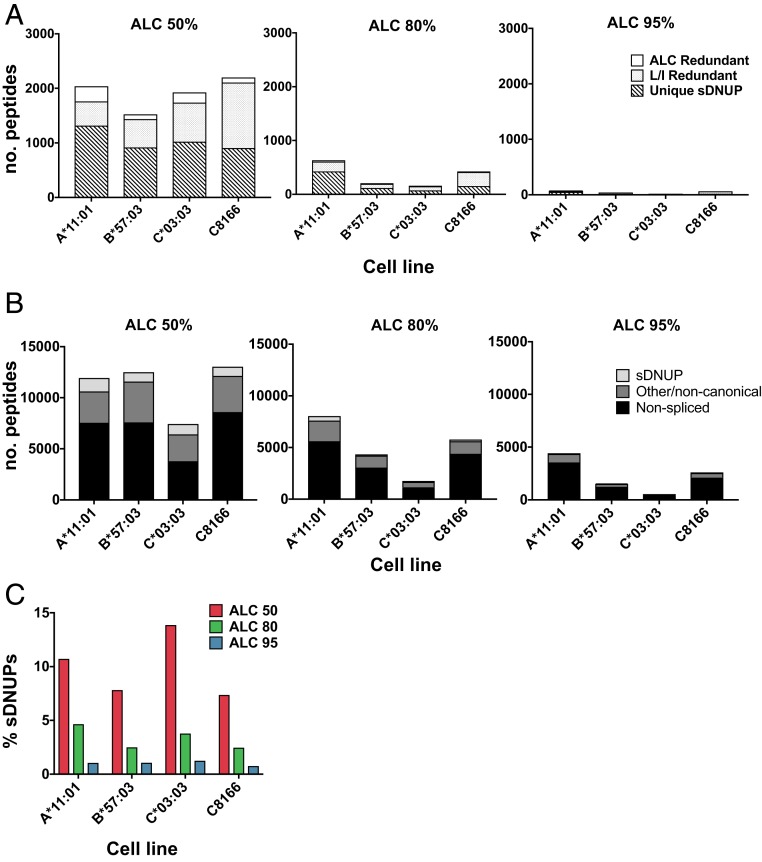
Proportion and redundancy of identified sDNUPs. (*A*) The number of unique, L/I redundant, and ALC redundant sDNUPs in datasets from the indicated cell lines are shown at each ALC cutoff. (*B*) The number of nonspliced, sDNUP, and other/noncanonical (non-*cis*-spliced) peptides contributing to the MS-detectable immunopeptidome of the indicated cell lines at each ALC cutoff. (*C*) The proportion of assigned sDNUPs (expressed as a percentage of all unique MS spectra) at each ALC threshold.

To inform the selection of an ALC threshold for spliced peptide discovery in our workflow, we first considered the ALC score distribution of de novo sequences assigned to database-matched nonspliced peptides (*SI Appendix*, Fig. S1*C*). Selecting an ALC score threshold of 50% was found to incorporate greater than 80% of all canonical contiguous peptides in each dataset, so this was chosen as a minimum threshold for sDNUP discovery. Use of an ALC score threshold of 50% enhanced the breadth of sDNUP identification by incorporating a greater proportion of de novo sequences into the workflow (Fig. 2*A*).

The possibility of interpreting more than one of the top-scoring DNUPs for a given MS/MS spectrum as a spliced peptide candidate led to “redundancy” within sDNUP candidates, as depicted in Fig. 2*A*. We identified 2 main sources of spectral redundancy in the workflow: L/I permutation redundancy and ALC redundancy. L/I redundancy resulted from the likelihood of splicing one or more L/I permutation variants of a single sDNUP sequence interpretation within de novo sequences containing a leucine residue (e.g., EIP-KVLFK and ELPK-VIFK), while ALC redundancy occurred when 2 or more distinct de novo sequence interpretations with an identical ALC score arose from a single peptide spectrum and could be explained by 2 distinct splicing events (e.g., SA-SKDAIKK or TG-SKDAIKK).

Although both types of redundancy introduce additional sDNUPs that require more involved validation, inclusion of these sequences avoids potential omission of candidate spliced peptides. However, to investigate the proportion and characteristics of sDNUPs in each sample, we only considered sDNUPs that resulted from a single *cis*-splicing event (unique sDNUPs). The full lists of unique sDNUPs and nonspliced peptides for each dataset are provided in Datasets S1 and S2.

The number of unique sDNUPs varied depending on the ALC threshold. For example, in the A*11:01 dataset, 1,316 unique sDNUPs were identified at an ALC cutoff of 50%, in comparison to only 52 unique identities when an ALC threshold of 95% was implemented (Fig. 2*B*). Hence, at an ALC cutoff of 50%, 1,316/12,271 (10.72%) of MS-detectable peptides were assigned as sDNUPs, compared to only 52/4,966 (1.05%) at an ALC cutoff of 95% (Fig. 2*C*). This percentage also differed depending on the allele. Thus, an estimate of the percentage (by diversity) of MS-detectable sDNUPs cannot be reliably obtained from HLA-I–derived “self” immunopeptidomic datasets, although the overall proportion is likely significantly lower than the value of ∼30% as previously reported (9, 10). Indeed, reanalysis of the data by Liepe et al. (9) in a recent study reported a frequency of ∼2 to 5% at an ALC threshold of 80%, which is concordant with our findings (11).

To determine the proportion of peptides designated as sDNUPs that might originate from alternative transcripts as contiguous peptides, RNA-sequencing (RNA-seq) analysis of the parental CD4.221 cell line was implemented. Following 3-frame translation of the resulting RNA transcripts from 3 biological replicates, all 3 groups of peptides (nonspliced, other/noncanonical, and sDNUPs) originating from the A*11:01, B*57:03, and C*03:03 datasets were matched to the resulting sequences (*SI Appendix*, Fig. S1*D*). Overall, 95.6% of nonspliced peptides matched to RNA-seq transcripts, with 7.9% of other/noncanonical and 1.6% of sDNUPs also matching to sequenced transcripts. Hence, the vast majority of sDNUPs identified in our workflow likely arise from PCPS events.

### HLA-I–Binding Motifs and Length Distributions of sDNUPs Mirror Those of Nonspliced Peptides.

**Fig. 3.**
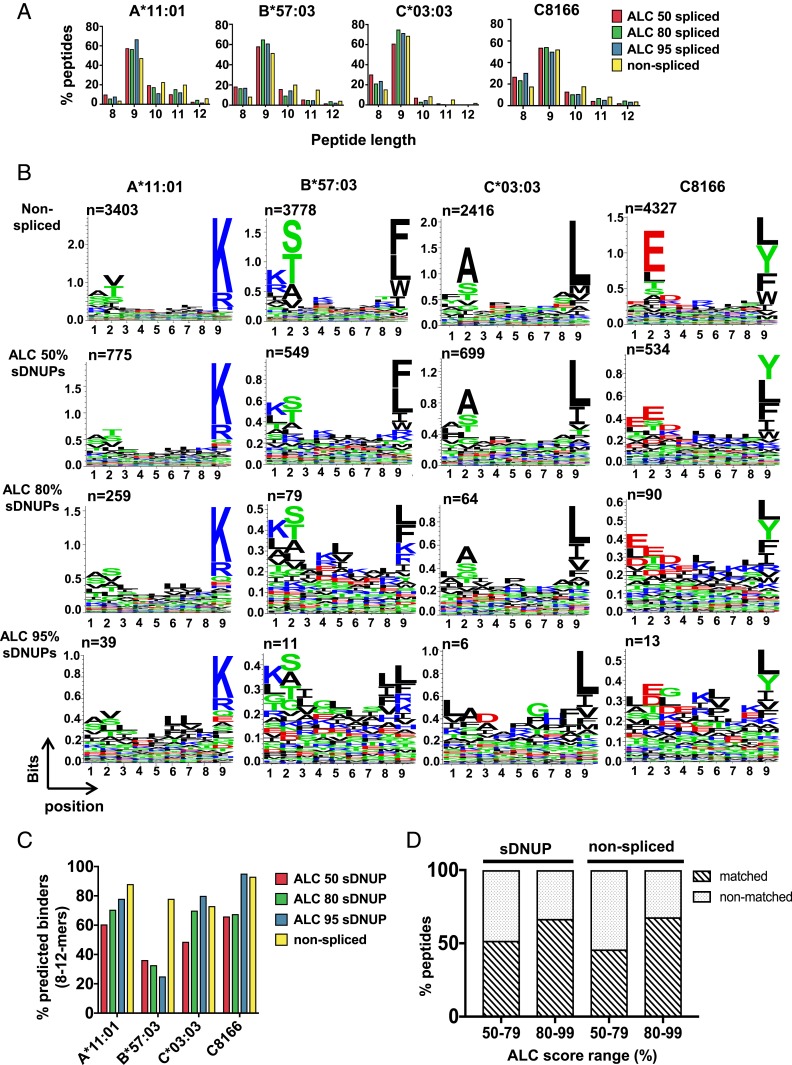
Characteristics of sDNUPs and nonspliced peptides. (*A*) Peptide length distributions of nonspliced peptides and sDNUPs at the indicated ALC thresholds, expressed as a percentage of all unique 8- to 12-mers. (*B*) Sequence motifs of nonspliced and sDNUP 9-mers identified at each ALC threshold as generated by Seq2logo. The number of peptides (n) incorporated at each ALC cutoff is indicated below each panel. See also *SI Appendix*, Fig. S2. (*C*) Percentage of nonspliced peptides and sDNUPs at the indicated ALC cutoffs predicted to bind to the relevant HLA-I allele by NetMHCpan4.0. (*D*) Percentage of the nonspliced peptides and sDNUPs tested of those with ALC scores in the indicated ranges that exhibited spectral matches to corresponding synthetic peptide standards. See also Dataset S3.

Because peptide binding is ultimately determined by the preferences of specific HLA-I–binding clefts, the properties of sDNUPs and nonspliced peptides eluted from a particular allele would be expected to be very similar. Following identification of sDNUPs, we therefore compared their HLA-I–binding characteristics to those of nonspliced peptides. The peptide length distributions of the sDNUPs identified at each ALC threshold closely mirrored those of nonspliced peptides for each allele, with A*11:01- and B*57:03-bound peptides showing similar proportions of longer 10/11-mers, and C*03:03-derived peptides including higher proportions of shorter 8/9-mer peptides (Fig. 3*A*). Expression of B*08:01 (in the C8166 line), which is known to have a preference for binding shorter 8-mer peptides (24), also resulted in correspondingly elevated proportions of shorter 8-mer sDNUPs and nonspliced peptides.

Following generation of sequence logos for 9-mers eluted from each dataset, we observed that HLA-I–binding motifs of sDNUPs showed enrichment of the same amino acids at canonical anchor residue positions as allele-matched nonspliced peptides, which is a key prerequisite in enabling high-affinity peptide binding to the restricting HLA-I allele (Fig. 3*B*). A strong correlation between amino acid frequencies in nonspliced peptides and sDNUPs was also observed (*SI Appendix*, Fig. S2).

### Higher ALC Score Thresholds Enhance the Accuracy of Peptide Spectral Assignments.

Next, we assessed the proportion of peptides in each dataset predicted to bind to their respective allele(s). Binding affinities for sDNUPs and nonspliced peptides identified in our workflow were predicted using the NetMHCpan4.0 algorithm (25). In general, we found that for sDNUPs an increasing proportion of predicted binders was observed as the ALC cutoff increased, although this was not consistently observed across all datasets (possibly due in part to the low number of sDNUPs identified at higher ALC cutoffs) (Fig. 3*C*). With greater ALC scores, a higher proportion of spectra would be more accurately sequenced and thus more likely to contain the correct HLA-I–binding motifs.

To more comprehensively determine the effect of ALC threshold on the reliability of sDNUP identification, spectra of synthetic peptide standards were compared to those of corresponding sDNUPs or nonspliced peptides in each dataset across 2 ALC score ranges (Dataset S3). In total, across all datasets, 133 sDNUPs and 51 nonspliced peptides were synthesized for spectral matching. For sDNUPs, we observed that 44/75 sDNUP spectra (58.6%) within the ALC 80 to 99% threshold and 30/58 spectra (51.7%) within the ALC 50 to 79% threshold achieved high-confidence spectral matches to their corresponding HLA-I–eluted peptides (Fig. 3*D*). This trend was also consistent for nonspliced peptides within each ALC score range and indicates that de novo spectra with higher ALC scores will generally result in a greater proportion of correctly sequenced annotations. However, as shown, implementing more stringent ALC cutoffs would result in omission of a significant proportion of correctly sequenced spectra. Hence, for identification of spliced epitopes derived from a large proteome (such as that in a tumor), use of a high ALC threshold could be implemented to maximize the proportion of correctly identified peptides within the large pool detected, but for identification of spliced peptides derived from the relatively small HIV-1 proteome we chose to use an ALC threshold of 50% to maximize detection of candidate spliced peptides and subsequently conduct stringent experimental validation of the HIV-derived sDNUPs identified.

### HIV-1–Derived Spliced Peptides Are Presented on Virus-Infected Cells.

We then employed the discovery workflow we had developed to identify spliced viral peptides from 15 HIV-1–infected CD4.221 single HLA-I transfectants (comprising a range of HLA-A, -B, and -C alleles), as well as the C8166 cell line (expressing multiple HLA-I alleles). In-depth profiling of the HIV-1 immunopeptidome across a broad range of alleles was performed to enable a reasonably accurate assessment of the proportion of MS-detectable spliced viral epitopes.

HIV-1 peptides that we previously reported as nonspecifically coeluted during HLA-I immunoprecipitations were discarded from the analysis (26). Dataset S4 shows the full list of HIV-1–derived HLA-I–restricted nonspliced peptides and sDNUPs discovered in our workflow (the former including a number of contiguous HIV-1 peptides that have not previously been described). From a total of 198 unique HIV-1–derived 8- to 12-mer peptides identified across all datasets, 5 were initially assigned as sDNUPs: FSD-QLIHLY (A*01:01, Vif), VAK-IRKVL (B*08:01, Pol), ALIK-PLPSV (B*13:02, Vif), AEWDRLH-AA (B*50:01, Gag), and FGEETT-KEL (C*03:03, Gag). These HIV-1–derived sDNUPs were confirmed not to originate from any sequence in the canonical human proteome following in silico splicing or to match any contiguous cryptic sequence in any of the 6 viral open reading frames. Furthermore, they were not detected in 3-frame translation of RNA-seq transcripts derived from HIV-infected CD4.221 cells. They were also not detected in MS datasets from the corresponding uninfected cell lines.

**Fig. 4.**
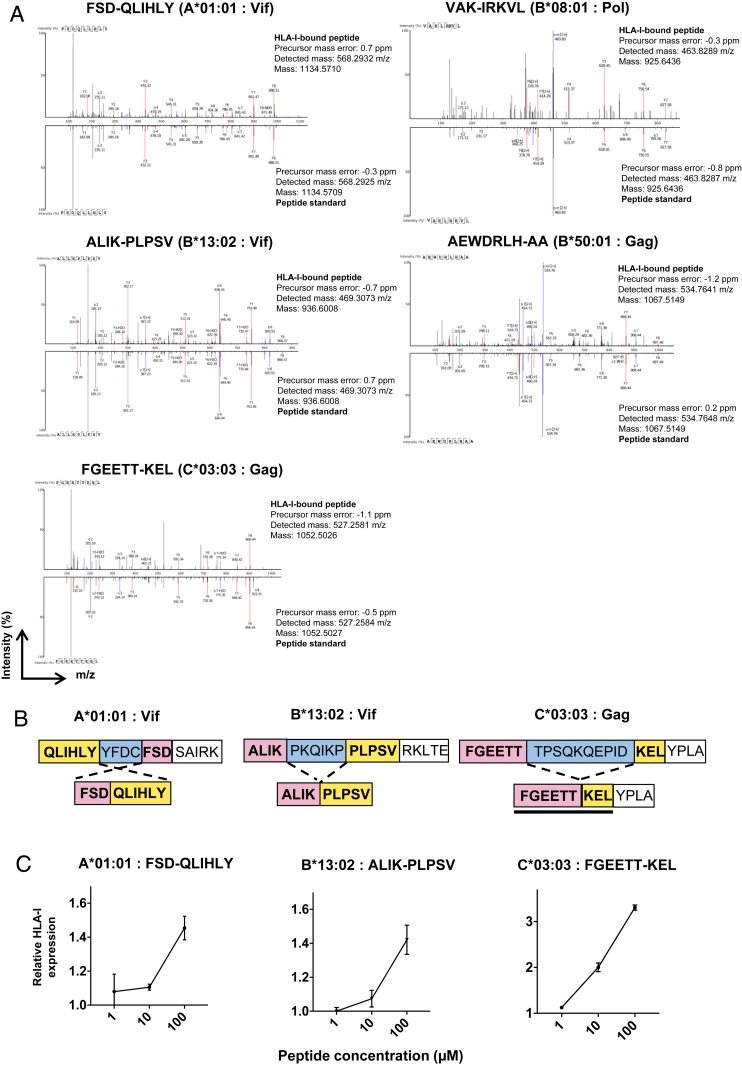
HIV-1 spliced peptides presented on HLA-I are generated by (immuno)proteasomal splicing. (*A*) Spectral matching of HLA-I–bound HIV-1–derived sDNUPs with synthetic peptide standards. (*B*) Schematic illustrating generation of the spliced peptide products from HIV-1 precursor peptides following (immuno)proteasomal digestion. See also *SI Appendix*, Fig. S3. (*C*) RMA-S HLA-I stabilization assays confirming binding of HIV-1–derived spliced peptides to relevant HLA-I alleles. The relative fluorescence intensities of peptide-pulsed to non-peptide-pulsed cells are depicted at log-fold titrations of peptide. Data are expressed as the mean relative fluorescence values ± the SD from triplicate experiments.

Spectral matching of HIV-1–derived sDNUPs with synthetic peptide standards confirmed the identity of the eluted peptides (Fig. 4*A*). To determine whether these sDNUPs could be generated from viral protein sequences by PCPS, in vitro proteasomal digests were performed on corresponding synthetic HIV-1 precursor peptides. MS sequencing of (immuno)proteasomal digests confirmed the generation of 3/5 HIV-1 derived sDNUPs by PCPS (Fig. 4*B*). Spectra of the (immuno)proteasomal digest-derived peptides also matched those of synthetic peptide standards with high confidence (*SI Appendix*, Fig. S3). We further confirmed binding of these *cis*-spliced HIV-1 peptides to their restricting HLA-I molecules by performing HLA-I stabilization assays in RMA-S cell lines transduced with the relevant alleles (Fig. 4*C*). However, we did not observe PCPS of [VAK-IRKVL] or [AEWDRLH-AA] in vitro. These putative sDNUPs may have originated from noncanonical/alternative transcripts that were not detected by RNA sequencing rather than being created by PCPS, although we cannot exclude that these peptides may be generated by PCPS in HIV-1–infected cells.

Overall, we identified a total of 193 unique contiguous HLA-I–bound HIV-1 epitopes and an additional 3 to 5 unique *cis*-spliced viral epitopes (∼1.5 to 2.5% of all of the HIV-1 epitopes detected) in the datasets examined. Hence, even considering the fact that fusion peptides with a 1-mer splice partner were excluded from our analysis and that a low level of peptide splicing may occur over distances longer than those considered in our study, our results indicate that spliced epitopes comprise only a minor proportion (by diversity) of the MS-detectable HIV-1 immunopeptidome. Importantly, there was no significant difference in the ratio of sDNUPs to nonspliced peptides between HIV-1–infected and corresponding uninfected datasets, indicating that infection did not globally impair PCPS.

### HIV-1–Derived Spliced Peptides Are Cross-Recognized by Patient T Cells Primed by Partly Overlapping Contiguous Epitopes.

We next sought to investigate whether CD8^+^ T cell responses were induced to the 3 experimentally validated spliced HIV-1 epitopes during natural infection in HIV-1^+^ individuals expressing the corresponding HLA-I alleles. Since identification of spliced peptides was performed using laboratory-adapted subtype B viral strains (NL4-3 and IIIB), to inform selection of patients for response screening we examined the conservation of the sequences containing the spliced epitopes in the HIV-1 subtype B and subtype C consensus sequences available in LANL (http://www.hiv.lanl.gov/, 2017 version of the database) (*SI Appendix*, Fig. S4). A glutamate residue (E) was observed more predominantly than aspartate (D) at P3 of the A*01:01-restricted spliced epitope (FSE-QLIHLY) in subtype B sequences, and the epitope was not conserved in the subtype C consensus sequence. ALIK-PLPSV was highly conserved in subtype C but less conserved in subtype B, and FGEETT-KEL was conserved in subtype B viruses only.

Correspondingly, 13 A*01:01^+^ individuals recruited into acute or chronic infection cohorts at clinical sites in the United States and United Kingdom where subtype B infections predominate, 16 B*13:02^+^ individuals recruited at sites in Africa where subtype C infections are most common, and 35 C*03:03^+^ individuals recruited at sites in the United Kingdom and Japan where subtype B infections are most prevalent were selected from available cohorts for screening. All subjects were screened for responses at time points prior to initiation of antiretroviral therapy. Most subjects were chronically infected at screening, with some subjects screened during acute infection (Dataset S5).

The selected HIV-infected individuals were tested for T cell responses to the original NL4-3/IIIB as well as the subtype-consensus versions of the HIV-1 spliced epitopes. Importantly, we observed that the A*01:01-restricted reverse spliced (FSD-QLIHLY) and B*13:02-restricted forward spliced (ALIK-PLPSV) epitopes originating from the HIV-1 Vif protein overlapped with contiguous epitopes (LADQLIHLY and KQIKPPLPSV) (*SI Appendix*, Fig. S4) that were coeluted from HLA-I in the same experiments (Dataset S4). We thus also screened for responses to these overlapping contiguous epitopes.

**Fig. 5.**
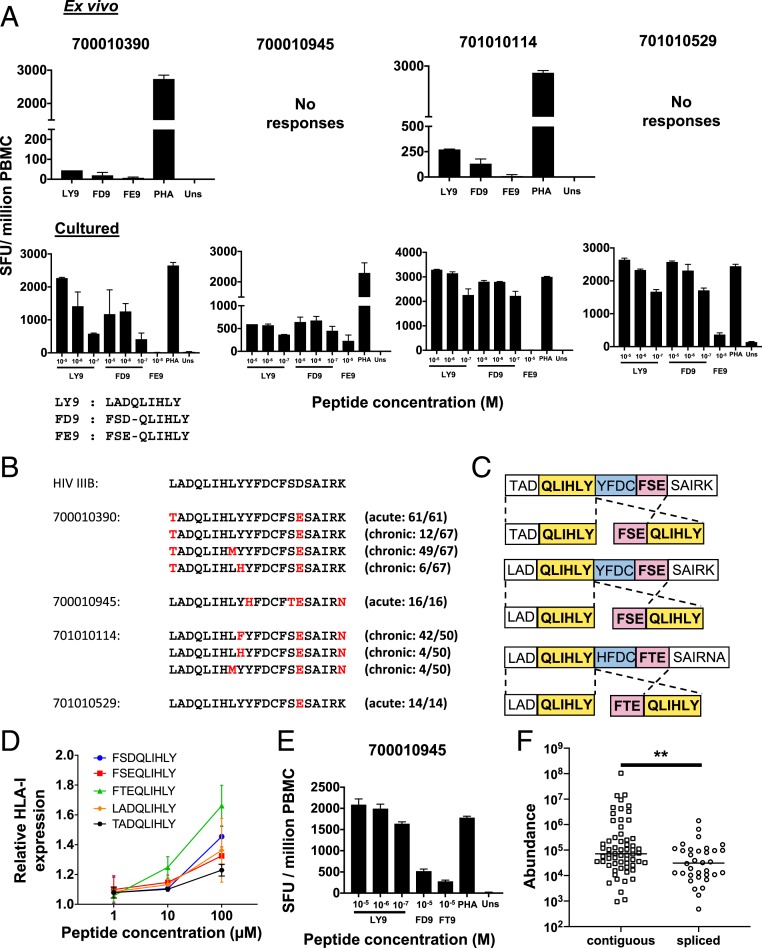
HIV-1 spliced peptides are cross-recognized by CD8^+^ T cell responses primed to overlapping contiguous epitopes in infected individuals. (*A*) Responses detected in IFN-γ ELISpot assays ex vivo (*Top*) or following T cell expansion by in vitro culture with the contiguous epitope LADQLIHLY (*Bottom*) to the HIV-1 IIIB contiguous LADQLIHLY (LY9) and spliced FSD-QLIHLY (FD9) epitopes and the predominant subtype B consensus sequence (see also *SI Appendix*, Fig. S4) version of the spliced epitope FSE-QLIHLY (FE9) in 4 A*01:01^+^ HIV-infected individuals (700010390, 701010114, 701010529, and 700010945). Cells stimulated with PHA and medium only (unstim) served as positive and negative controls, respectively. Data are expressed as SFU per million PBMC, and the mean ± the SD of duplicate wells are shown. (*B*) Sequence alignments of HIV-IIIB and predominant autologous viral sequences present in the 4 HIV-1^+^ A*01:01^+^ individuals at the indicated sampling time points during acute and/or chronic infection. Amino acid residues that differ from the IIIB sequence are shown in red. See also *SI Appendix*, Fig. S4. (*C*) Schematic illustrating HIV-1 precursor peptides subjected to (immune)proteasomal digestion and the spliced and optimal length contiguous epitope products detected by MS. (*D*) RMA-S HLA-I stabilization assays comparing the relative binding affinities of contiguous (LADQLIHLY) and spliced viral epitopes (FSD-QLIHLY, FSE-QLIHLY, and FTE-QLIHLY) to HLA-A*01:01. Data are expressed as the mean relative fluorescence values ± SD from triplicate experiments. (*E*) Responses detected in an IFN-γ ELISpot assay following T cell expansion by in vitro culture with the contiguous epitope LADQLIHLY to the former peptide and to the IIIB FSD-QLIHLY (FD9) and autologous virus FTE-QLIHLY (FT9) versions of the spliced epitope peptide. Data are expressed as in *A*. (*F*) Abundance values of unique contiguous and spliced peptides originating from immunoproteasomal digests of HIV polypeptides described in Figs. 4*B* and C. Group medians are indicated; **P<0.01, two-tailed unpaired Mann-Whitney *t* test.

Screening was performed using both ex vivo and cultured interferon-γ (IFN-γ) enzyme-linked immunosorbent spot (ELISpot) assays, to facilitate detection of even low-magnitude responses. No responses were detected to the B*13:02-restricted Vif-derived spliced or contiguous epitopes (ALIK-PLPSV and KQIKPPLPSV) or to the C*03:03-restricted spliced Gag epitope (FGEETT-KEL) in any subject at the timepoints tested. However, T cell responses were observed to both the A*01:01-restricted contiguous epitope LADQLIHLY (LY9) and the HIV-1 IIIB spliced epitope FSD-QLIHLY (FD9) in ex vivo assays in 2/13 A*01:01^+^ individuals and in a further 2 subjects following expansion of epitope-specific T cells in cultured ELISpot assays (Fig. 5*A*). No recognition of the subtype B consensus sequence version of the A*01:01-restricted spliced epitope FSE-QLIHLY (FE9) was detected in ex vivo assays, although T cells expanded following in vitro culture with the contiguous epitope LADQLIHLY were found to cross-recognize FSE-QLIHLY in 2 of the 4 subjects tested (Fig. 5*A*).

Single genome amplification-based HIV-1 sequence analysis of plasma samples from the 4 A*01:01^+^ subjects in whom responses were detected revealed differences between the HIV-1 IIIB sequence and their autologous viral sequences in the region of Vif encoding the contiguous and spliced A*01:01-restricted epitopes (Fig. 5*B*). Three of the subjects had been sampled in acute infection, enabling inference of the transmitted-founder virus sequence, and one of these (700010390) was also resampled in chronic infection, allowing analysis of viral sequence evolution over time. Subject 701010114 was only sampled during chronic infection, by which time a mixture of viruses with variant epitope sequences similar to those observed during chronic infection in subject 700010390 were present.

To determine whether the spliced viral epitopes could be generated from each patient’s deduced autologous transmitted-founder virus sequence by PCPS, the corresponding precursor peptides were synthesized and digested in vitro. In each case, the splicing event was observed (Fig. 5*C*). Binding of the autologous transmitted-founder spliced epitope sequences to HLA-A*01:01 was also confirmed in RMA-S HLA-I stabilization assays (Fig. 5*D*). These results indicate the potential for in vivo presentation of HLA-A*01:01-restricted spliced peptides in all 4 of these infected individuals. In subject 700010945, where the autologous viral sequence of the spliced epitope peptide was FTE-QLIHLY rather than the subtype B consensus version FSE-QLIHLY, cross-recognition was also confirmed by cultured ELISpot assay (Fig. 5*E*).

Overall, there was no evidence that the 3 spliced HIV-1 epitopes studied here had elicited priming of CD8^+^ T cell responses in individuals of appropriate HLA types infected with viruses that were expected (on the basis of subtype consensus sequences) or confirmed (by autologous virus sequencing) to contain sequences from which the spliced peptide could be generated. Furthermore, in the case of the FSD-QLIHLY epitope, 4 patients were identified in whom responses had been primed to an overlapping contiguous peptide (LADQLIHLY) with a similar affinity of binding to HLA-A*01:01 that were capable of cross-recognizing the spliced peptide, confirming the existence of spliced-epitope reactive T cells in the T cell repertoire. Together, these results suggest that HIV-1 epitopes generated by PCPS do not prime CD8^+^ T cell responses in infected individuals as efficiently as contiguous viral epitopes. Comparison of the abundance of all spliced and contiguous peptides generated during in vitro immunoproteasomal digests of HIV-1 precursor peptides containing the 3 validated spliced epitopes revealed that the former were significantly less abundant (*P* = 0.0082, Mann–Whitney test) (Fig. 5*F*), which may provide an explanation for their inferior in vivo T cell priming capacity.

### Escape Mutations Abrogate T Cell Recognition of Both Contiguous and Spliced HIV-1 Epitopes.

**Fig. 6.**
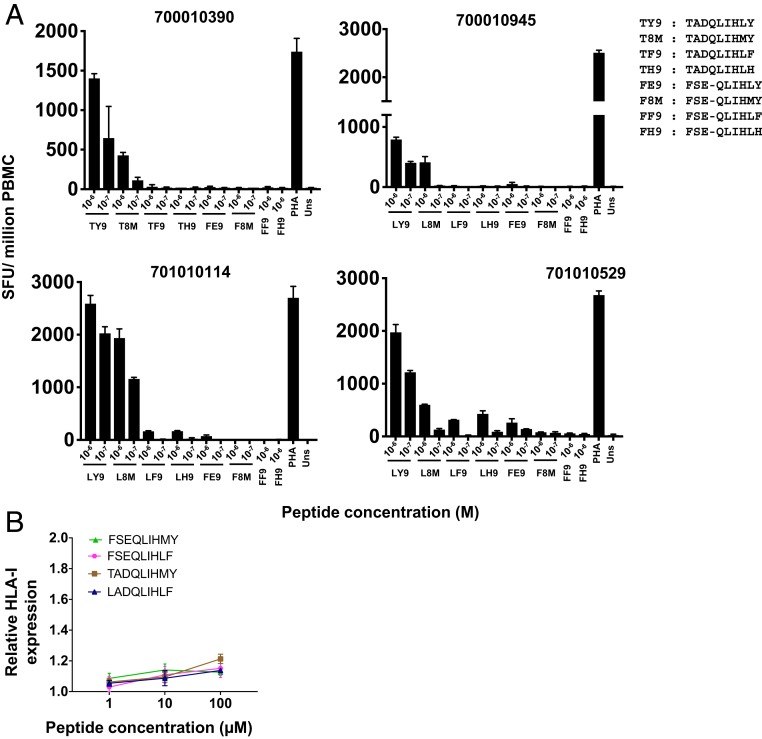
Mutations abrogate CD8^+^ T cell recognition of both contiguous and spliced epitopes. (*A*) Responses detected to mutant contiguous LADQLIHMY (L8M), LADQLIHLF (LF9), and LADQLIHLH (LH9) and mutant spliced FSE-QLIHMY (F8M), FSE-QLIHLH (FH9), and FSE-QLIHLF (FF9) peptides in cultured IFN-γ ELISpot assays following T cell expansion by in vitro culture with autologous contiguous viral epitopes LADQLIHLY (LY9) or TADQLIHLY (TY9). Data are expressed as SFU per million PBMC, and the mean ± SD of duplicate wells are shown. (*B*) RMA-S HLA-I stabilization assays comparing the relative binding affinities of dominant mutant versions of the nonspliced (LADQLIHLF and TADQLIHMY) and spliced (FSE-QLIHLF and FSE-QLIHMY) viral epitopes to HLA-A*01:01. Data are expressed as the mean relative fluorescence values ± SD from triplicate experiments.

Despite the fact that spliced HIV-1 epitopes may have a lower propensity to elicit CD8^+^ T cell priming than contiguously encoded epitopes in infected individuals, spliced peptide presentation on HIV-infected cells may nonetheless have a role to play in enhancing CD8^+^ T cell-driven reduction of HIV replication by necessitating escape via acquisition of mutation(s) that abrogate recognition of both the spliced and contiguous peptides. In subject 700010390, variant viruses encoding a leucine-to-methionine change at P8 or a tyrosine-to-histidine change at P9 (comprising the C-terminal anchor of both the contiguous and spliced epitope peptide) were observed in the in vivo quasispecies during chronic infection (Fig. 5*B*). Consistent with this, in subject 701010114 a similar repertoire of Vif sequences in the viral quasispecies was observed during chronic infection, with an additional tyrosine-to-phenylalanine change at the P9 anchor residue of the epitopes. T cell recognition of the relevant mutant versions of the contiguous and spliced viral epitopes was assessed by cultured IFN-γ ELISpot assay following expansion with the appropriate autologous contiguous epitopes (TY9 or LY9) in each patient (Fig. 6*A*). Although no longitudinal sequence data were available for 700010945 and 701010529, T cell reactivity against the same variant epitope sequences observed in 700010390 or 701010114 was also tested in these patients.

While all of the 3 mutations resulted in diminished T cell recognition of the contiguous epitope, mutations where canonical C-terminal P9 anchor residues were altered (LF9 and LH9) showed the greatest reduction in T cell reactivity, likely indicating viral escape from epitope-specific CD8^+^ T cells. Furthermore, mutant versions of the corresponding spliced epitopes did not elicit detectable responses, suggesting that escape mutations resulted in abrogation of T cell recognition of both contiguous and spliced viral peptides. Contiguous and spliced peptides containing the dominant mutations observed in the majority of the viral quasispecies in each patient (LF9, FF9, T8M, and F8M) were subsequently tested in HLA-A*01:01 stabilization assays. These mutations significantly impaired binding of both the contiguous and spliced epitopes to HLA-A*0101 (Fig. 6*B*), suggesting that the observed abrogation of CD8^+^ T cell recognition may be due to a reduction in affinity and diminished binding to HLA-A*01:01.

## Discussion

The importance of PCPS as a means of epitope generation in the in vivo immune response is currently unknown. Here, we have identified HLA-I–bound HIV-1 peptides generated by proteasomal splicing in virus-infected CD4^+^ T cells and provide insight into the contribution these peptides might make to control of viral replication in HIV-infected individuals. We show that spliced peptides comprise only a minor proportion of the HIV-1–derived immunopeptidome. We also found no evidence that the spliced HIV-1 peptides identified here elicited CD8^+^ T cell priming during natural infection, but observed cross-recognition of spliced peptides by CD8^+^ T cell responses primed to overlapping contiguous epitopes. In addition, we document in vivo selection of HIV-1 mutations conferring escape from CD8^+^ T cell recognition of both the contiguous and spliced viral epitopes, suggesting a role for PCPS in limiting viral escape pathways and enhancing CD8^+^ T cell-driven reduction of HIV-1 replication.

Identification of spliced viral peptides presented with HLA-I on HIV-infected cells was achieved by development of a broadly applicable de novo sequencing-based discovery workflow, which was stringently evaluated prior to implementation for HIV-1 spliced peptide definition. Importantly, we sought to address the advantages as well as inherent caveats of using a database-independent de novo sequencing approach for spliced peptide identification. In previous studies, creation of artificial spliced proteins composed of ligated strings of very similar peptide sequences that are not representative of annotated proteomes has been used to identify spliced peptides (9, 10). This may differentially impact the shortlisting of proteins for peptide spectral matching by different search algorithms and further confound peptide spectral assignments. Indeed, we found that a large proportion of sDNUP sequences that were omitted following a post hoc database validation step with artificial spliced proteins were in fact correctly sequenced, matching with high confidence to corresponding synthetic peptide standards, thus indicating that omission of this step will result in increased breadth of discovery.

Although high-energy collisional dissociation fragmentation was implemented in our LC-MS/MS approach, alternative hybrid fragmentation methods such as electron transfer/higher-energy collision dissociation that enable high-confidence spectral assignment of HLA-bound peptides (27) may mitigate potential ambiguity in de novo peptide sequences. Incomplete fragmentation at peptide N termini may sometimes reduce the accuracy of de novo peptide spectral assignments (28), leading to incorrect assignment of terminal amino acid residues. Although HLA-I–binding motifs for sDNUPs largely mirrored those of nonspliced peptides, this may account for the somewhat lower proportion of sDNUPs predicted to bind to a given HLA-I allele than nonspliced peptides in our study. In addition, 3-frame translation of CD4.221-derived RNA transcripts revealed that a small proportion (∼1.6%) of sDNUPs may also be matched to contiguous sequences originating from alternative transcripts. However, in addition to such previously described noncanonical translation products (29–31), *trans*-spliced peptides as well as low translational fidelity of mRNA transcripts or RNA editing may result in nongenomically encoded peptide sequences that may also contribute to the immunopeptidome and could be misassigned as sDNUPs (10, 21, 22).

The contribution of spliced peptides to the HLA-I–bound peptide repertoire has generated controversy. After interrogating presentation of both contiguous and spliced HIV-1 epitopes by diverse HLA-I alleles, we estimate that the frequency of MS-detectable *cis*-spliced peptides in the HIV-1 immunopeptidome is very low. We cannot be certain that every spliced HIV-1 peptide in our datasets was identified. As discussed above, de novo peptide spectral assignment is not perfect, and peptides present at lower abundance, which may include a higher proportion of spliced than contiguous peptides, may be more prone to misidentification or fall below the threshold of detection. Furthermore, we excluded fusion peptides with a 1-mer splice partner from our analysis and did not seek to identify peptides generated by splicing over distances longer than those over which the majority of PCPS events are thought to occur (20). Nonetheless, our results compellingly demonstrate that the frequency of spliced peptides in the MS-detectable HIV-1 immunopeptidome is much lower than values initially reported for the frequency of spliced peptides in the self-protein–derived immunopeptidomes of uninfected cell lines (9, 10). Our estimate is more concordant with a recent analysis reporting a contribution of ∼2 to 6% of *cis*-spliced peptides to the MS-detectable cellular immunopeptidome (11) (although the proportion of HLA-I–bound spliced peptides may have been slightly overestimated in the latter study, as other noncanonical sources were not considered).

Our use of a much smaller viral proteome provides a more tractable estimate of the likely proportion of MS-detectable spliced peptides presented with HLA-I. Following transcription of HIV-1 mRNAs from integrated proviral DNA in the nucleus, HIV-1 mRNAs are exported to the cytoplasm and proteins are produced by the cellular translation machinery. Hence, the cellular sites/pathways of HIV-1 protein generation mirror those of host cell proteins. The cellular translational apparatus is modulated by HIV-1 proteins to improve the efficiency of viral translation (32), but we did not observe a significant difference in the ratio of sDNUPs to nonspliced peptides in HIV-1–infected and uninfected cells, suggesting that HIV does not globally impair PCPS. Although we cannot preclude that HIV-1 may have evolved to minimize expansion of the viral immunopeptidome by peptide splicing as a means of immune evasion, this would seem unlikely in the absence of strong CD8^+^ T cell-mediated selective pressure against spliced epitopes. Analysis of HIV-1 epitopes is thus likely to provide a fairly representative estimate of the overall frequency of *cis*-spliced epitopes in the cellular HLA-I–bound immunopeptidome.

Although T cell responses can be primed by spliced epitopes in vivo (1, 2), the contribution made by spliced peptides to the CD8^+^ T cell response elicited following infection or tumor development remains unclear. Here, we found no evidence that the 3 spliced viral epitopes we defined had elicited CD8^+^ T cell priming in HIV-infected individuals expressing the corresponding restricting HLA-I alleles. However, detection of responses in infected individuals may have been confounded by effects of autologous viral sequence variability within the spliced epitope that altered HLA-I-binding/T cell recognition, or by epitope-proximal sequence variation which may have influenced spliced epitope generation by the proteasome. Moreover, we screened for responses to only 3 spliced viral peptides so cannot conclude that T cell responses may not be primed by other spliced epitopes in HIV-infected individuals.

However, our results suggest that spliced HIV-1 epitopes may elicit T cell priming less efficiently than contiguous epitopes during natural infection. A prior study in which the efficiency with which a single spliced epitope was generated in vitro and *in cellulo* was compared to that of contiguous epitope generation suggested that proteasomes generate spliced epitopes as efficiently as contiguous epitopes (6). In contrast, our analysis of 34 spliced and 62 contiguous peptides generated following in vitro immunoproteasomal digests showed a trend for spliced peptides to be less abundant than contiguous viral peptides, putatively indicating a lower efficiency of PCPS reactions. However, future analysis of the *in cellulo* abundance of large numbers of validated spliced and contiguous peptides derived from a variety of source proteins will be required to determine the relative efficiencies of contiguous and spliced peptide generation by both constitutive and immunoproteasomes. Although we have not addressed levels of spliced versus contiguous peptide generation during antigen processing for cross-presentation (which may make an important contribution to CD8^+^ T cell priming during viral infections) (33, 34), lower peptide abundance may reduce the efficiency with which responses are elicited to spliced epitopes in vivo.

Notably, we demonstrated that CD8^+^ T cells primed by overlapping contiguous epitopes in HIV-1–infected individuals were able to cross-recognize spliced HIV-1 epitopes. This is very likely to be due to the amino acids shared by the peptides constituting the principal (or potentially all of the) T cell receptor contact residues in the epitope, as previous studies have shown that peptide sequences as short as 5 aa presented on HLA-I can act as minimal determinants for TCR recognition (35). Cross-recognition of spliced epitopes by contiguous epitope-specific T cells has also been demonstrated in a mouse model of *Listeria monocytogenes* infection (36), where it may enhance T cell recognition of infected cells. In contrast to *L. monocytogenes*, HIV is a highly variable pathogen that employs sequence variation in/around epitopes as a means of evading host immune responses (14). Our findings thus indicate a further role for host-mediated PCPS as a means of diversification of the HLA-I–bound immunopeptidome, which may restrict pathways for the evolution of escape mutations, thereby enhancing the viral fitness costs associated with CD8^+^ T cell evasion and enabling greater overall T cell-mediated reduction in ongoing viral replication.

In summary, although *cis*-spliced epitopes comprise a relatively small proportion of the viral immunopeptidome, and may not elicit CD8^+^ T cell priming as effectively as contiguous epitopes, these peptides may nonetheless contribute to in vivo control of HIV-1 replication in infected individuals. Moreover, the fact that spliced viral peptides are presented in quantities detectable by MS on HIV-1–infected cells provides an opportunity to design vaccines encoding contiguous versions of these epitopes that may elicit responses of greater breadth than those induced during natural infection. Large-scale identification of spliced peptides using our discovery workflow will offer the possibility of enhancing the breadth of vaccine-induced CD8^+^ T cell responses not only in HIV-1 infection but also in other infectious diseases, cancers, and autoimmune disorders.

## Materials and Methods

### Human Subjects.

Peripheral blood mononuclear cells (PBMCs) cryopreserved from HIV-infected individuals recruited as part of studies on HIV-infected individuals in the United Kingdom (MM cohort: 8 patients), Japan (NCGM cohort: 29 patients), or at sites in the United States or Africa (CHAVI 001 cohort: 27 patients), were used. Ethical approval for these studies was obtained from The National Health Service Camden and Islington Community local Research Ethics Committee (MM cohort), Kumamoto University and National Centre for Global Health and Medicine Ethics Committee (NCGM Cohort), and Duke Medicine Institutional Review Board and the ethics boards of the local sites (CHAVI 001 cohort). All patients gave written informed consent. PBMC samples were selected based on donor HLA-I types, without accounting for age, sex, or demographics. HLA typing was performed as previously described (37–39).

### Cell Lines.

All cell lines were mycoplasma-negative and cultured at 37 °C in the presence of 5% CO_2_. The 721.221 HLA-class I deficient cell line transfected with CD4 (CD4.221) and selected HLA class I alleles (40) and the C8166 cell line expressing multiple HLA alleles (A*01:01 homozygous, B*08:01, B*44:02, C*05:01, and C*07:01) (41) were grown in RPMI 1640 medium (Thermo Fisher) containing 10% fetal bovine serum (FBS), 2 mM l-glutamine, 100 U/mL penicillin, and 100 μg/mL streptomycin (R10). For CD4.221 cell lines, R10 medium was supplemented with 0.15 mg/mL hygromycin B (Thermo Fisher) to maintain HLA-I expression. Our panel of 15 single HLA-I transfectants comprised A*02:01, A*11:01/02, B*08:01, B*13:02, B*35:01, B*44:02, B*50:01, B*57:01, B*57:03, C*03:03/04, C*12:02, and C*14:02/03. HEK 293FT cells (Thermo Fisher) were grown in Dulbecco’s modified Eagle’s medium (Thermo Fisher) containing 10% FBS, 2 mM l-glutamine, 100 U/mL penicillin, and 100 μg/mL streptomycin (D10).

### HIV-1 Infection.

The laboratory-adapted HIV-1 IIIB (subtype B, CXCR4-tropic) strain and the NL4-3 infectious molecular clone (also subtype B, CXCR4-tropic) were obtained from the Program EVA Centre for AIDS Reagents, National Institute for Biological Standards and Control. HIV-1 IIIB viral stocks were prepared by growth in C8166 cells as previously described (26). Preparation of NL4-3 stocks and in vitro infections were carried out as detailed in *SI Appendix*, *Methods*.

### Immunoprecipitation and Purification of HLA-I–Associated Peptides.

W6/32 coated resin was prepared as described in *SI Appendix*, *Materials*. Roughly 150 × 10^6^ cells were harvested, washed once in phosphate-buffered saline, and lysed in 5 mL of cell lysis buffer (1% IGEPAL 630, 300 mM NaCl, 100 mM Tris, pH 8.0, plus protease inhibitors) for 45 min at 4 °C. HLA-I was then immunoprecipitated and bound peptides dissociated and purified as detailed in *SI Appendix*, *Methods*.

### Synthetic Peptides.

Peptides were synthesized using Fmoc solid-phase chemistry by Genscript. For spectral matching, synthetic spliced peptides were resuspended to a final concentration of 5 μM.

### In Vitro (Immuno)proteasomal Digests and Spectral Validation.

For (immuno)proteasomal digests, 5 μg of synthetic polypeptides were digested with 500 ng of 20S immunoproteasome or proteasome (Enzo Life Sciences) in 20 mM Hepes (pH 7.8), 5 mM magnesium chloride, and 2 mM dithiothreitol for 2 h or 20 h at 37 °C. Following incubation, reactions were terminated by addition of 5 μL of acetic acid, and digest material was bound to a C18 ZipTip column (Merck), eluted in 30% acetonitrile (CH_3_CN), dried down, and resuspended in 20 μL LC-MS/MS loading buffer (1% acetonitrile and 0.1% trifluoroacetic acid in water); 0.2 μL were analyzed by LC-MS/MS. Spectral matching was performed on 133 sDNUPs and 51 nonspliced peptides identified in our workflow by manual inspection of observed HLA-I–bound spectra and comparison with those of corresponding synthetic peptide spectra (shown in Dataset S3).

### LC-MS/MS Analysis.

LC-MS/MS analysis was performed as described by Partridge et al. (26). A detailed methodological description is provided in *SI Appendix*, *Methods*. LC-MS/MS datasets are available in the PRoteomics IDEntifications (PRIDE) Archive (42), project accession number PXD015489.

### Discovery Workflow for Identification of Spliced Peptides.

The analysis of all LC-MS/MS datasets (.raw files) was performed using PEAKS v8.0 (Bioinformatic Solutions) software. LC-MS/MS scans were searched without enzyme specification using a mass tolerance precursor setting of 5 ppm for peptides and 0.03 Da for fragment ions. PEAKS de novo assisted sequencing was implemented for the assignment of peptide-spectral matches. De novo sequencing was set to output 100 candidates per spectrum for each dataset without variable or fixed modifications. An ALC value was assigned to each peptide-spectrum match based on spectral quality and fragmentation pattern and reflects the mean confidence of the assigned sequence after summation of the confidence scores of each amino acid within the sequence.

De novo assisted peptide sequencing was followed by spectral matching against the annotated *H. sapiens* UniProt database appended with 6 reading frame translation of the HIV-1 IIIB (for A*11:01 and C*12:02 CD4.221 single HLA-I transfectants and C8166 infections) or NL4-3 (for all other single HLA-I CD4.221 transfectants) genomes. An FDR of 5% was set using a parallel decoy database search. Following the database matching of spectra, the PEAKS posttranslational modification (PTM) search results were exported to allow first for matching of spectra to all common PTMs. MS/MS spectra assigned as posttranslationally modified were removed from the analysis.

Subsequent computational steps in our discovery workflow for spliced peptide identification were implemented using Python version 3.7.1. Unmatched peptide spectra of ALC 50% and above were considered. These were termed DNUPs. From a maximum of 100 de novo sequence interpretations for each spectrum, only interpretations contained within the top 5 ALC scores in each scan were incorporated into the discovery workflow for spliced peptide identification.

Because the masses of leucine (L) and isoleucine (I) are identical, LC-MS/MS cannot differentiate between L and I residues within peptides. For this reason, PEAKS de novo sequencing reports all L or I residues as Ls. Therefore, for DNUPs containing ‘n’ leucine residues, all permutations (2^n^) of L/I variants were computed. If a match to a contiguous protein sequence within the UniProt proteome resulted following L/I permutations, the whole LC-MS/MS spectrum/scan was removed from the analysis. Subsequently, DNUPs differing by one amino acid residue from contiguous protein sequences contained within the annotated proteome were removed to account for possible single amino acid variants or mutations contained within the cell lines that may be misassigned as spliced peptides.

In silico splicing of the resulting DNUP sequence interpretations in each scan was implemented by splitting each DNUP into 2 fragments from (*N* − 2) aa to 2 aa, where *n* = 7 < N < 13, and is equivalent to DNUP length. The (*N* − 2)^th^ fragment was scanned for a contiguous match across the proteome and, if found, its corresponding splice partner fragment was scanned for a contiguous match within a 40-aa window either side of the initial match. A DNUP that fulfilled these criteria was termed an sDNUP, and a final list of sDNUPs were compiled for each allele. Here, redundant sDNUPs originating from the same scan as either L/I variants (L/I redundant) or spectral variants (ALC redundant) were discarded from the downstream analyses. Only sDNUPs originating from a single splicing event (unique sDNUPs) were subsequently considered for investigating the properties of HLA-I–bound spliced peptides. The remaining (non-*cis*-spliced) DNUPs that did not match contiguous peptides within the annotated proteome were termed “other/noncanonical” peptides. Code used for data analysis will be supplied on request.

Shannon sequence logos for nonspliced and sDNUP 9-mers were produced using the online tool Seq2logo v2.0 (43); Venn diagrams were produced by using the online tool BioVenn (44). Binding predictions of sDNUP and nonspliced peptides were calculated using NetMHCpan4.0, where threshold percent ranks for strong and weak binders were set at 0.5% and 2%, respectively (25). Heat maps were generated using gnuplot version 5.2.4.

For examining the effects of post hoc database matching with artificial spliced proteins (as in *SI Appendix*, Fig. S1), sDNUPs were appended to the *H. sapiens* UniProt database as concatenated protein entries of ∼500 aa in length (corresponding to the mean length of an annotated UniProt *H. sapiens* protein). The initial raw spectral data were then researched in PEAKS or MaxQuant with the resulting new “spliced” databases.

### RNA-Seq Analysis of CD4.221 Cells.

Three biological replicates (containing 8 × 10^6^ cells) of the CD4.221 cell line either infected with NL4-3 or uninfected were lysed, and RNA was extracted using the RNeasy Mini Kit (Qiagen) according to the manufacturer’s protocol. Complementary DNA was prepared using the Smart-seq2 protocol, then barcoded libraries were prepared and sequenced using an Illumina NextsEq. 500 and the resulting RNA-seq data were analyzed as described in *SI Appendix*, *Methods*.

### RMA-S Binding Assay.

The binding of peptides to HLA-I molecules was measured as previously described (45); an outline of the method used is provided in *SI Appendix*, *Methods*. Peptide binding is expressed as the relative HLA expression index, calculated as MFI of RMA-S cells prepulsed with peptide/MFI of RMA-S cells without peptide pulsing.

### Analysis of Peptide-Specific T Cell Responses in HIV-1–Infected Individuals.

Ex vivo or cultured ELISpot assays were carried out on PBMCs from HIV-positive donors. For ex vivo ELISpots, cryopreserved PBMCs were thawed and rested in R10 overnight then transferred to ELISpot plates (Merck Millipore) at 2 × 10^5^ cells per well. Peptides were added to bring the total volume/well to 200 μL; wells containing phytohemagglutinin (PHA) at 20 μg/mL concentration (Merck) or medium only were included as positive and negative controls. Plates were then incubated for 20 h at 37 °C, 5% CO_2_. Coating, development (MabTech), and reading of ELISpot plates (AID Reader) have been described previously (38). For cultured ELISpots, 1.5 × 10^6^ cells were stimulated with peptide at a final concentration of 10 μg/mL in 24-well plates (Merck Millipore) with addition of 25 ng/μL interleukin (IL)-7 (R&D Systems) on day 0 in RPMI-1640 medium containing 10% human serum type AB, 2 mM l-glutamine, 100 U/mL penicillin, 100 μg/mL streptomycin, and 1 mM sodium pyruvate (Rab-10); 1.8 × 10^3^ units/mL IL-2 (R&D Systems) were added on days 3 and 7, and ELISpot assays were performed on day 10. A positive response was confirmed when the number of spots exceeded 3 times background (media only wells) and was greater than 25 spot-forming units (SFU) per million PBMCs. Zero values were not accepted in any replicate of antigen-stimulated wells.

### Sequencing of Plasma-Derived HIV-1.

Single genome amplification and sequencing of plasma viral RNA was used to generate HIV-1 sequences devoid of PCR artifacts, and 2,194-base pair (nucleotides 3683 to 5877) or 1,682-base pair (nucleotides 4900 to 6581) regions from the HIV-1 HXB2 reference genome were amplified by nested RT-PCR as described (46); details of the primers used are provided in *SI Appendix*, *Materials*. Sequencing of the PCR amplicons was performed using the Illumina MiSeq platform as previously described (47). An outline of the method used is included in *SI Appendix*, *Methods*. Viral sequence data are available in GenBank.

### Statistical Analysis.

Unless otherwise stated, 2-tailed unpaired *t* tests were carried out to assess the statistical significance of differences between groups using GraphPad Prism v7.0. Differences were considered statistically significant at a *P* value of <0.05.

## Supplementary Material

Supplementary File

Supplementary File

Supplementary File
