# Supplementary material for: Contribution of proteasome-catalyzed peptide cis-splicing to viral targeting by CD8+ T cells in HIV-1 infection
Source: Proc Natl Acad Sci U S A. 2019 Nov 20;116(49):24748–59. doi: 10.1073/pnas.1911622116 (PMC6900506; doi:10.1073/pnas.1911622116)
Supplement: Supplementary File [file pnas.1911622116.sapp.pdf]

PNAS
www.pnas.org

Supporting Information for:

Contribution of proteasome-catalyzed peptide *cis*-splicing to viral targeting by CD8^{+} T cells in HIV-1 infection

Wayne Paes, German Leonov, Thomas Partridge, Takayuki Chikata, Hayato Murakoshi, Anna Frangou, Simon Brackenridge, Annalisa Nicastri, Andrew G. Smith, Gerald H. Learn, Yingying Li, Robert Parker, Shinichi Oka, Pierre Pellegrino, Ian Williams, Barton F. Haynes, Andrew J. McMichael, George M. Shaw, Beatrice H. Hahn, Masafumi Takiguchi, Nicola Ternette, Persephone Borrow

Wayne Paes, Nicola Ternette, Persephone Borrow

Email: wayne.paes@ndm.ox.ac.uk, nicola.ternette@ndm.ox.ac.uk, persephone.borrow@ndm.ox.ac.uk

This PDF file includes:

Supporting text (Materials and Methods)
Supporting figures S1 to S4

Other supporting materials for this manuscript include the following:

Supporting datasets S1-S5

www.pnas.org/cgi/doi/10.1073/pnas.1911622116

Supporting Text

Materials

W6/32-coated resin

W6/32 antibody was purified from the W6/32 hybridoma and cross-linked to Protein A Sepharose beads (Amintra) as previously described (1). Briefly, 1 mg of W6/32 antibody was incubated with 2 mL of 50% (v/v) bead slurry, and the final volume brought to 10 mL with PBS. The mixture was incubated at 4°C with gentle rotation. Beads were collected under gravity flow filtration and washed with 10 mL PBS, followed by equilibration with 20 mL 0.2 M triethanolamine (Sigma-Aldrich), pH 8.2. Cross-linking was performed by incubation with 40 mM dimethyl pimelimidate dihydrochloride (Sigma-Aldrich) in 0.2 M Tris pH 8.3 for 1 h at room temperature, and the reaction was terminated by addition of ice cold 0.2 M Tris pH 8.0. Non-cross-linked antibody was removed by addition of citrate buffer pH 3.0 and W6/32-coated bead resin was equilibrated by addition of 50 mL PBS to neutral pH.

Primers for cDNA synthesis from plasma viral RNA and nested PCR amplification

Plasma viral RNA extraction, cDNA synthesis (using primer R1: 5'-TTCTTCCTGCCATAGGAAATGCCTAAGC-3'), and single-genome amplification (SGA) were performed as previously described (2). The primers used for nested PCR amplifications of the 2194 bp region were F1: 5'-ACAGCTGGACTGTCAATGATATACA-3' // R1: 5'-TTCTTCCTGCCATAGGAAATGCCTAAGC-3' for the first round and F2: 5'-CCACAGAAAGCATAGTAATATGGGG-3' // R2: 5'-AAGSAGTTTTAGGYTGRCTTCCTGGATG-3' for second round. Primers used for nested PCR amplifications of the 1682 bp region were F3: 5'-CAAATTAYAAAAATTCAAAATTTTCGGGTTTATTACAG-3' // R3: 5'-ATTRCARTAGAAAAATTCYCCTCYACAATT-3' for the first round and F4: 5'-

GGGTTTATTACAGRGACAGCAGAG-3' // R4: 5'-ACATGGYTTTAGGCTTTSRTCCCATA-3' for second round.

Methods

HIV-1 stock preparation and *in vitro* infections

NL4-3 viral stocks were prepared by transfection of pNL4-3 into HEK 293FT cells. 12 μg of NL4-3 plasmid DNA and 25μl of Lipofectamine 2000 (ThermoFisher) were mixed in 2 mL of OptiMEM (ThermoFisher) by gentle inversion and incubated at room temperature for 30 mins. The mixture was added dropwise to HEK293FT cells (~90% confluent) in a T75 flask (Corning), and topped up with 8 mL of D10. Virus-containing supernatant was harvested 72 hours post transfection, aliquoted, and frozen at −80°C.

C8166 cells were infected in a low volume of R10 with HIV-1 IIIB at a multiplicity of infection (MOI) of 0.01, or 50 μl of NL4-3 IMC stock (RT value = 4×10^2 ng / mL) were added to 8×10^6 CD4.221 cells for 1.5 h at 37°C, after which 20 mL of R10 was added and flasks cultured overnight. At day 1 post infection, a further 20 mL R10 was added and cells were split (1:2) on day 2. Infected flasks were harvested for immunoprecipitation of HLA-peptide complexes on day 3 post-infection. The proportion of cells infected with HIV-1 was determined on day 3 post-infection by intracellular p24 staining, as previously described (3). Levels of infection varied from 20-40% live $p24^+$ cells.

Purification of HLA-I-peptide complexes and peptide elution

Cell lysates were cleared by two centrifugation steps, 2000 × *g* for 10 min followed by 20,000 × *g* for 30 min at 4°C. HLA-peptide complexes were captured from cleared lysates on W6/32-coated Protein A-Sepharose beads overnight at 4°C in a volume of 10 mL. W6/32-bound HLA-peptide complexes were sequentially washed with 20 mL of wash buffer 1

(0.005% IGEPAL, 50 mM Tris pH 8.0, 150 mM NaCl, 5 mM EDTA), wash buffer 2 (50 mM Tris pH 8.0, 150 mM NaCl), wash buffer 3 (50 mM Tris pH 8.0, 400 mM NaCl) and finally wash buffer 4 (50 mM Tris pH 8.0) under gravity flow in Econo-Column glass chromatography columns (Bio-Rad). 5 mL of 10% acetic acid was added to elute peptide-HLA complexes from the beads, and samples were dried down prior to resuspension in 120 μL loading buffer (0.1% TFA, 1% acetonitrile in ultragrade HPLC water). Samples were loaded onto a 4.6 × 50 mm ProSwift RP-1S column (Thermo Fisher Scientific) and eluted using a 500 μL/min flow rate over 10 min from 2 to 34% buffer B (0.1% TFA in acetonitrile) in buffer A (0.1% TFA in water) using an Ultimate 3000 HPLC system (Thermo Scientific). Detection was performed using a variable wavelength detector at 280 nm. Alternate odd and even fractions that did not contain β2-microglobulin were combined and dried prior to resuspension in 20 μL LC-MS/MS loading buffer (0.1% TFA in water).

LC-MS/MS Analysis

Each sample was resuspended in 20 μL loading buffer and 9 ul were injected onto a 3 μm particle size 0.075 mm x 150 mm PepMap C18 trap column and further loaded onto a 2 μm particle size, 75 μm x 50 cm analytical column on an Ultimate 3000 nUPLC system (Thermo Scientific). For HLA-ligand analysis and spectral matching, a linear gradient of 3-25% buffer B (0.1% formic acid, 5% DMSO in acetonitrile) in buffer A (0.1% formic acid, 5% DMSO in water) at a flowrate of 250 μl/min was applied over 1 h to elute peptides. For *in vitro* proteasomal digest samples, a 30 min gradient from 8-50% buffer B in buffer A was applied. Peptides were introduced using an Easy-Spray source at 2000V at 40ºC to a Fusion Lumos mass spectrometer (Thermo Scientific). The ion transfer tube temperature was set to 305ºC. Full MS spectra were recorded from 300-1500 m/z in the Orbitrap at 120,000 resolution with an AGC target of 400,000. Precursor selection was performed using TopSpeed mode at a

cycle time of 2 s. Peptide ions were isolated using an isolation width of 1.2 amu and trapped at a maximal injection time of 120 ms with an AGC target of 300,000. Higher-energy collisional dissociation (HCD) fragmentation was induced at an energy setting of 28 for peptides with a charge state of 2-4, while singly charged peptides were fragmented at an energy setting of 32 at lower priority (for HLA-ligand analysis only). Fragments were analysed in the Orbitrap at 30,000 resolution.

Generation of cDNA libraries using the Smart-seq2 protocol

Samples were stored at -80°C prior to sequencing. Retro-transcription and 16 PCR amplification steps were performed following the Smart-seq2 protocol (4). The thermal conditions for RT and PCR reactions were set according to the Smart-seq2 protocol. After PCR amplification, cDNA libraries were purified using Ampure XP magnetic beads (ThermoFisher) at a ratio of 0.8 to 1 with cDNA. After purification, the libraries were resuspended in 17.5 µl of buffer EB (Qiagen) and stored at –20 °C. The quality and concentration of the cDNA libraries generated was assessed using a High-Sensitivity Bioanalyzer (Agilent).

Illumina library preparation and sequencing

1.25 µl of cDNA was used for tagmentation reaction carried out with Nextera XT DNA Sample Preparation kit (Illumina) according to the manufacturer's instructions, but using one-fourth of the stipulated volumes. Purification of the product was implemented with a 1:1 ratio of AMPure XP beads, with a final elution in 17.5 µl in resuspension buffer provided from the Nextera kit (Illumina). Samples were loaded on a High-Sensitivity DNA chip (Agilent Technologies) to check the size and quality of the indexed library, and the concentration was

measured with Qubit High-Sensitivity DNA kit (Invitrogen). Barcoded libraries were sequenced using an Illumina Nextseq 500.

RNA-seq data analysis

Quality analysis of RNA-seq reads was performed using the FastQC tool, and Trimmomatic software (version 0.38) was used to discard low quality reads. >99% of reads in each of the 3 replicates of both the uninfected and infected samples achieved an average quality score of 20 or above. These were mapped to the human reference genome Ensembl GRCh37 using the open-source software tool HISAT2 (version 2.1.0). Transcripts were assembled from the reads using StringTie (version 1.3.4). Fragments per kilobase of transcript per million mapped reads (FPKM) were obtained for each transcript, with an average FPKM value calculated from 3 biological replicates. Only transcripts with a FPKM value >0.5 were considered. Transcripts were subsequently classified in to 3 groups: Group 1: known transcripts that have Ensembl protein ID and have protein sequences; Group 2: known transcripts that do not have an Ensembl protein ID/sequence; Group 3: novel transcripts – transcripts with a novel combination of known exons or novel splice junctions.

RMA-S peptide-HLA binding assay

RMA-S cell lines were pre-cultured at 26°C for 18 hr, to allow accumulation of empty HLA-I molecules on the cell surface. Each RMA-S cell line was incubated with increasing concentrations of the relevant peptides at 26°C for 1 hr, then incubated at 37°C for another 3 hr. After incubation, cells were stained with anti-HLA-I α3 domain mAb TP25.99 (5) or (for the C*03:03 RMA-S cell line) the anti-HLA-C DT9 antibody (a gift from Prof. Mary Carrington, NIH). FITC-conjugated mouse immunoglobulin G (IgG) (Jackson ImmunoResearch Laboratories) was used as the secondary antibody for mean fluorescence

intensity (MFI) readouts. The surface expression of HLA-I molecules was measured by flow cytometry (BD FACSCanto II). The relative HLA expression index was calculated as: (MFI of RMA-S cells pre-pulsed with peptide / MFI of RMA-S cells without peptide pulsing).

Sequencing of HIV PCR amplicons

PCR amplicons were fragmented with Nextera tagmentation reagents (Illumina Inc., San Diego, CA) and individually barcoded. Barcoded libraries were purified, quantified, and sequenced using the Illumina MiSeq 300 cycle Nano Kit v2 (Illumina Inc., San Diego, CA). Raw sequence reads were trimmed of adapter sequence and binned according to barcode. Reads from each amplicon were aligned to the HIV-1 NL4-3 reference using Geneious analysis software (Biomatters Ltd) to generate a consensus sequence. Sequences were examined for mixed bases and amplicons with greater than 25% sequence diversity at any one position were considered mixtures and excluded from further analysis.

References

1. N. Ternette *et al.*, Early Kinetics of the HLA Class I-Associated Peptidome of MVA.HIVconsv-Infected Cells. *J Virol* **89**, 5760-5771 (2015).
2. J. F. Salazar-Gonzalez *et al.*, Deciphering human immunodeficiency virus type 1 transmission and early envelope diversification by single-genome amplification and sequencing. *J Virol* **82**, 3952-3970 (2008).
3. H. Yang *et al.*, Improved quantification of HIV-1-infected CD4+ T cells using an optimised method of intracellular HIV-1 gag p24 antigen detection. *J Immunol Methods* **391**, 174-178 (2013).
4. S. Picelli *et al.*, Smart-seq2 for sensitive full-length transcriptome profiling in single cells. *Nat Methods* **10**, 1096-1098 (2013).

5. M. Tanabe, M. Sekimata, S. Ferrone, M. Takiguchi, Structural and functional analysis of monomorphic determinants recognized by monoclonal antibodies reacting with the HLA class I alpha 3 domain. *J Immunol* **148**, 3202-3209 (1992).

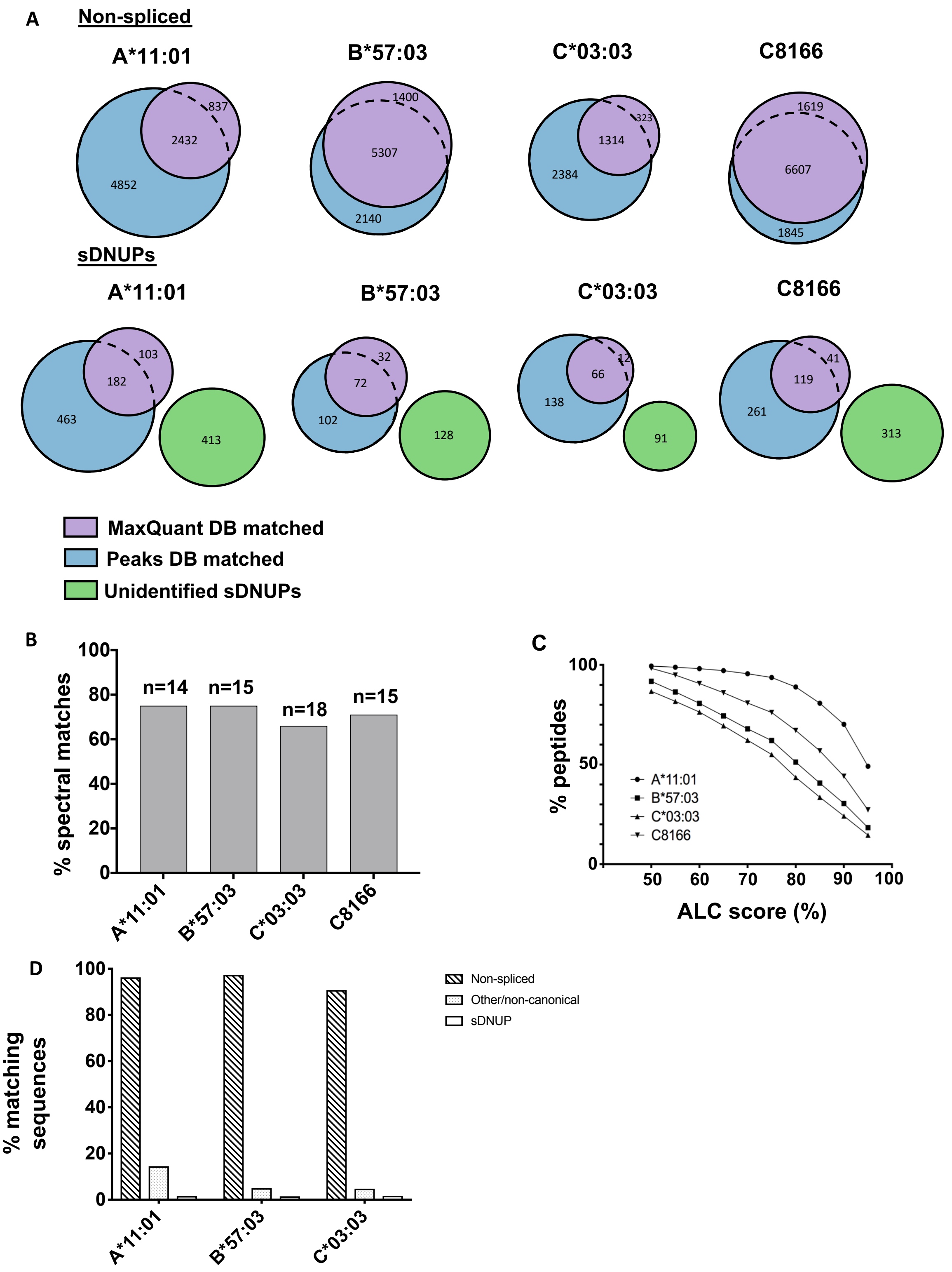

Figure S1: Effect of search engine and *post hoc* spliced protein database validation on sDNUP identification

(A) Area-proportional Venn diagrams showing database-matched non-spliced peptides and sDNUPs identified by PEAKS (blue) and MaxQuant (purple). sDNUPs identified in our *de novo* sequencing workflow by PEAKS which were not subsequently identified following artificial spliced protein database validation are in green. Using PEAKS, 29-42% of DNUPs initially assigned by our workflow as sDNUPs were not identified following implementation of *post hoc* database validation.

(B) Proportion of spectrally matched sDNUPs discovered in our workflow that were not identified following *post hoc* database validation with artificial spliced proteins. The total numbers of peptides included in each dataset (n) are indicated above each bar. Following spectral matching of 62 sDNUPs that were unidentified (across all 4 datasets), ~70-80% matched with high confidence to spectral of synthetic peptide standards.

(C) Cumulative ALC score distributions of *de novo* sequences assigned to database-matched non-spliced peptides are allele-dependent.

(D) Percentage of peptide sequences matching to three-frame translation of CD4.221 RNA transcripts.

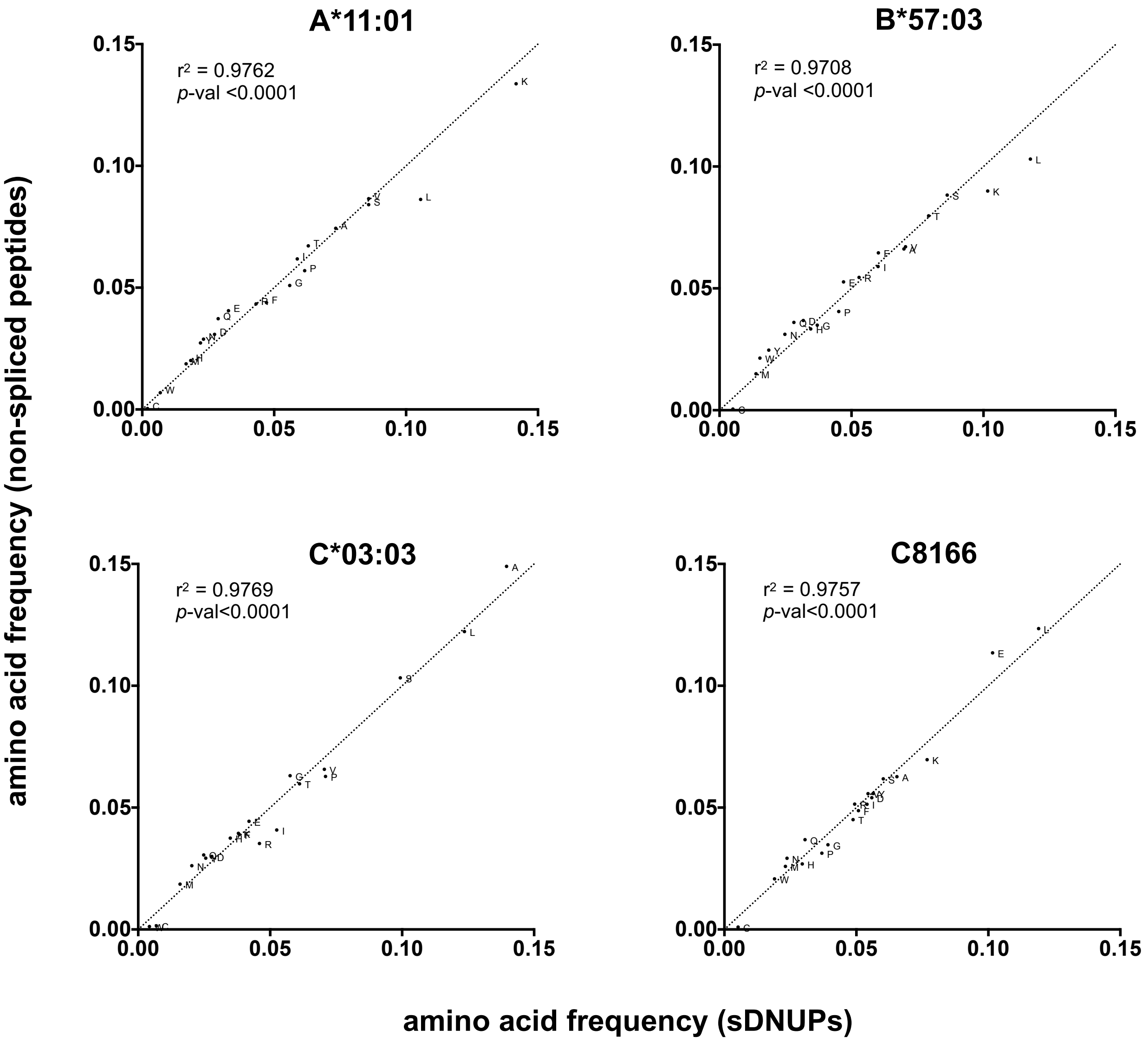

Figure S2: Amino acid frequencies in unique spliced and non-spliced peptides
Amino acid frequency comparisons of unique sDNUP and non-spliced peptides indicate a strong correlation between the proportions of each amino acid residue in the two sets of peptides within a given HLA-I-bound peptide repertoire.

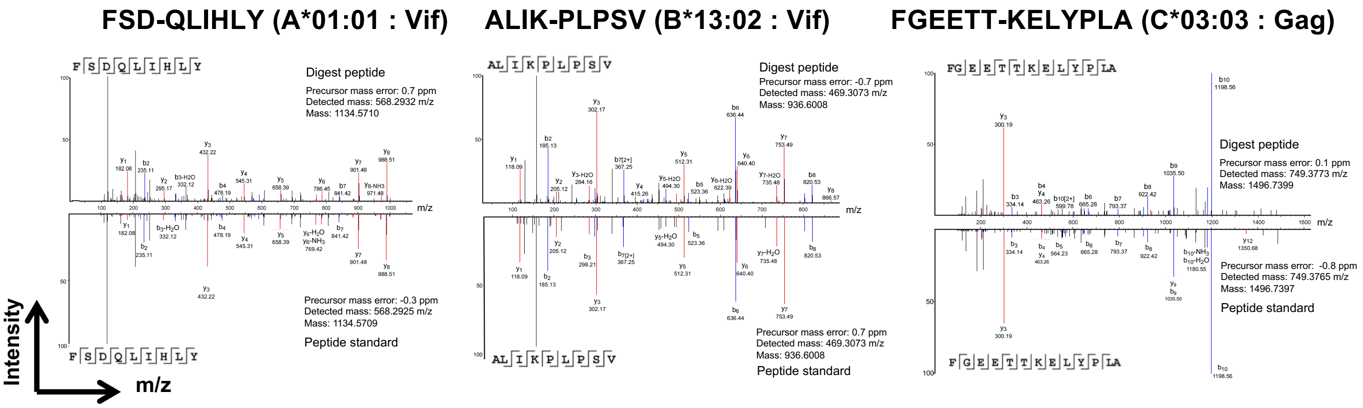

Figure S3: Spectral matching of *in vitro* generated HIV-1 spliced peptides

(A) Spectral matching of HIV-1 IIIB (FSD-QLIHLY) or NL4.3-derived (ALIK-PLPSV and FGEETT-KELYPA) spliced peptides following *in vitro* proteasomal digests of precursor peptides (with corresponding synthetic peptide standards).

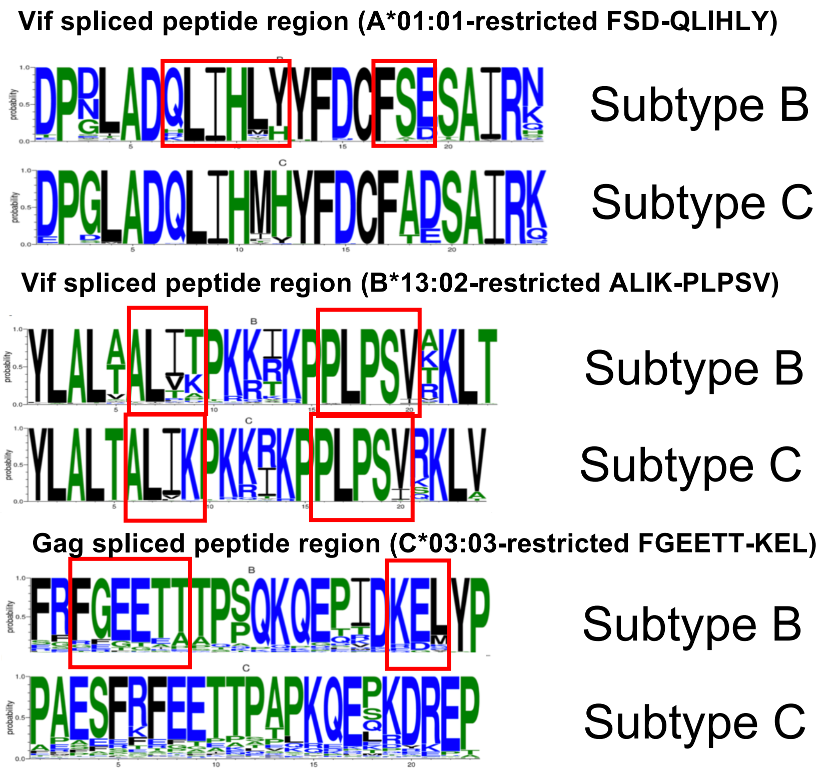

Figure S4: Subtype consensus sequences of confirmed HIV-1-derived spliced peptides

Filtered web alignment of the HIV-1 subtype B and C sequence logos for the regions containing the relevant experimentally validated HIV-1 IIIB- or NL4.3-derived spliced epitopes (highlighted in red boxes) generated from all circulating subtype B and C viral sequences submitted to the Los Alamos National Laboratory database resource in July 2017. Logos were generated using LANL QuickAlign.
