# Supplementary material for: Contribution of proteasome-catalyzed peptide cis-splicing to viral targeting by CD8+ T cells in HIV-1 infection
Source: Proc Natl Acad Sci U S A. 2019 Nov 20;116(49):24748–59. doi: 10.1073/pnas.1911622116 (PMC6900506; doi:10.1073/pnas.1911622116)
Supplement: Supplementary File [file pnas.1911622116.sd03.pdf]

Supporting Dataset S3

Spectral matching of non-spliced and sDNUPs

www.pnas.org/cgi/doi/10.1073/pnas.1911622116

ALC 80%-99% sDNUPs – spectral matches

A*11:01

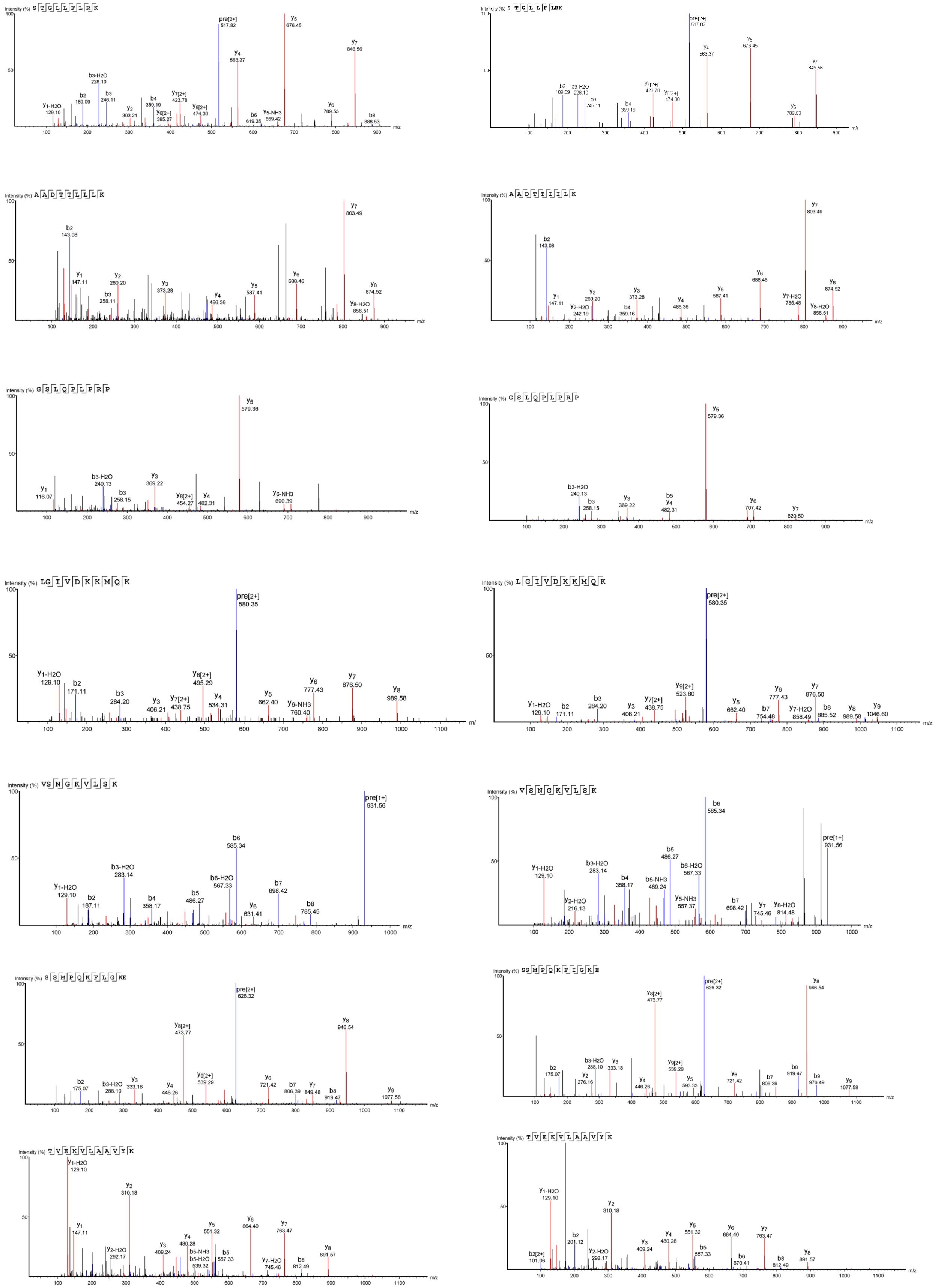

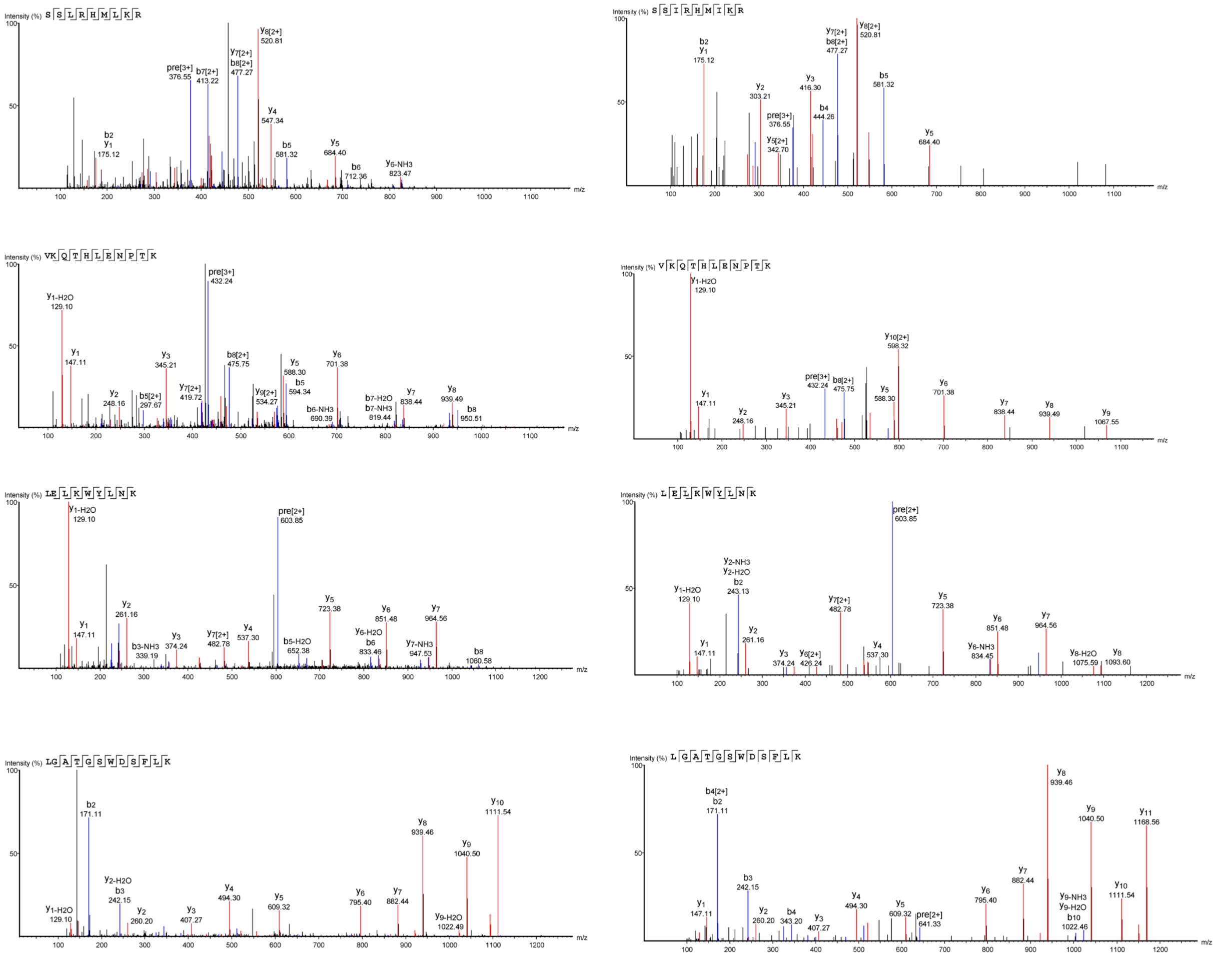

SSLRHMLKR
SSIRHMIKR
VKQTHLENPTK
VKQTHLENPTK
LELKWYLNK
LELKWYLNK
LGATGSWDSFLK
LGATGSWDSFLK

B*57:03

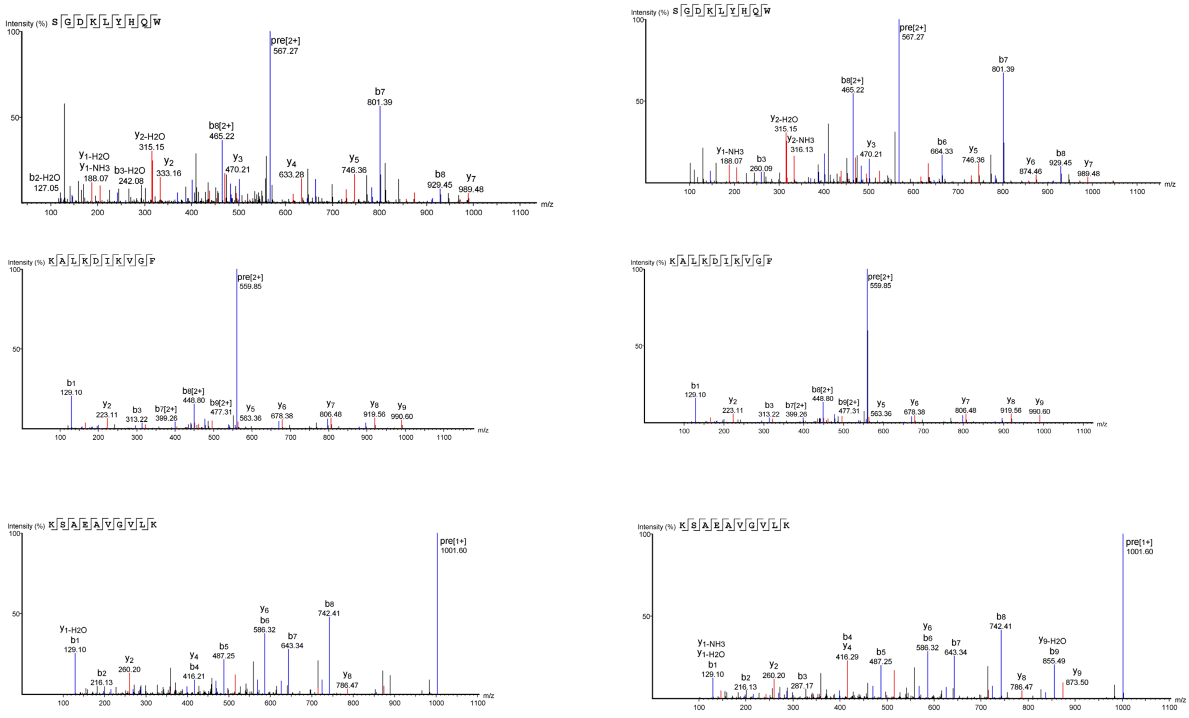

SGDKLYHQW
SGDKLYHQW
KALKDIKVGF
KALKDIKVGF
KSAEAVGVLK
KSAEAVGVLK

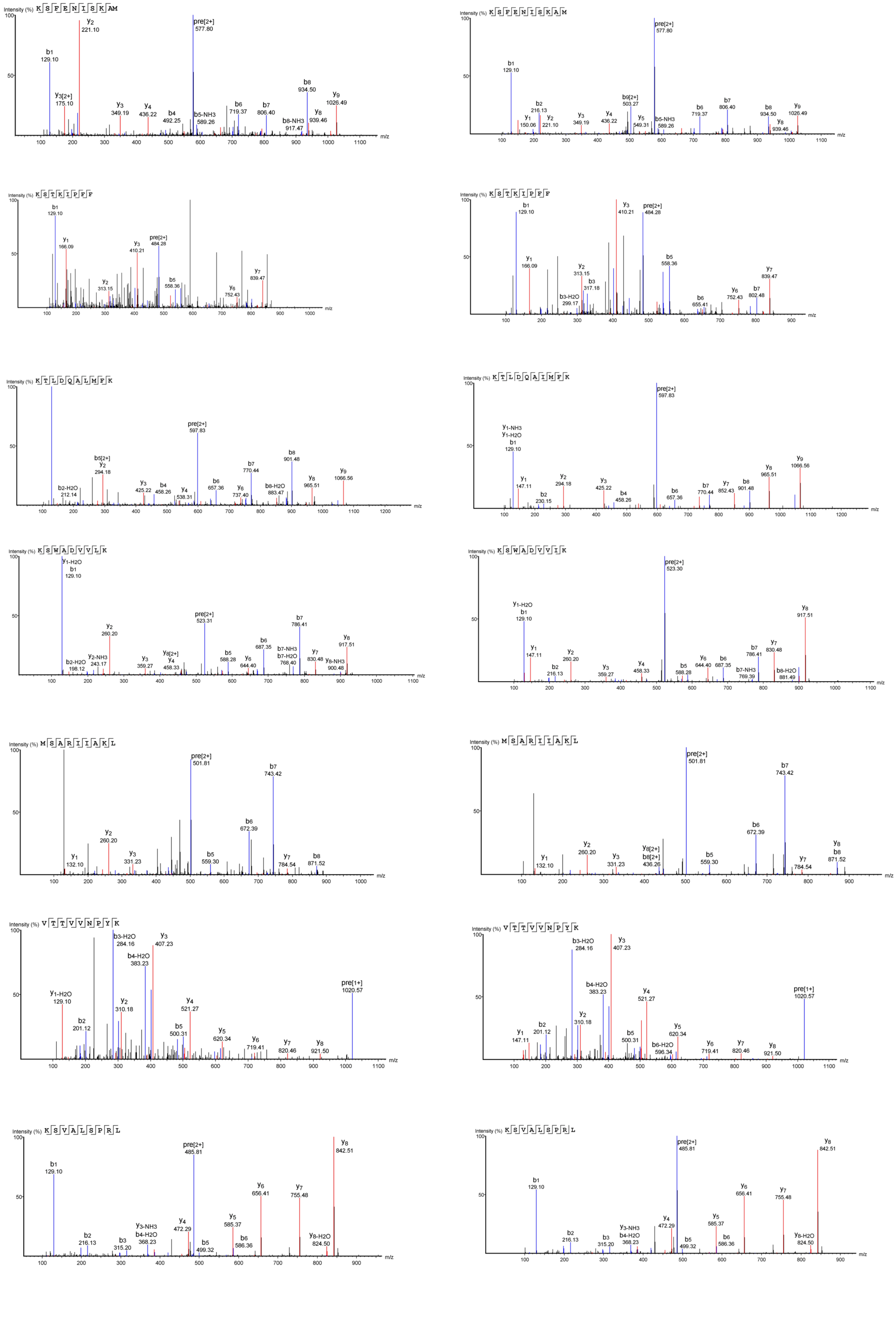
Intensity (%) KSFENISKAM
pre[2+] 577.80
y2 221.10
b1 129.10
y3[2+] 175.10
y3 349.19
y4 436.22
b4 492.25
b5-NH3 589.26
b6 719.37
b7 806.40
b8-NH3 917.47
b8 934.50
y8 939.46
y9 1026.49
m/z
Intensity (%) KSFENISKAM
pre[2+] 577.80
b1 129.10
b2 216.13
y1 150.06
y2 221.10
y3 349.19
y4 436.22
b9[2+] 503.27
y5 549.31
b5-NH3 589.26
b6 719.37
b7 806.40
b8 934.50
y8 939.46
y9 1026.49
m/z
Intensity (%) KSTKIPFF
b1 129.10
y1 166.09
y3 410.21
pre[2+] 484.28
y2 313.15
b5 558.36
y6 752.43
y7 839.47
m/z
Intensity (%) KSTKIPFF
b1 129.10
y1 166.09
y3 410.21
pre[2+] 484.28
y2 313.15
b3 317.18
b3-H2O 299.17
b5 558.36
b6 655.41
y6 752.43
b7 802.48
y7 839.47
m/z
Intensity (%) KTLDQALMFK
b2-H2O 212.14
b5[2+]
y2 294.18
y3 425.22
b4 458.26
y4 538.31
pre[2+] 597.83
b6 657.36
y6 737.40
b7 770.44
b8-H2O 883.47
b8 901.48
y8 965.51
y9 1066.56
m/z
Intensity (%) KTLDQALMFK
pre[2+] 597.83
y1-NH3
y1-H2O
b1 129.10
y1 147.11
b2 230.15
y2 294.18
y3 425.22
b4 458.26
b6 657.36
b7 770.44
y7 852.43
b8 901.48
y8 965.51
y9 1066.56
m/z
Intensity (%) KSWADVVLK
y1-H2O
b1 129.10
b2-H2O 198.12
y2-NH3 243.17
y2 260.20
y3 359.27
y8[2+]
y4 458.33
pre[2+] 523.31
b5 588.28
y6 644.40
b6 687.35
b7-NH3
b7-H2O 768.40
b7 786.41
y7 830.48
y8-NH3 900.48
y8 917.51
m/z
Intensity (%) KSWADVVLK
pre[2+] 523.30
y1-H2O
b1 129.10
y1 147.11
b2 216.13
y2 260.20
y3 359.27
y4 458.33
b5 588.28
y6 644.40
b6 687.35
b7-NH3 769.39
b7 786.41
y7 830.48
b8-H2O 881.49
y8 917.51
m/z
Intensity (%) MSARIIAKL
y1 132.10
y2 260.20
y3 331.23
pre[2+] 501.81
b5 559.30
b6 672.39
b7 743.42
y7 784.54
b8 871.52
m/z
Intensity (%) MSARIIAKL
pre[2+] 501.81
y1 132.10
y2 260.20
y3 331.23
y8[2+]
b8[2+] 436.26
b5 559.30
b6 672.39
b7 743.42
y7 784.54
y8
b8 871.52
m/z
Intensity (%) VTTVVNPYK
y1-H2O 129.10
b2 201.12
b3-H2O 284.16
y2 310.18
b4-H2O 383.23
y3 407.23
b5 500.31
y4 521.27
y5 620.34
y6 719.41
y7 820.46
y8 921.50
pre[1+] 1020.57
m/z
Intensity (%) VTTVVNPYK
y1 147.11
b2 201.12
b3-H2O 284.16
y2 310.18
b4-H2O 383.23
y3 407.23
b5 500.31
y4 521.27
b6-H2O 596.34
y5 620.34
y6 719.41
y7 820.46
y8 921.50
pre[1+] 1020.57
m/z
Intensity (%) KSVALSPRL
b1 129.10
b2 216.13
b3 315.20
y3-NH3
b4-H2O 368.23
y4 472.29
pre[2+] 485.81
b5 499.32
y5 585.37
b6 586.36
y6 656.41
y7 755.48
y8-H2O 824.50
y8 842.51
m/z
Intensity (%) KSVALSPRL
b1 129.10
b2 216.13
b3 315.20
y3-NH3
b4-H2O 368.23
y4 472.29
pre[2+] 485.81
b5 499.32
y5 585.37
b6 586.36
y6 656.41
y7 755.48
y8-H2O 824.50
y8 842.51
m/z

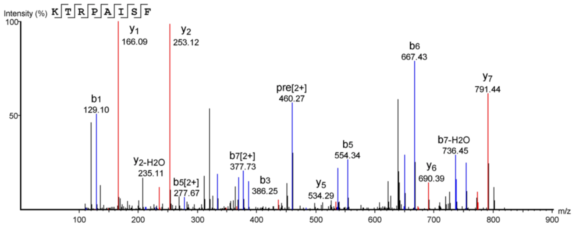
Intensity (%) KTRPAISF
y1 166.09
y2 253.12
b1 129.10
y2-H2O 235.11
b5[2+] 277.67
b7[2+] 377.73
b3 386.25
pre[2+] 460.27
y5 534.29
b5 554.34
b6 667.43
y6 690.39
b7-H2O 736.45
y7 791.44
m/z

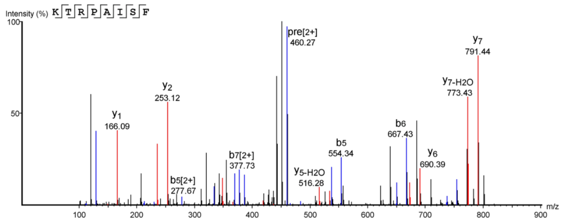
Intensity (%) KTRPAISF
y1 166.09
y2 253.12
b5[2+] 277.67
b7[2+] 377.73
pre[2+] 460.27
y5-H2O 516.28
b5 554.34
b6 667.43
y6 690.39
y7-H2O 773.43
y7 791.44
m/z

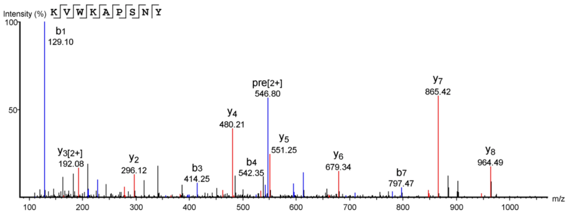
Intensity (%) KVWKAPSNY
b1 129.10
y3[2+] 192.08
y2 296.12
b3 414.25
y4 480.21
b4 542.35
pre[2+] 546.80
y5 551.25
y6 679.34
b7 797.47
y7 865.42
y8 964.49
m/z

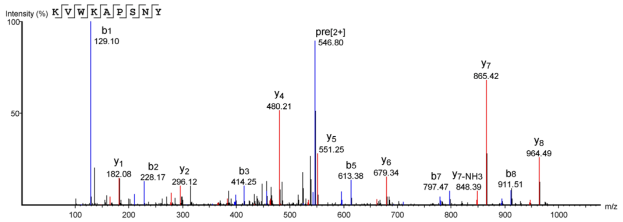
Intensity (%) KVWKAPSNY
b1 129.10
y1 182.08
b2 228.17
y2 296.12
b3 414.25
y4 480.21
pre[2+] 546.80
y5 551.25
b5 613.38
y6 679.34
b7 797.47
y7-NH3 848.39
y7 865.42
b8 911.51
y8 964.49
m/z

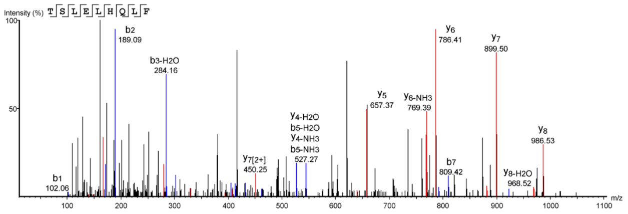
Intensity (%) TSLELHQLF
b1 102.06
b2 189.09
b3-H2O 284.16
y7[2+] 450.25
y4-H2O
b5-H2O
y4-NH3
b5-NH3 527.27
y5 657.37
y6-NH3 769.39
y6 786.41
b7 809.42
y7 899.50
y8-H2O 968.52
y8 986.53
m/z

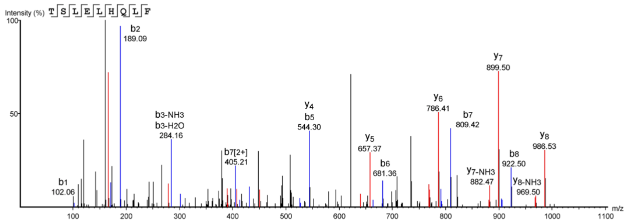
Intensity (%) TSLELHQLF
b1 102.06
b2 189.09
b3-NH3
b3-H2O 284.16
b7[2+] 405.21
y4
b5 544.30
y5 657.37
b6 681.36
y6 786.41
b7 809.42
y7-NH3 882.47
y7 899.50
b8 922.50
y8-NH3 969.50
y8 986.53
m/z

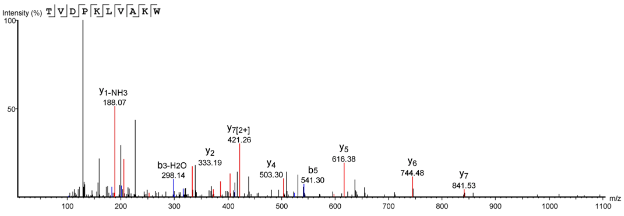
Intensity (%) TVDPKLVAKW
y1-NH3 188.07
b3-H2O 298.14
y2 333.19
y7[2+] 421.26
y4 503.30
b5 541.30
y5 616.38
y6 744.48
y7 841.53
m/z

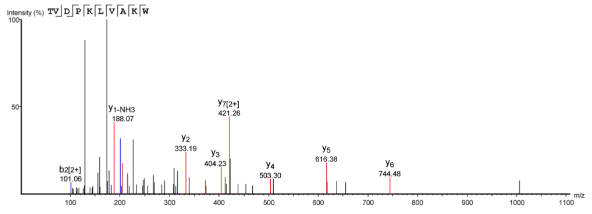
Intensity (%) TVDPKLVAKW
b2[2+] 101.06
y1-NH3 188.07
y2 333.19
y3 404.23
y7[2+] 421.26
y4 503.30
y5 616.38
y6 744.48
m/z

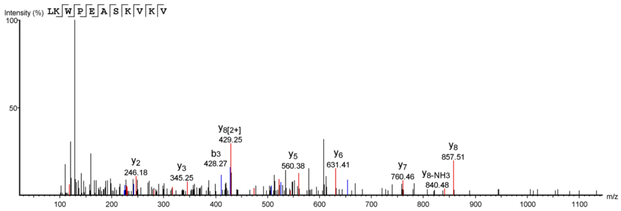
Intensity (%) LKWPEASKVKV
y2 246.18
y3 345.25
b3 428.27
y8[2+] 429.25
y5 560.38
y6 631.41
y7 760.46
y8-NH3 840.48
y8 857.51
m/z

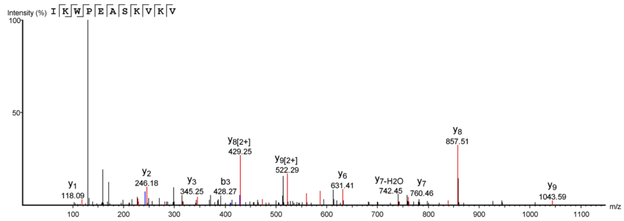
Intensity (%) IKWPEASKVKV
y1 118.09
y2 246.18
y3 345.25
b3 428.27
y8[2+] 429.25
y9[2+] 522.29
y6 631.41
y7-H2O 742.45
y7 760.46
y8 857.51
y9 1043.59
m/z

C*03:03

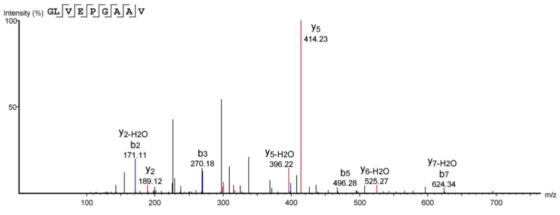
Intensity (%) GLVEPGAAV
y2-H2O
b2 171.11
y2 189.12
b3 270.18
y5-H2O 396.22
y5 414.23
b5 496.28
y6-H2O 525.27
y7-H2O
b7 624.34
m/z

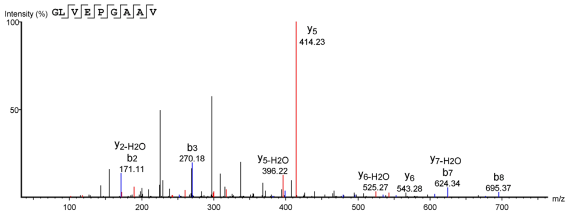
Intensity (%) GLVEPGAAV
y2-H2O
b2 171.11
b3 270.18
y5-H2O 396.22
y5 414.23
y6-H2O 525.27
y6 543.28
y7-H2O
b7 624.34
b8 695.37
m/z

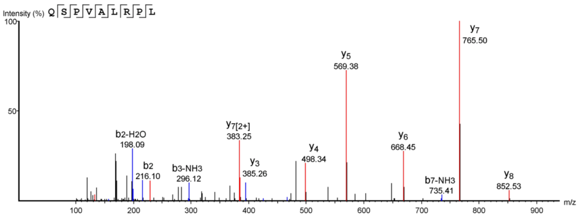
Intensity (%) QSPVALRPL
b2-H2O 198.09
b2 216.10
b3-NH3 296.12
y7[2+] 383.25
y3 385.26
y4 498.34
y5 569.38
y6 668.45
b7-NH3 735.41
y7 765.50
y8 852.53
m/z

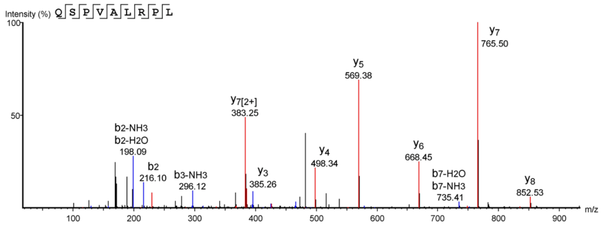
Intensity (%) QSPVALRPL
b2-NH3
b2-H2O 198.09
b2 216.10
b3-NH3 296.12
y7[2+] 383.25
y3 385.26
y4 498.34
y5 569.38
y6 668.45
b7-H2O
b7-NH3 735.41
y7 765.50
y8 852.53
m/z

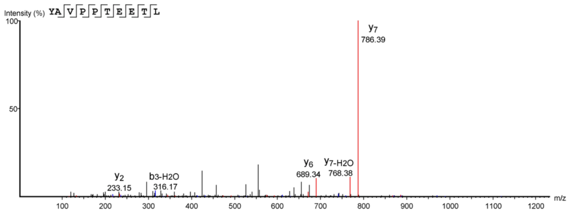

Intensity (%) YAVPPTEETL
y7 786.39
y2 233.15
b3-H2O 316.17
y6 689.34
y7-H2O 768.38
m/z

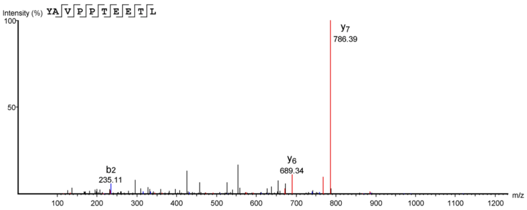

Intensity (%) YAVPPTEETL
y7 786.39
b2 235.11
y6 689.34
m/z

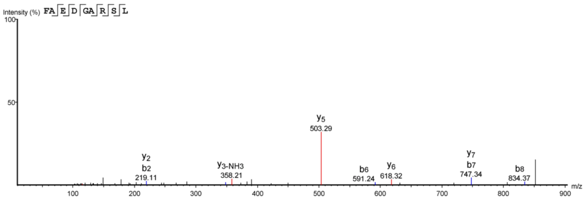

Intensity (%) FAEDGARSL
y2 b2 219.11
y3-NH3 358.21
y5 503.29
b6 591.24
y6 618.32
y7 b7 747.34
b8 834.37
m/z

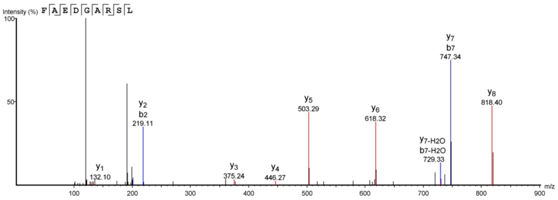

Intensity (%) FAEDGARSL
y7 b7 747.34
y8 818.40
y2 b2 219.11
y5 503.29
y6 618.32
y7-H2O b7-H2O 729.33
y1 132.10
y3 375.24
y4 446.27
m/z

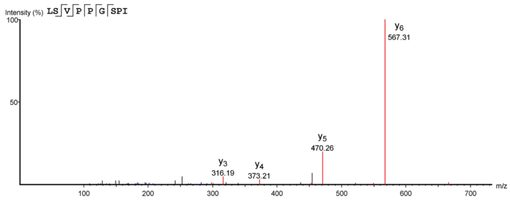

Intensity (%) LSVPPGSPI
y6 567.31
y5 470.26
y3 316.19
y4 373.21
m/z

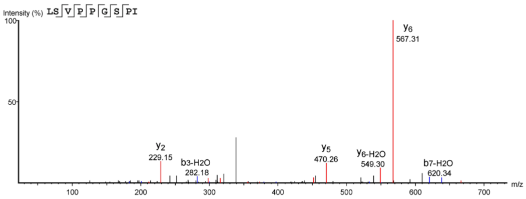

Intensity (%) LSVPPGSPI
y6 567.31
y2 229.15
b3-H2O 282.18
y5 470.26
y6-H2O 549.30
b7-H2O 620.34
m/z

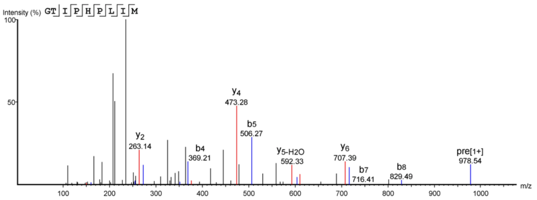

Intensity (%) GTIPHPLIM
y4 473.28
b5 506.27
y2 263.14
b4 369.21
y5-H2O 592.33
y6 707.39
b7 716.41
b8 829.49
pre[1+] 978.54
m/z

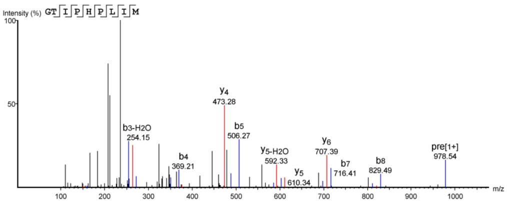

Intensity (%) GTIPHPLIM
y4 473.28
b5 506.27
b3-H2O 254.15
b4 369.21
y5-H2O 592.33
y5 610.34
y6 707.39
b7 716.41
b8 829.49
pre[1+] 978.54
m/z

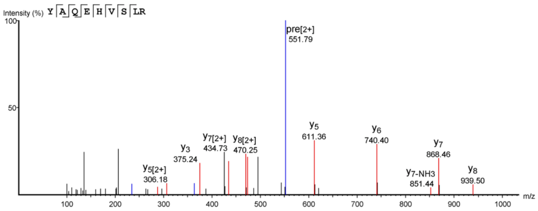

Intensity (%) YAQEHVSLR
pre[2+] 551.79
y5 611.36
y6 740.40
y3 375.24
y7[2+] 434.73
y8[2+] 470.25
y5[2+] 306.18
y7 868.46
y7-NH3 851.44
y8 939.50
m/z

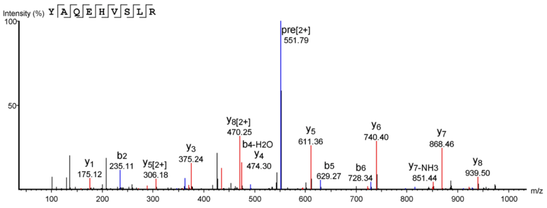

Intensity (%) YAQEHVSLR
pre[2+] 551.79
y8[2+] 470.25
b4-H2O y4 474.30
y5 611.36
y6 740.40
y7 868.46
y1 175.12
b2 235.11
y5[2+] 306.18
y3 375.24
b5 629.27
b6 728.34
y7-NH3 851.44
y8 939.50
m/z

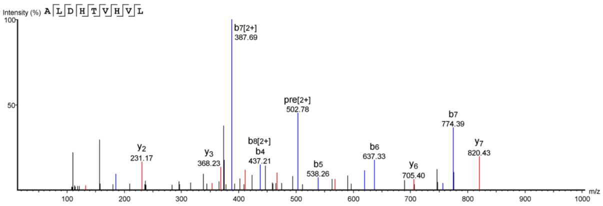

Intensity (%) ALDHTVHVL
b7[2+] 387.69
pre[2+] 502.78
b7 774.39
y2 231.17
y3 368.23
b8[2+] b4 437.21
b5 538.26
b6 637.33
y6 705.40
y7 820.43
m/z

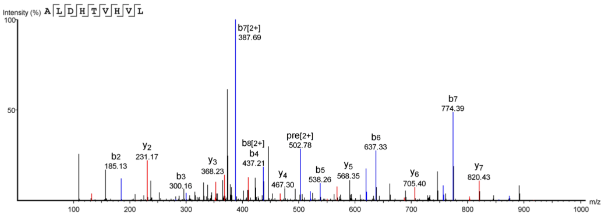

Intensity (%) ALDHTVHVL
b7[2+] 387.69
b7 774.39
pre[2+] 502.78
b6 637.33
b2 185.13
y2 231.17
b3 300.16
y3 368.23
b8[2+] b4 437.21
y4 467.30
b5 538.26
y5 568.35
y6 705.40
y7 820.43
m/z

C8166

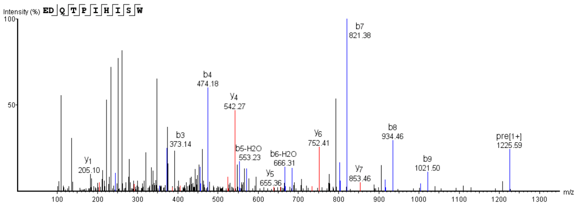

Intensity (%) EDQTPIHISW
b7 821.38
b4 474.18
y4 542.27
b3 373.14
b5-H2O 553.23
b6-H2O 666.31
y6 752.41
b8 934.46
y1 205.10
y5 655.36
y7 853.46
b9 1021.50
pre[1+] 1225.59
m/z

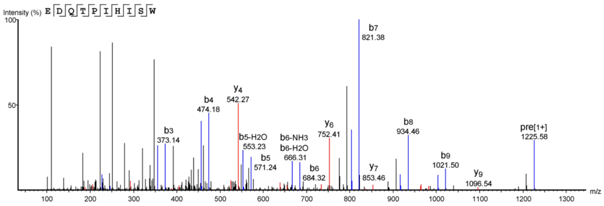

Intensity (%) EDQTPIHISW
b7 821.38
b4 474.18
y4 542.27
b3 373.14
b5-H2O 553.23
b5 571.24
b6-NH3 b6-H2O 666.31
b6 684.32
y6 752.41
b8 934.46
y7 853.46
b9 1021.50
y9 1096.54
pre[1+] 1225.58
m/z

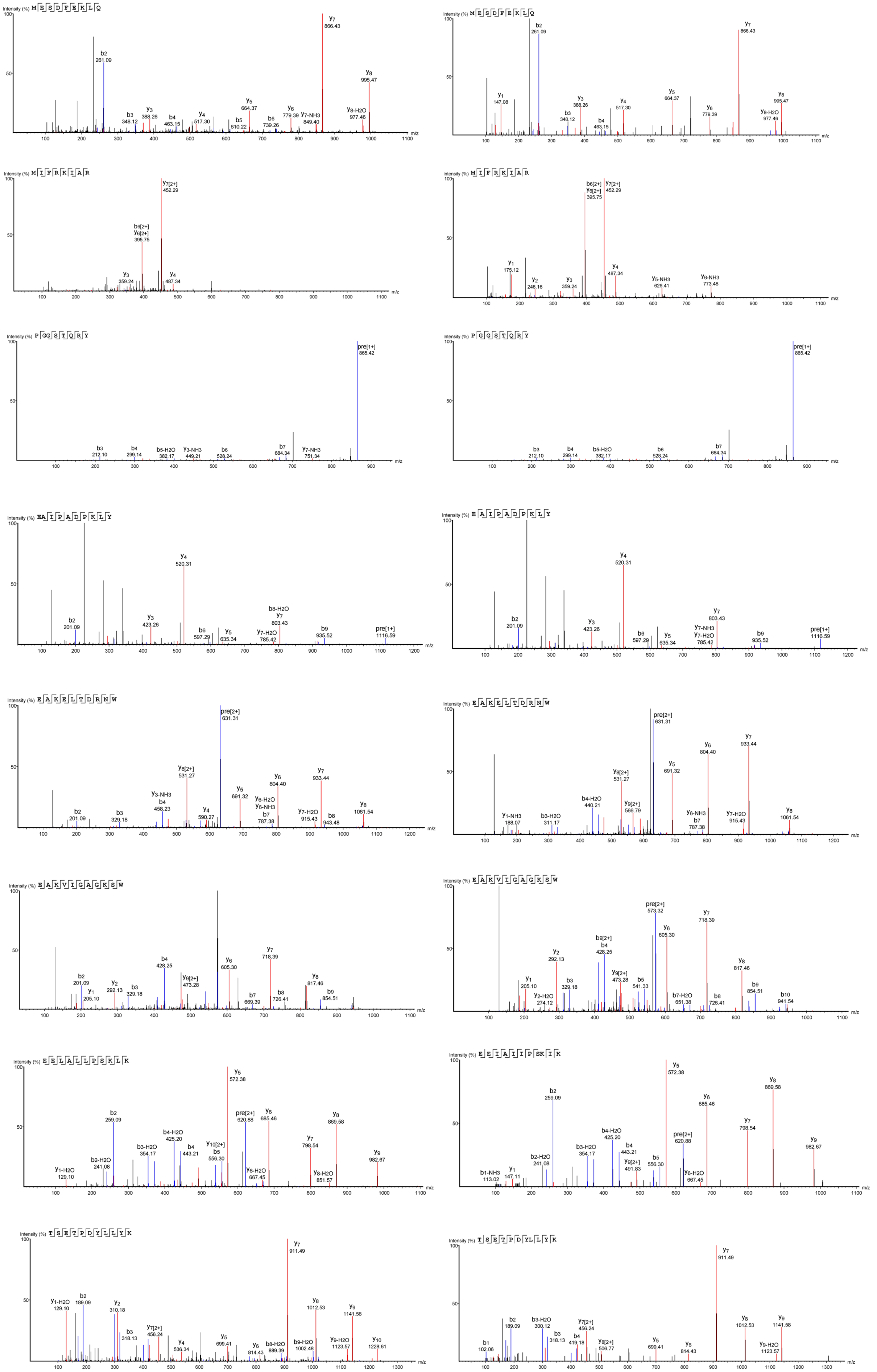

Intensity (%) MESDFEKLQ
Intensity (%) MESDFEKLQ
Intensity (%) MIFRKIAR
Intensity (%) MIFRKIAR
Intensity (%) PGGSTQRY
Intensity (%) PGGSTQRY
Intensity (%) EAIPADPKLY
Intensity (%) EAIPADPKLY
Intensity (%) EAKELTDRNW
Intensity (%) EAKELTDRNW
Intensity (%) EAKVIGAGKSW
Intensity (%) EAKVIGAGKSW
Intensity (%) EELALLPSKLK
Intensity (%) EEIAIIPSKIK
Intensity (%) TSETPDYLLYK
Intensity (%) TSETPDYLLYK
m/z

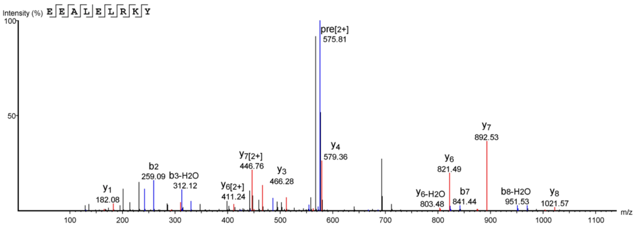
Intensity (%)
E E A L E L R K Y
100
50
pre[2+]
575.81
y4
579.36
y7
892.53
b2
259.09
b3-H2O
312.12
y7[2+]
446.76
y3
466.28
y6
821.49
y1
182.08
y6[2+]
411.24
y6-H2O
803.48
b7
841.44
b8-H2O
951.53
y8
1021.57
100
200
300
400
500
600
700
800
900
1000
1100
m/z

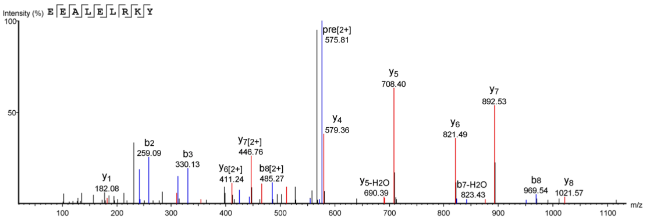
Intensity (%)
E E A L E L R K Y
100
50
pre[2+]
575.81
y5
708.40
y7
892.53
y4
579.36
y6
821.49
b2
259.09
b3
330.13
y7[2+]
446.76
y6[2+]
411.24
b8[2+]
485.27
y1
182.08
y5-H2O
690.39
b7-H2O
823.43
b8
969.54
y8
1021.57
100
200
300
400
500
600
700
800
900
1000
1100
m/z

ALC 80%-99% sDNUPs - non-matched

A*11:01

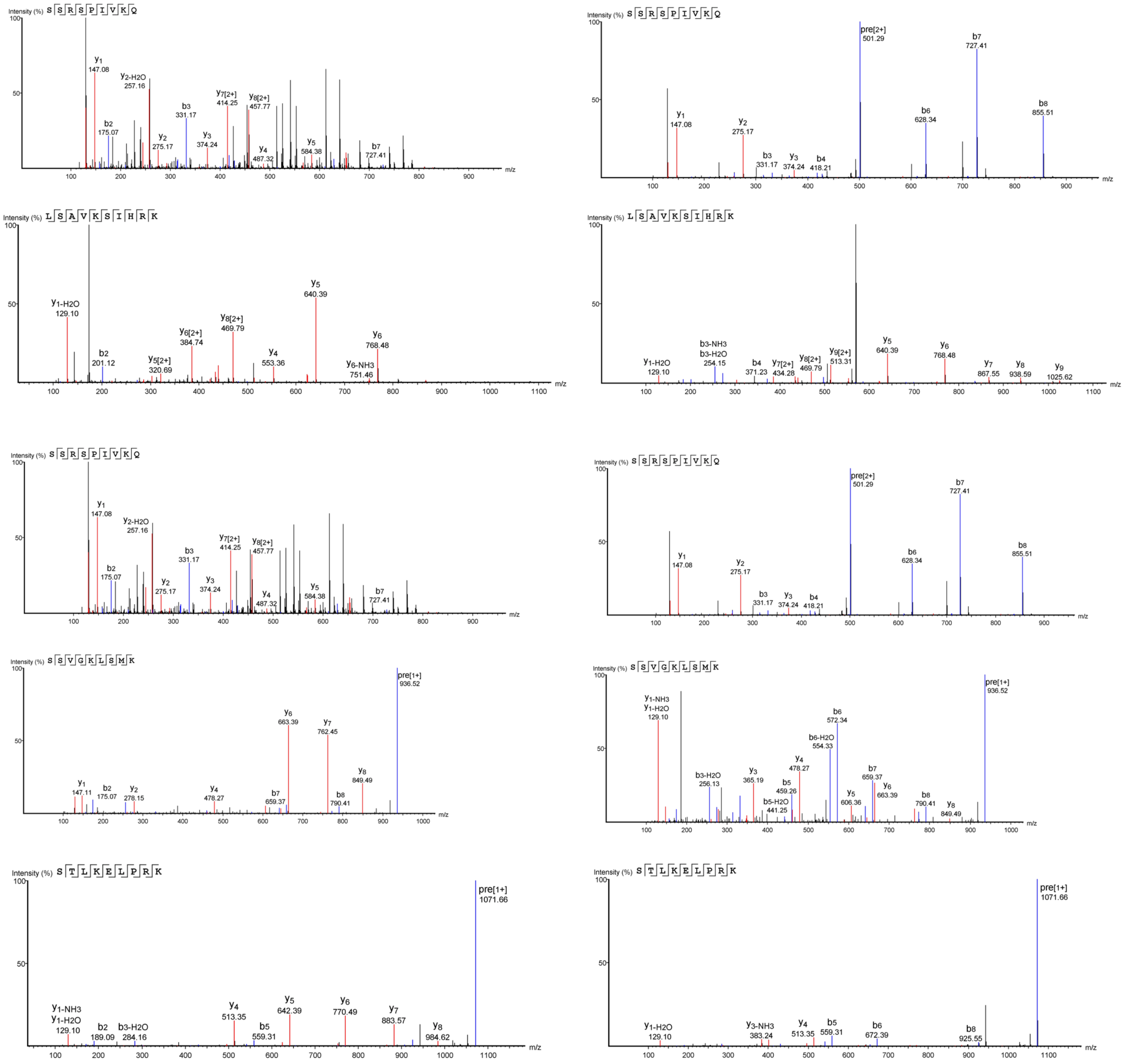

B*57:03

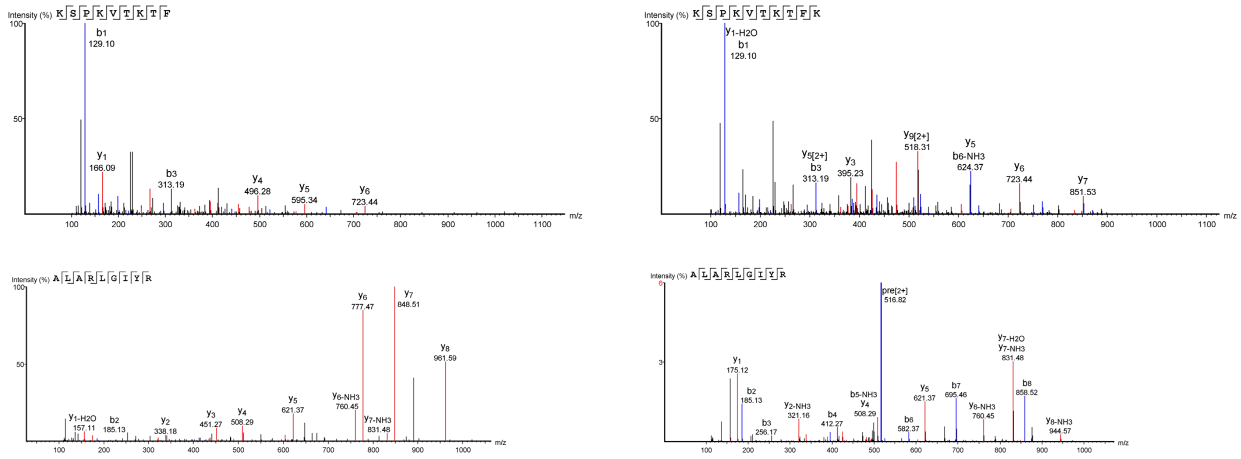

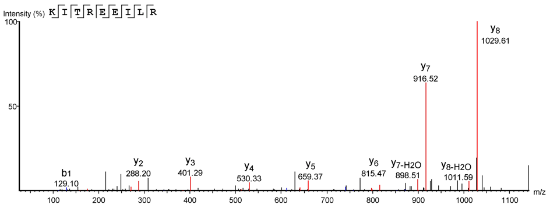
Intensity (%) KITREEILR
b1 129.10
y2 288.20
y3 401.29
y4 530.33
y5 659.37
y6 815.47
y7-H2O 898.51
y7 916.52
y8-H2O 1011.59
y8 1029.61
m/z

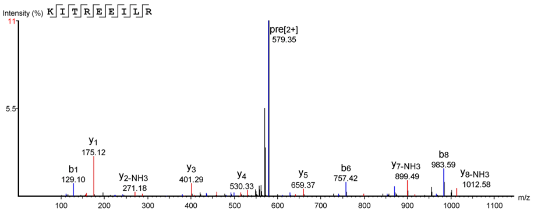
Intensity (%) KITREEILR
pre[2+] 579.35
y1 175.12
b1 129.10
y2-NH3 271.18
y3 401.29
y4 530.33
y5 659.37
b6 757.42
y7-NH3 899.49
b8 983.59
y8-NH3 1012.58
m/z

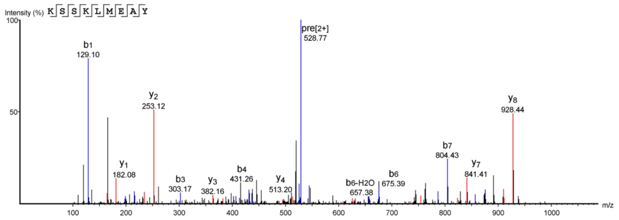
Intensity (%) KSSKLMEAY
b1 129.10
pre[2+] 528.77
y2 253.12
y1 182.08
b3 303.17
y3 382.16
b4 431.26
y4 513.20
b6-H2O 657.38
b6 675.39
b7 804.43
y7 841.41
y8 928.44
m/z

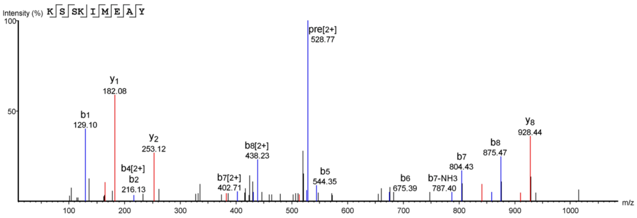
Intensity (%) KSSKIMEAY
pre[2+] 528.77
y1 182.08
b1 129.10
y2 253.12
b4[2+] b2 216.13
b8[2+] 438.23
b7[2+] 402.71
b5 544.35
b6 675.39
b7 804.43
b7-NH3 787.40
b8 875.47
y8 928.44
m/z

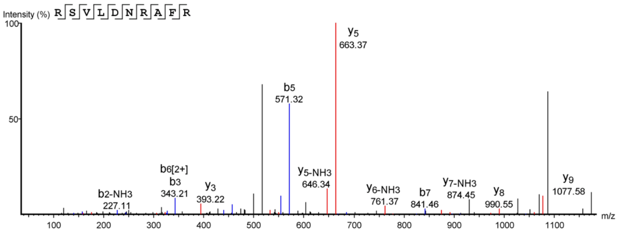
Intensity (%) RSVLDNRAFR
y5 663.37
b5 571.32
b6[2+] b3 343.21
b2-NH3 227.11
y3 393.22
y5-NH3 646.34
y6-NH3 761.37
b7 841.46
y7-NH3 874.45
y8 990.55
y9 1077.58
m/z

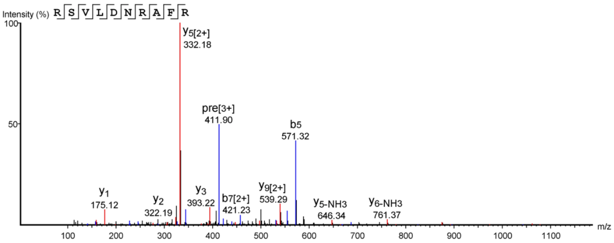
Intensity (%) RSVLDNRAFR
y5[2+] 332.18
pre[3+] 411.90
b5 571.32
y1 175.12
y2 322.19
y3 393.22
b7[2+] 421.23
y9[2+] 539.29
y5-NH3 646.34
y6-NH3 761.37
m/z

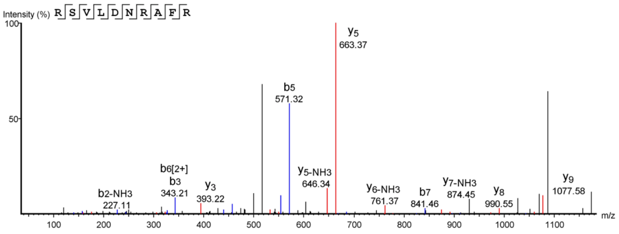
Intensity (%) RSVLDNRAFR
y5 663.37
b5 571.32
b6[2+] b3 343.21
b2-NH3 227.11
y3 393.22
y5-NH3 646.34
y6-NH3 761.37
b7 841.46
y7-NH3 874.45
y8 990.55
y9 1077.58
m/z

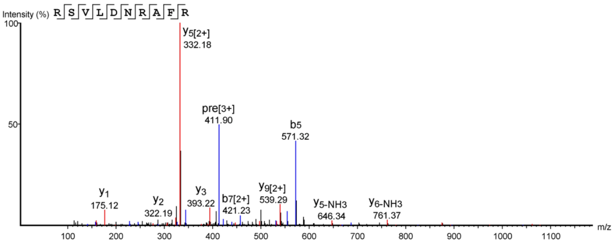
Intensity (%) RSVLDNRAFR
y5[2+] 332.18
pre[3+] 411.90
b5 571.32
y1 175.12
y2 322.19
y3 393.22
b7[2+] 421.23
y9[2+] 539.29
y5-NH3 646.34
y6-NH3 761.37
m/z

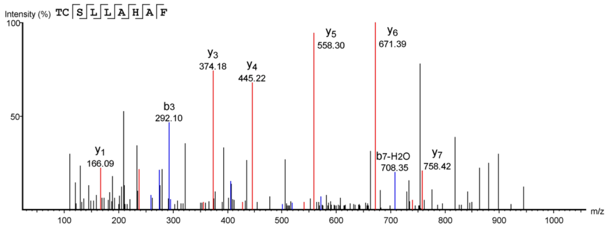
Intensity (%) TCSLLAHAF
y5 558.30
y6 671.39
y3 374.18
y4 445.22
b3 292.10
y1 166.09
b7-H2O 708.35
y7 758.42
m/z

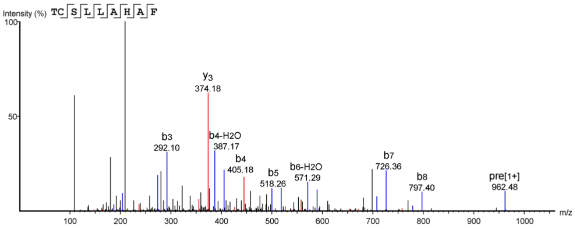
Intensity (%) TCSLLAHAF
y3 374.18
b3 292.10
b4-H2O 387.17
b4 405.18
b5 518.26
b6-H2O 571.29
b7 726.36
b8 797.40
pre[1+] 962.48
m/z

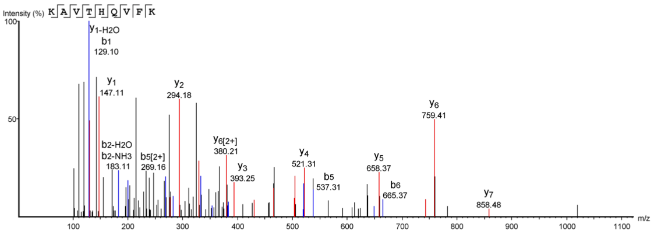
Intensity (%) KAVTHQVFK
y1-H2O b1 129.10
y1 147.11
y2 294.18
b2-H2O b2-NH3 183.11
b5[2+] 269.16
y6[2+] 380.21
y3 393.25
y4 521.31
b5 537.31
y5 658.37
b6 665.37
y6 759.41
y7 858.48
m/z

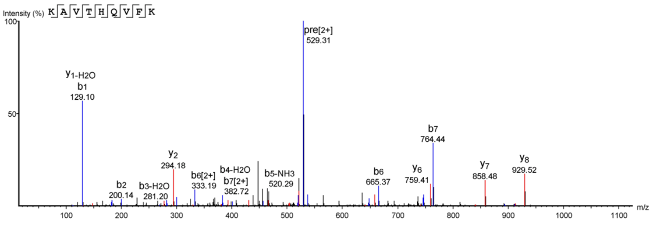
Intensity (%) KAVTHQVFK
pre[2+] 529.31
y1-H2O b1 129.10
b7 764.44
y2 294.18
b2 200.14
b3-H2O 281.20
b6[2+] 333.19
b4-H2O b7[2+] 382.72
b5-NH3 520.29
b6 665.37
y6 759.41
y7 858.48
y8 929.52
m/z

C*03:03

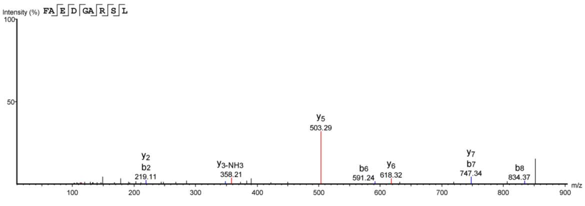
Intensity (%) FAEDGARSL
y5 503.29
y2 b2 219.11
y3-NH3 358.21
b6 591.24
y6 618.32
y7 b7 747.34
b8 834.37
m/z

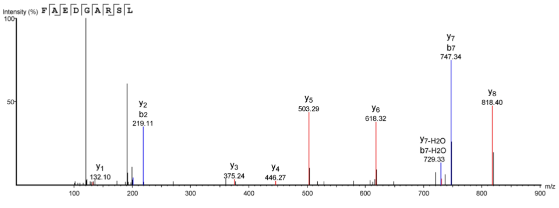
Intensity (%) FAEDGARSL
y7 b7 747.34
y8 818.40
y2 b2 219.11
y5 503.29
y6 618.32
y7-H2O b7-H2O 729.33
y1 132.10
y3 375.24
y4 446.27
m/z

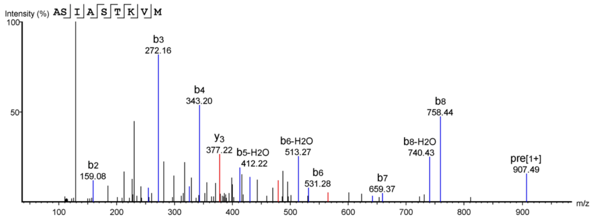
Intensity (%) ASIASTKVM
b2 159.08
b3 272.16
b4 343.20
y3 377.22
b5-H2O 412.22
b6-H2O 513.27
b6 531.28
b7 659.37
b8-H2O 740.43
b8 758.44
pre[1+] 907.49
m/z

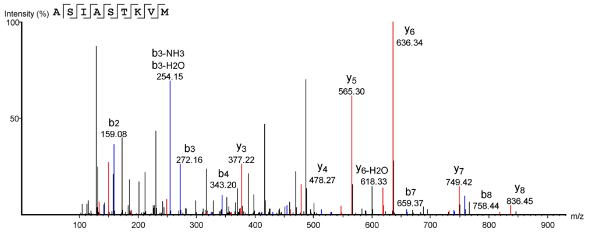
Intensity (%) ASIASTKVM
b2 159.08
b3-NH3
b3-H2O 254.15
b3 272.16
b4 343.20
y3 377.22
y4 478.27
y5 565.30
y6-H2O 618.33
y6 636.34
b7 659.37
y7 749.42
b8 758.44
y8 836.45
m/z

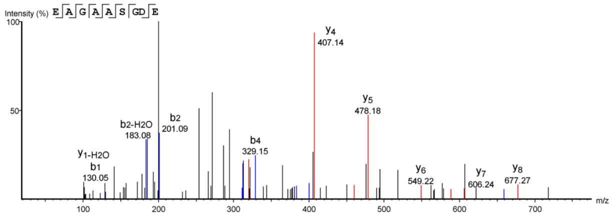
Intensity (%) EAGAASGDE
y1-H2O
b1 130.05
b2-H2O 183.08
b2 201.09
b4 329.15
y4 407.14
y5 478.18
y6 549.22
y7 606.24
y8 677.27
m/z

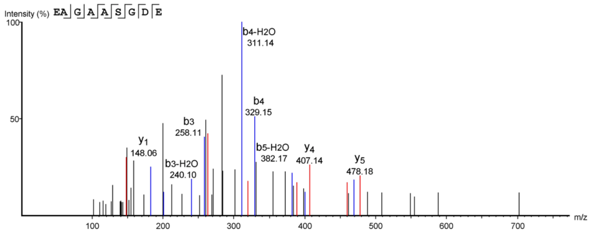
Intensity (%) EAGAASGDE
y1 148.06
b3-H2O 240.10
b3 258.11
b4-H2O 311.14
b4 329.15
b5-H2O 382.17
y4 407.14
y5 478.18
m/z

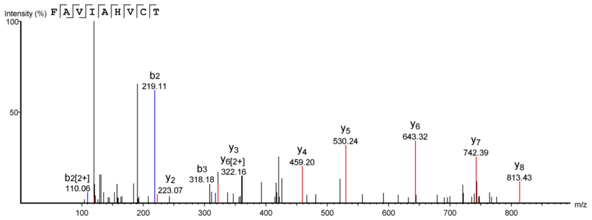
Intensity (%) FAVIAHVCT
b2[2+] 110.06
b2 219.11
y2 223.07
b3 318.18
y3
y6[2+] 322.16
y4 459.20
y5 530.24
y6 643.32
y7 742.39
y8 813.43
m/z

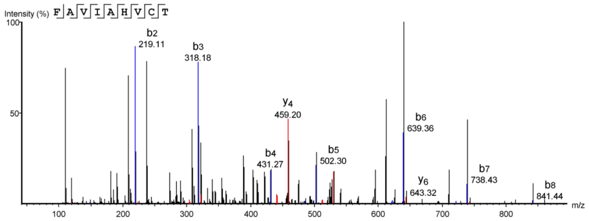
Intensity (%) FAVIAHVCT
b2 219.11
b3 318.18
b4 431.27
y4 459.20
b5 502.30
b6 639.36
y6 643.32
b7 738.43
b8 841.44
m/z

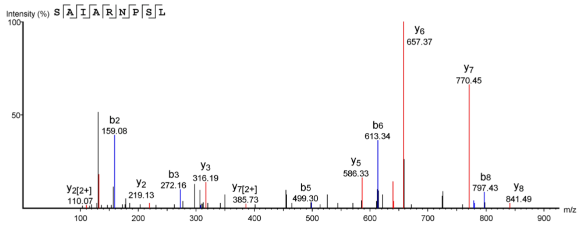
Intensity (%) SAIARNPSL
y2[2+] 110.07
b2 159.08
y2 219.13
b3 272.16
y3 316.19
y7[2+] 385.73
b5 499.30
y5 586.33
b6 613.34
y6 657.37
y7 770.45
b8 797.43
y8 841.49
m/z

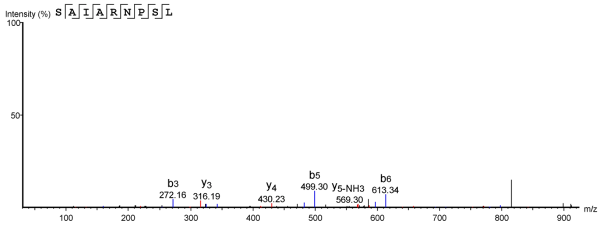
Intensity (%) SAIARNPSL
b3 272.16
y3 316.19
y4 430.23
b5 499.30
y5-NH3 569.30
b6 613.34
m/z

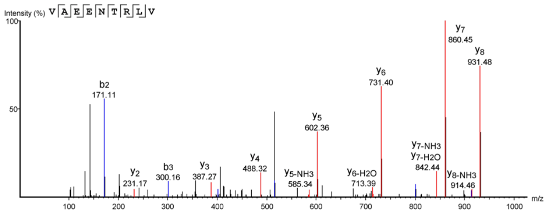
Intensity (%) VAEENTRLV
b2 171.11
y2 231.17
b3 300.16
y3 387.27
y4 488.32
y5-NH3 585.34
y5 602.36
y6-H2O 713.39
y6 731.40
y7-NH3
y7-H2O 842.44
y7 860.45
y8-NH3 914.46
y8 931.48
m/z

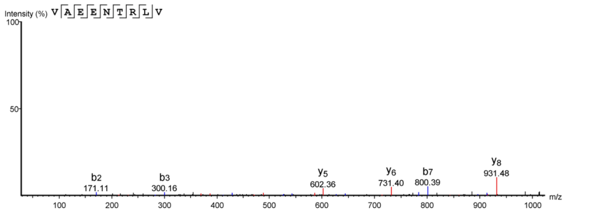
Intensity (%) VAEENTRLV
b2 171.11
b3 300.16
y5 602.36
y6 731.40
b7 800.39
y8 931.48
m/z

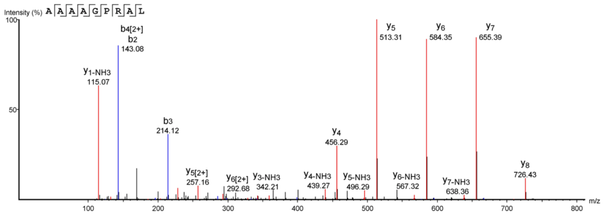
Intensity (%) AAAAGPRAL
y1-NH3 115.07
b4[2+]
b2 143.08
b3 214.12
y5[2+] 257.16
y6[2+] 292.68
y3-NH3 342.21
y4-NH3 439.27
y4 456.29
y5-NH3 496.29
y5 513.31
y6-NH3 567.32
y6 584.35
y7-NH3 638.36
y7 655.39
y8 726.43
m/z

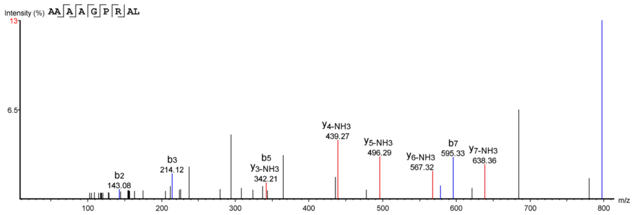
Intensity (%) AAAAGPRAL
b2 143.08
b3 214.12
b5
y3-NH3 342.21
y4-NH3 439.27
y5-NH3 496.29
y6-NH3 567.32
b7 595.33
y7-NH3 638.36
m/z

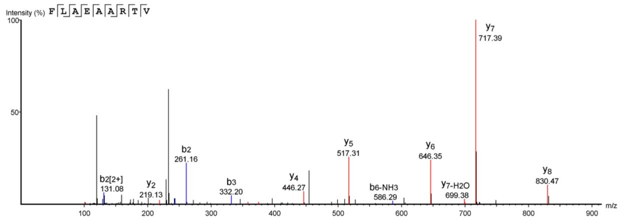
Intensity (%) FLAEAARTV
b2[2+] 131.08
y2 219.13
b2 261.16
b3 332.20
y4 446.27
y5 517.31
b6-NH3 586.29
y6 646.35
y7-H2O 699.38
y7 717.39
y8 830.47
m/z

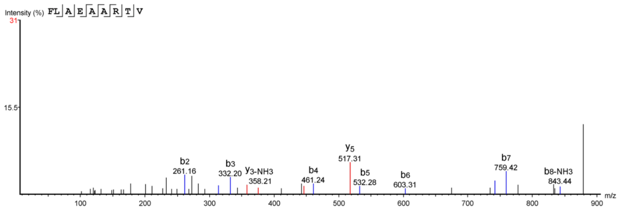
Intensity (%) FLAEAARTV
b2 261.16
b3 332.20
y3-NH3 358.21
b4 461.24
y5 517.31
b5 532.28
b6 603.31
b7 759.42
b8-NH3 843.44
m/z

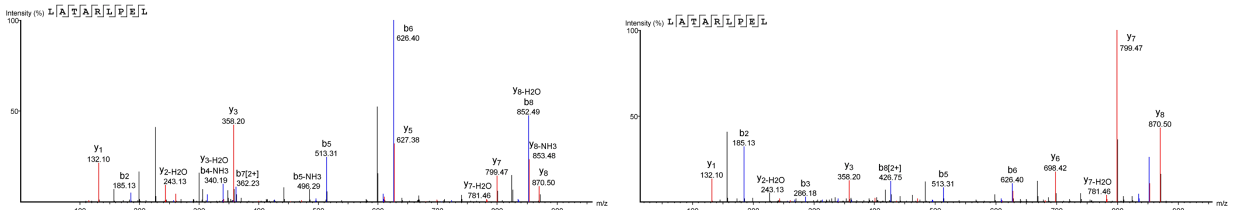
LATARLPEL
LATARLPEL

C8166

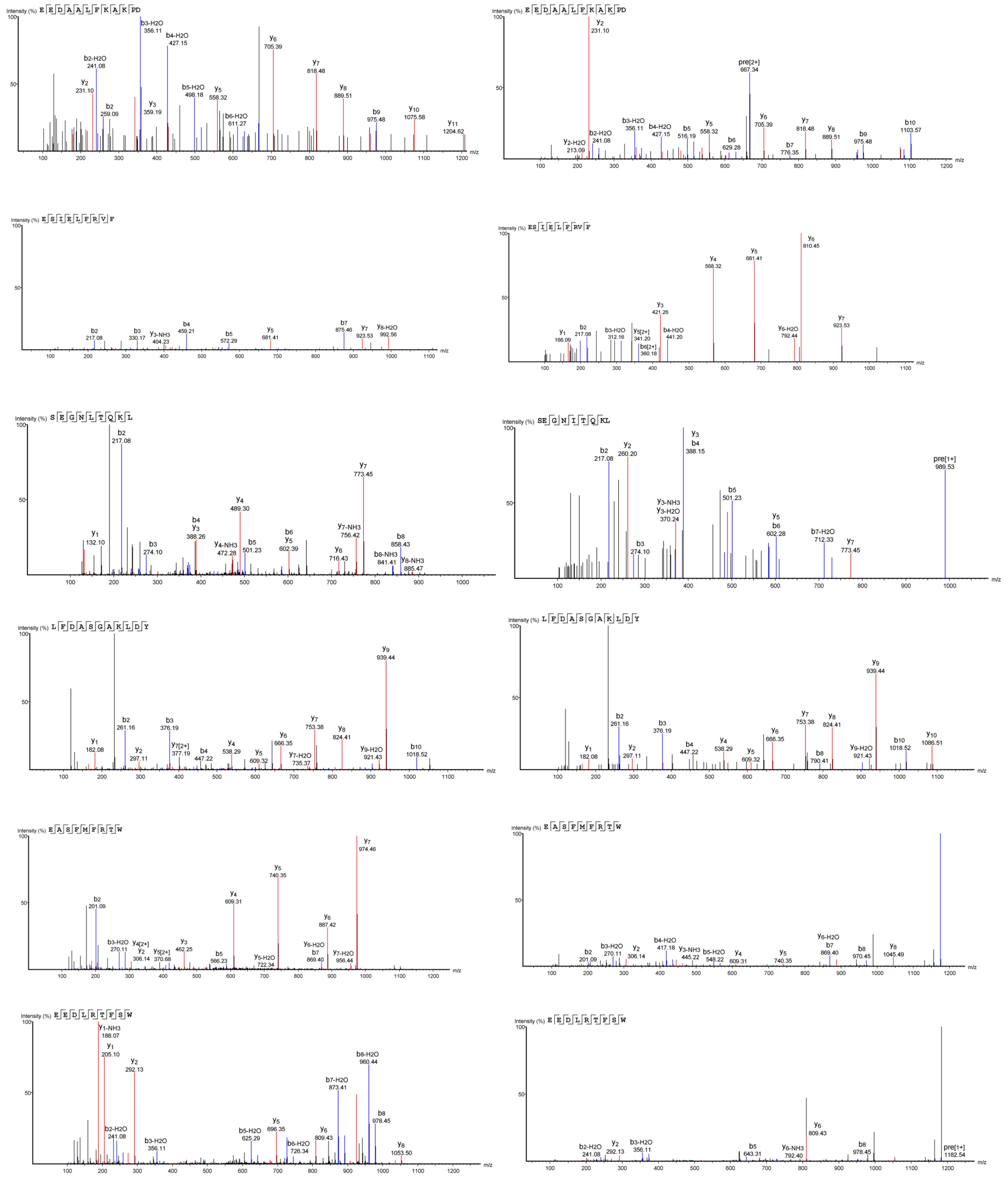
EEDAALFKAKPD
EEDAALFKAKPD
ESIELFRVF
ESIELFRVF
SEGNLTQKL
SEGNITQKL
LFDASGAKLDY
LFDASGAKLDY
EASFMFRTW
EASFMFRTW
EEDLRTFSW
EEDLRTFSW

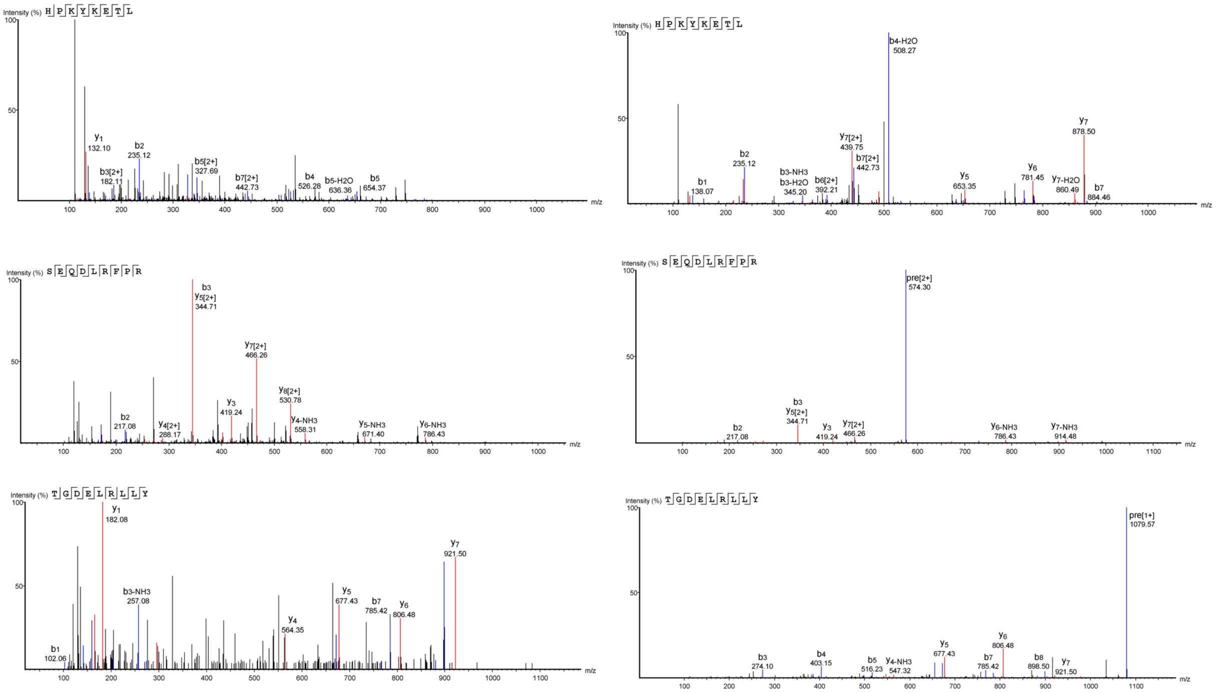

Intensity (%) HPKYKETL
y1 132.10
b2 235.12
b3[2+] 182.11
b5[2+] 327.69
b7[2+] 442.73
b4 526.28
b5-H2O 636.36
b5 654.37
Intensity (%) HPKYKETL
b4-H2O 508.27
b1 138.07
b2 235.12
b3-NH3
b3-H2O 345.20
b6[2+] 392.21
y7[2+] 439.75
b7[2+] 442.73
y5 653.35
y6 781.45
y7-H2O 860.49
y7 878.50
b7 884.46
Intensity (%) SEQDLRFPR
b3
y5[2+] 344.71
y7[2+] 466.26
b2 217.08
y4[2+] 288.17
y3 419.24
y8[2+] 530.78
y4-NH3 558.31
y5-NH3 671.40
y6-NH3 786.43
Intensity (%) SEQDLRFPR
pre[2+] 574.30
b2 217.08
b3
y5[2+] 344.71
y3 419.24
y7[2+] 466.26
y6-NH3 786.43
y7-NH3 914.48
Intensity (%) TGDELRLLY
y1 182.08
b1 102.06
b3-NH3 257.08
y4 564.35
y5 677.43
b7 785.42
y6 806.48
y7 921.50
Intensity (%) TGDELRLLY
pre[1+] 1079.57
b3 274.10
b4 403.15
b5 516.23
y4-NH3 547.32
y5 677.43
b7 785.42
y6 806.48
b8 898.50
y7 921.50
m/z

ALC 50%-79% sDNUPs – spectral matches

A*11:01

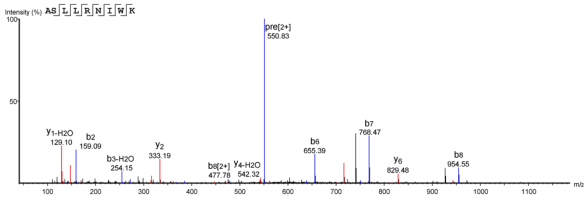

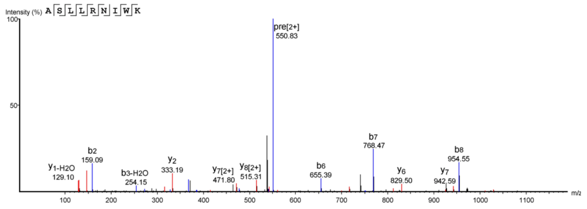

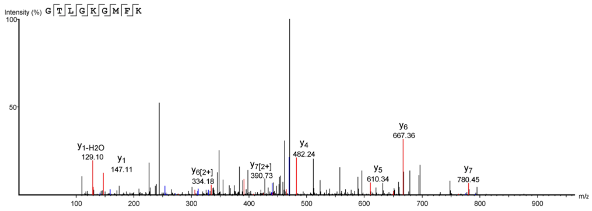

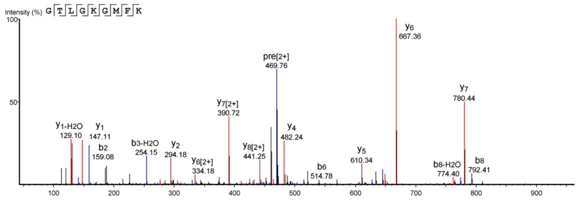

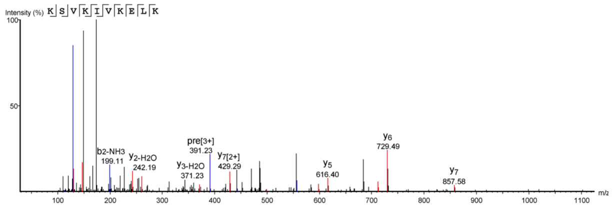

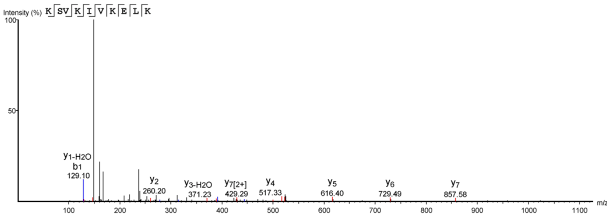

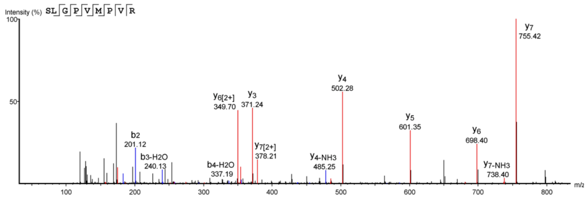

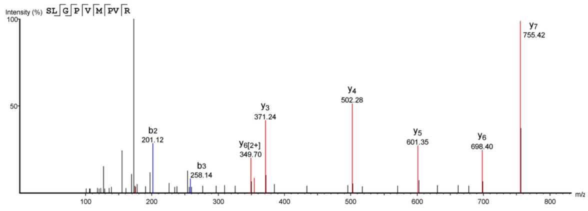

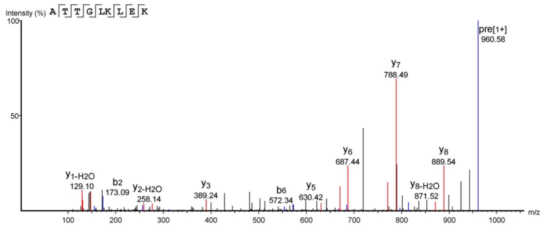

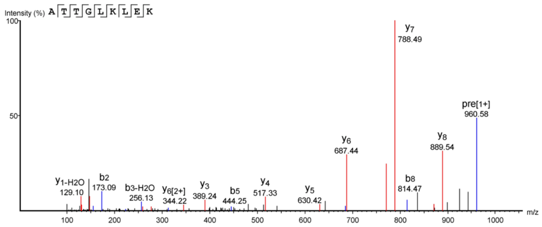

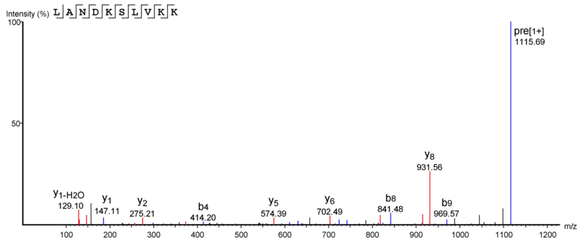

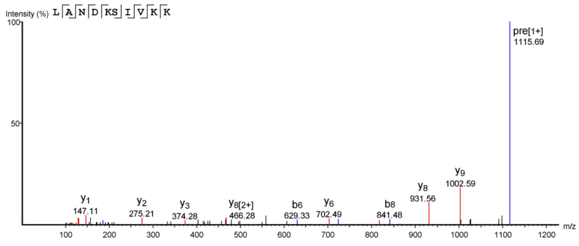

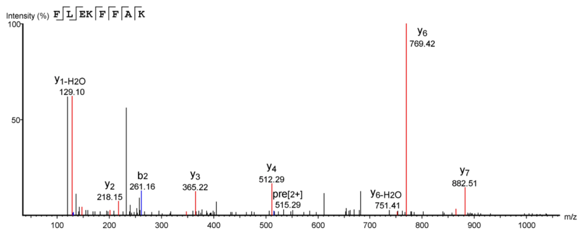

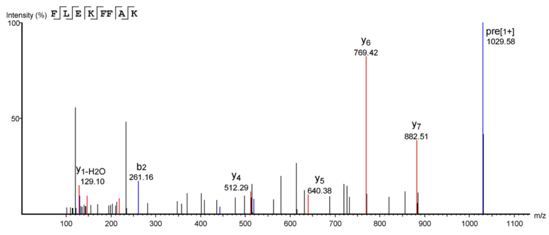

B*57:03

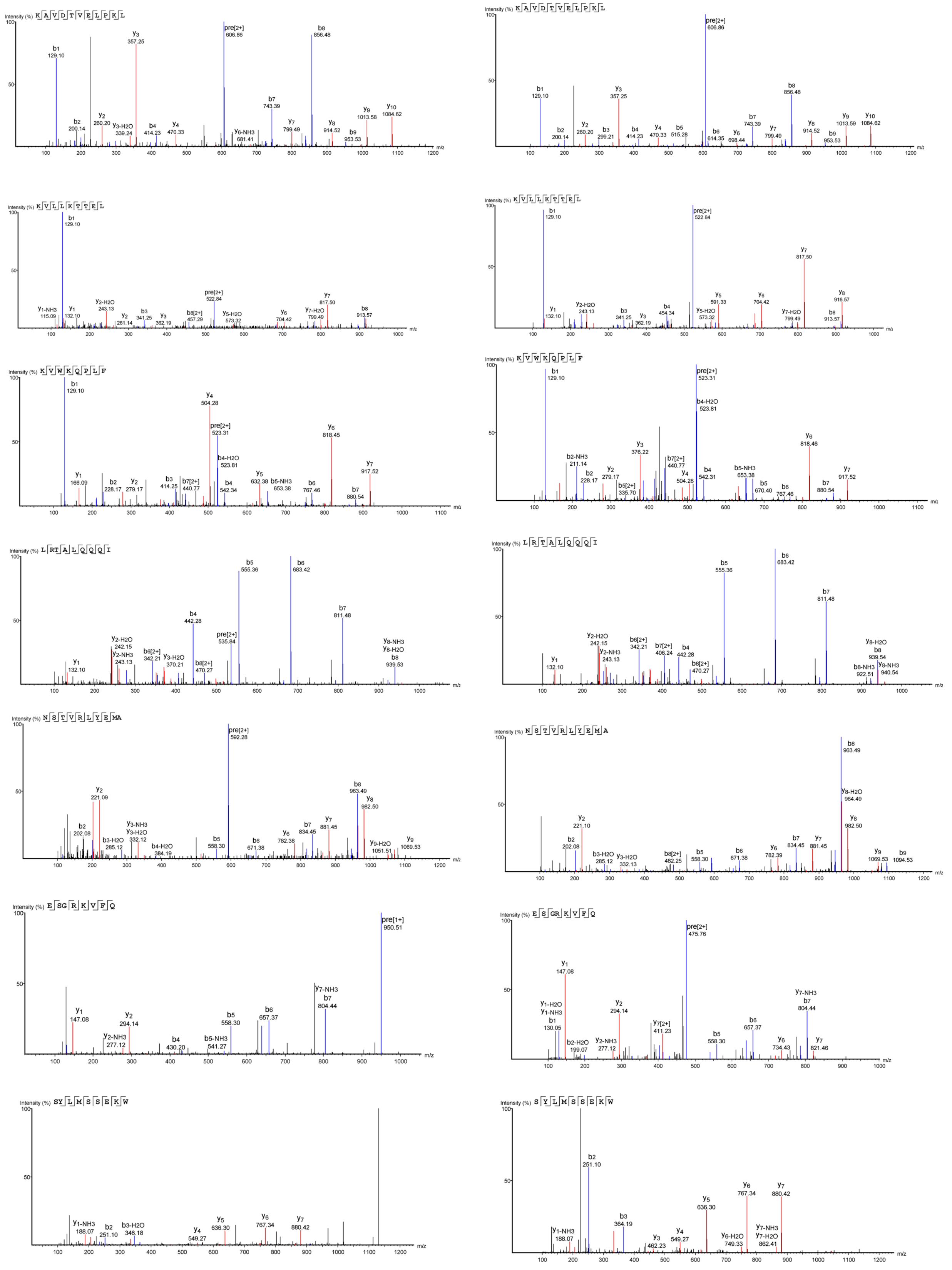
Intensity (%) KAVDTVELPKL
Intensity (%) KAVDTVELPKL
Intensity (%) KVLLKTTEL
Intensity (%) KVLLKTTEL
Intensity (%) KVWKQPLF
Intensity (%) KVWKQPLF
Intensity (%) LRTALQQQI
Intensity (%) LRTALQQQI
Intensity (%) NSTVRLYEMA
Intensity (%) NSTVRLYEMA
Intensity (%) ESGRKVFQ
Intensity (%) ESGRKVFQ
Intensity (%) SYLMSSEKW
Intensity (%) SYLMSSEKW
m/z

C*03:03

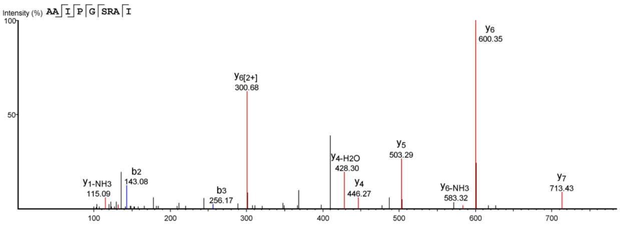

Intensity (%)
AAIPGSRAI
y6 600.35
y6[2+] 300.68
y5 503.29
y4-H2O 428.30
y4 446.27
y1-NH3 115.09
b2 143.08
b3 256.17
y6-NH3 583.32
y7 713.43

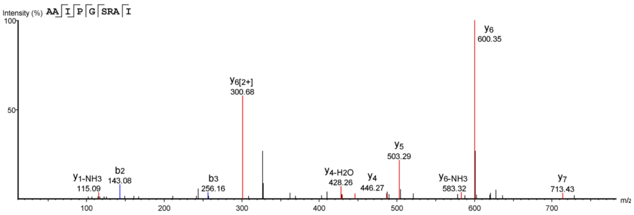

Intensity (%)
AAIPGSRAI
y6 600.35
y6[2+] 300.68
y5 503.29
y4-H2O 428.26
y4 446.27
y1-NH3 115.09
b2 143.08
b3 256.16
y6-NH3 583.32
y7 713.43
m/z

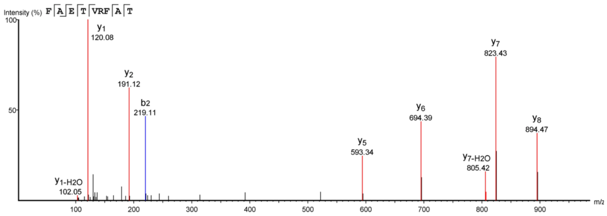

Intensity (%)
FAETVRFAT
y1 120.08
y2 191.12
b2 219.11
y1-H2O 102.05
y5 593.34
y6 694.39
y7 823.43
y7-H2O 805.42
y8 894.47
m/z

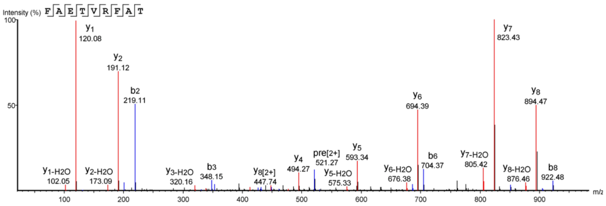

Intensity (%)
FAETVRFAT
y1 120.08
y2 191.12
b2 219.11
y7 823.43
y6 694.39
y8 894.47
y1-H2O 102.05
y2-H2O 173.09
y3-H2O 320.16
b3 348.15
y8[2+] 447.74
y4 494.27
pre[2+] 521.27
y5 593.34
y5-H2O 575.33
y6-H2O 676.38
b6 704.37
y7-H2O 805.42
y8-H2O 876.46
b8 922.48
m/z

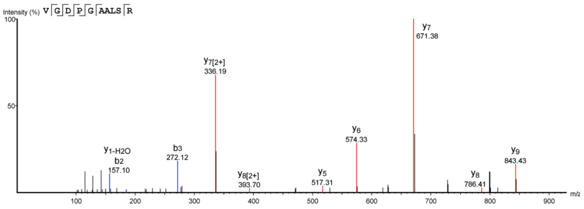

Intensity (%)
VGDPGAALSR
y7 671.38
y7[2+] 336.19
y1-H2O
b2 157.10
b3 272.12
y8[2+] 393.70
y5 517.31
y6 574.33
y8 786.41
y9 843.43
m/z

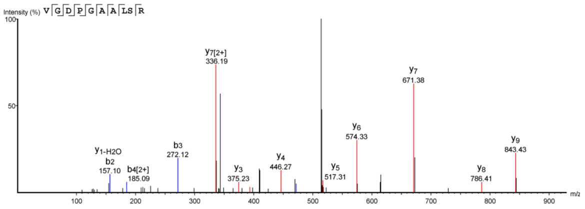

Intensity (%)
VGDPGAALSR
y7[2+] 336.19
y7 671.38
y1-H2O
b2 157.10
b4[2+] 185.09
b3 272.12
y3 375.23
y4 446.27
y5 517.31
y6 574.33
y8 786.41
y9 843.43
m/z

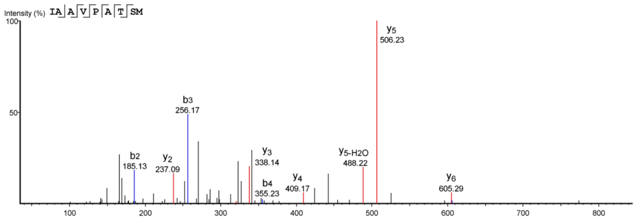

Intensity (%)
IAAVPATSM
y5 506.23
b3 256.17
b2 185.13
y2 237.09
y3 338.14
b4 355.23
y4 409.17
y5-H2O 488.22
y6 605.29

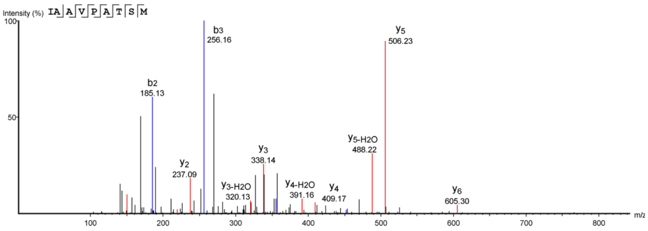

Intensity (%)
IAAVPATSM
b3 256.16
y5 506.23
b2 185.13
y2 237.09
y3-H2O 320.13
y3 338.14
y4-H2O 391.16
y4 409.17
y5-H2O 488.22
y6 605.30
m/z

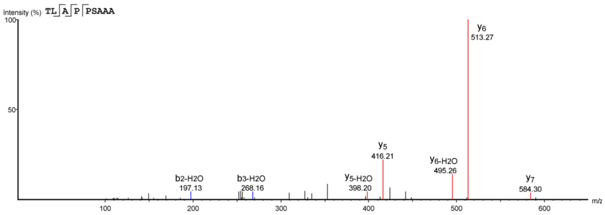

Intensity (%)
TLAPPSAAA
y6 513.27
b2-H2O 197.13
b3-H2O 268.16
y5-H2O 398.20
y5 416.21
y6-H2O 495.26
y7 584.30
m/z

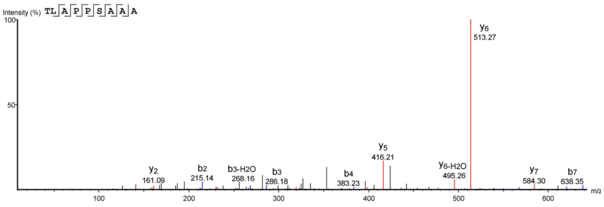

Intensity (%)
TLAPPSAAA
y6 513.27
y2 161.09
b2 215.14
b3-H2O 268.16
b3 286.18
b4 383.23
y5 416.21
y6-H2O 495.26
y7 584.30
b7 638.35
m/z

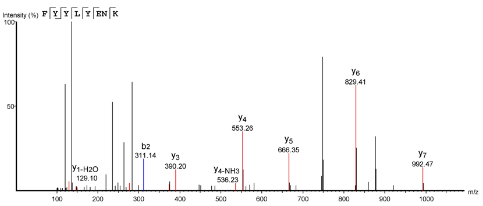

Intensity (%)
FYYLYENK
y6 829.41
y1-H2O 129.10
b2 311.14
y3 390.20
y4-NH3 536.23
y4 553.26
y5 666.35
y7 992.47
m/z

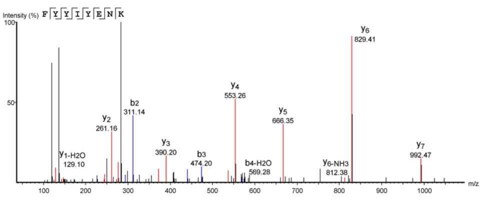

Intensity (%)
FYYIYENK
y6 829.41
y4 553.26
y5 666.35
b2 311.14
y2 261.16
y1-H2O 129.10
y3 390.20
b3 474.20
b4-H2O 569.28
y6-NH3 812.38
y7 992.47
m/z

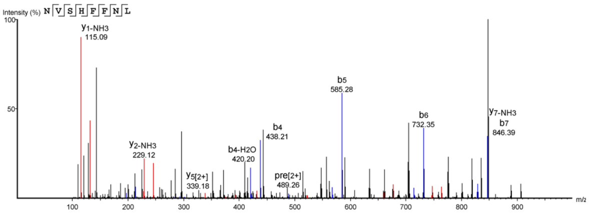

Intensity (%)
NVSHFFNL
y1-NH3 115.09
b5 585.28
b6 732.35
y7-NH3
b7 846.39
y2-NH3 229.12
b4-H2O 420.20
b4 438.21
y5[2+] 339.18
pre[2+] 489.26
m/z

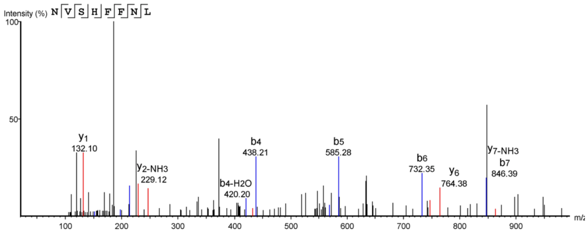

Intensity (%)
NVSHFFNL
y1 132.10
y2-NH3 229.12
b4 438.21
b4-H2O 420.20
b5 585.28
b6 732.35
y6 764.38
y7-NH3
b7 846.39
m/z

C8166

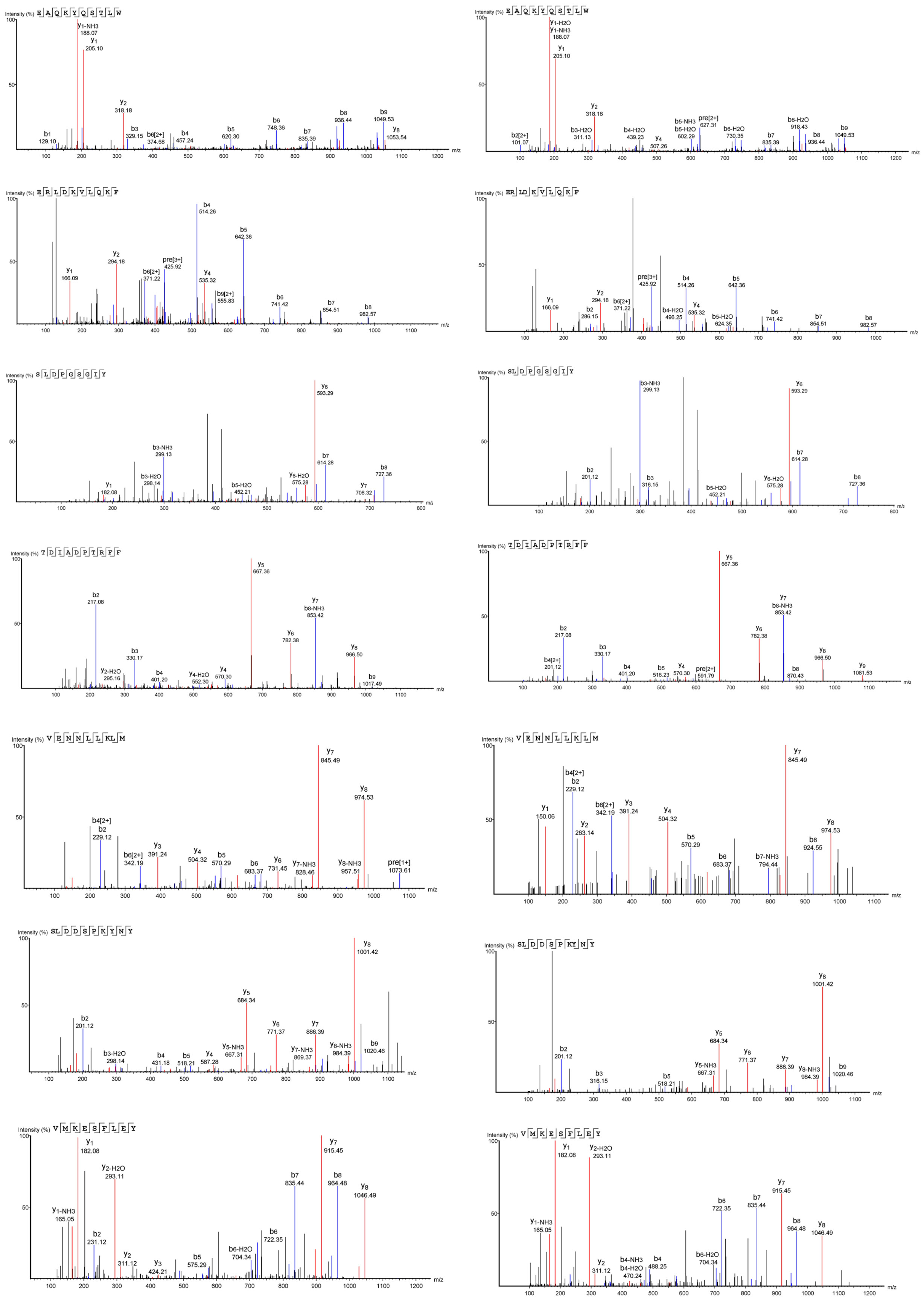

EAQKYQSTLW
EAQKYQSTLW
ERLDKVLQKF
ERLDKVLQKF
SLDPGSGIY
SLDPGSGIY
TDIADPTRFF
TDIADPTRFF
VENNLLKLM
VENNLLKLM
SLDDSPKYNY
SLDDSPKYNY
VMKESFLEY
VMKESFLEY

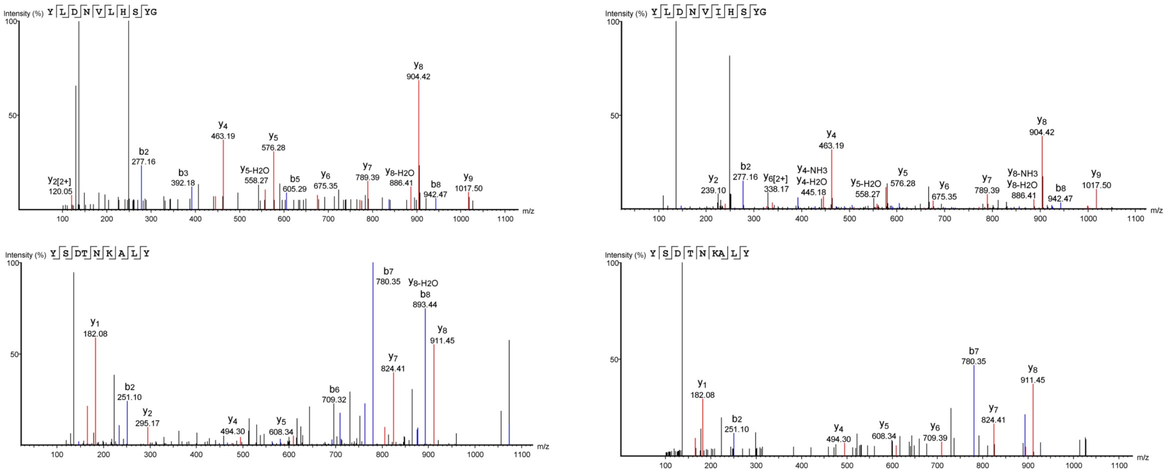

Intensity (%) YLDNVLHSYG
y2[2+] 120.05
b2 277.16
b3 392.18
y4 463.19
y5-H2O 558.27
y5 576.28
b5 605.29
y6 675.35
y7 789.39
y8-H2O 886.41
y8 904.42
b8 942.47
y9 1017.50
m/z
Intensity (%) YLDNVIHSYG
y2 239.10
b2 277.16
y6[2+] 338.17
y4-NH3
y4-H2O 445.18
y4 463.19
y5-H2O 558.27
y5 576.28
y6 675.35
y7 789.39
y8-NH3
y8-H2O 886.41
y8 904.42
b8 942.47
y9 1017.50
m/z
Intensity (%) YSDTNKALY
y1 182.08
b2 251.10
y2 295.17
y4 494.30
y5 608.34
b6 709.32
b7 780.35
y7 824.41
y8-H2O
b8 893.44
y8 911.45
m/z
Intensity (%) YSDTNKALY
y1 182.08
b2 251.10
y4 494.30
y5 608.34
y6 709.39
b7 780.35
y7 824.41
y8 911.45
m/z

ALC 50%-79% sDNUPs – non-matched

A*11:01

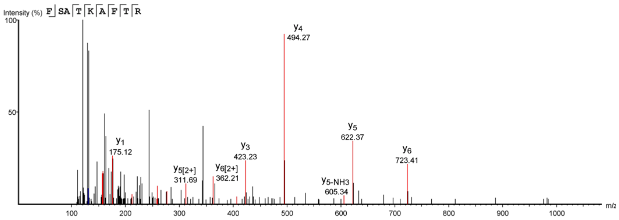

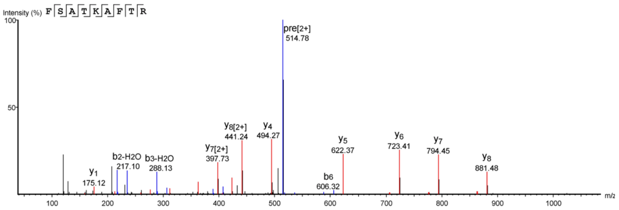

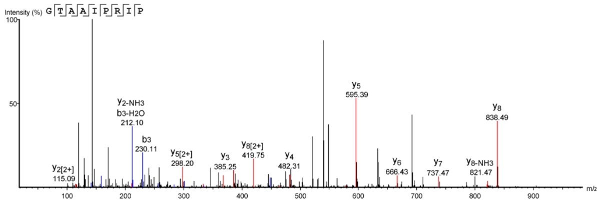

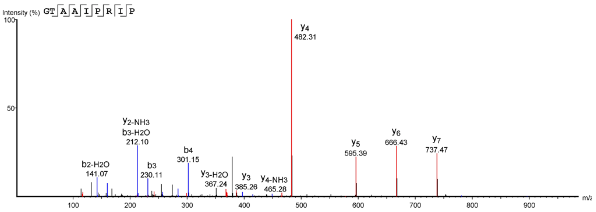

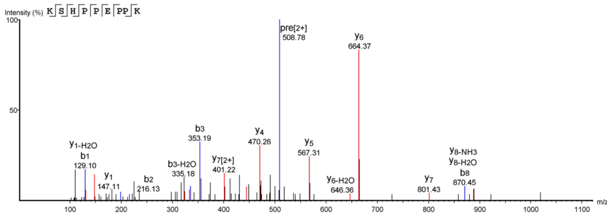

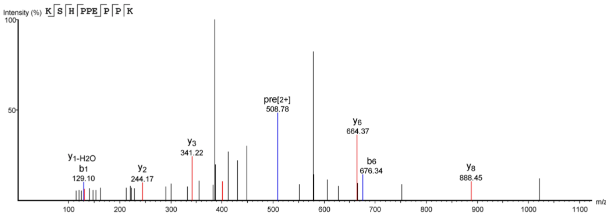

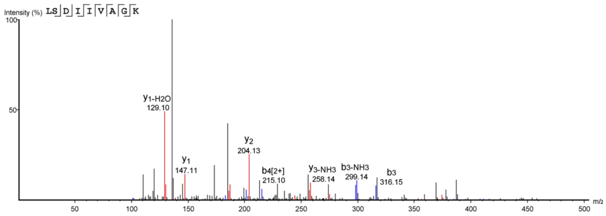

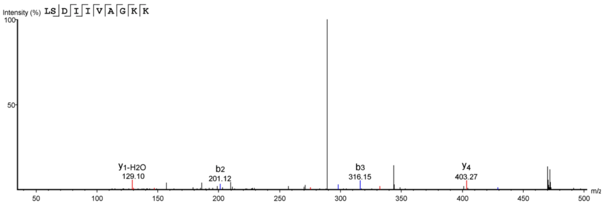

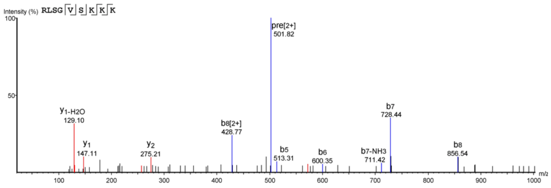

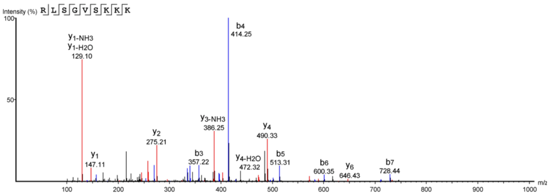

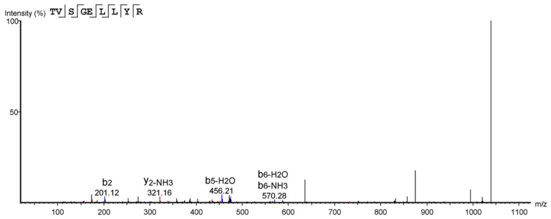

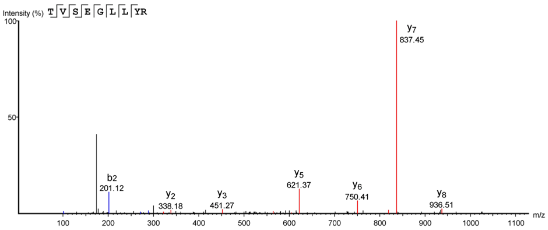

B*57:03

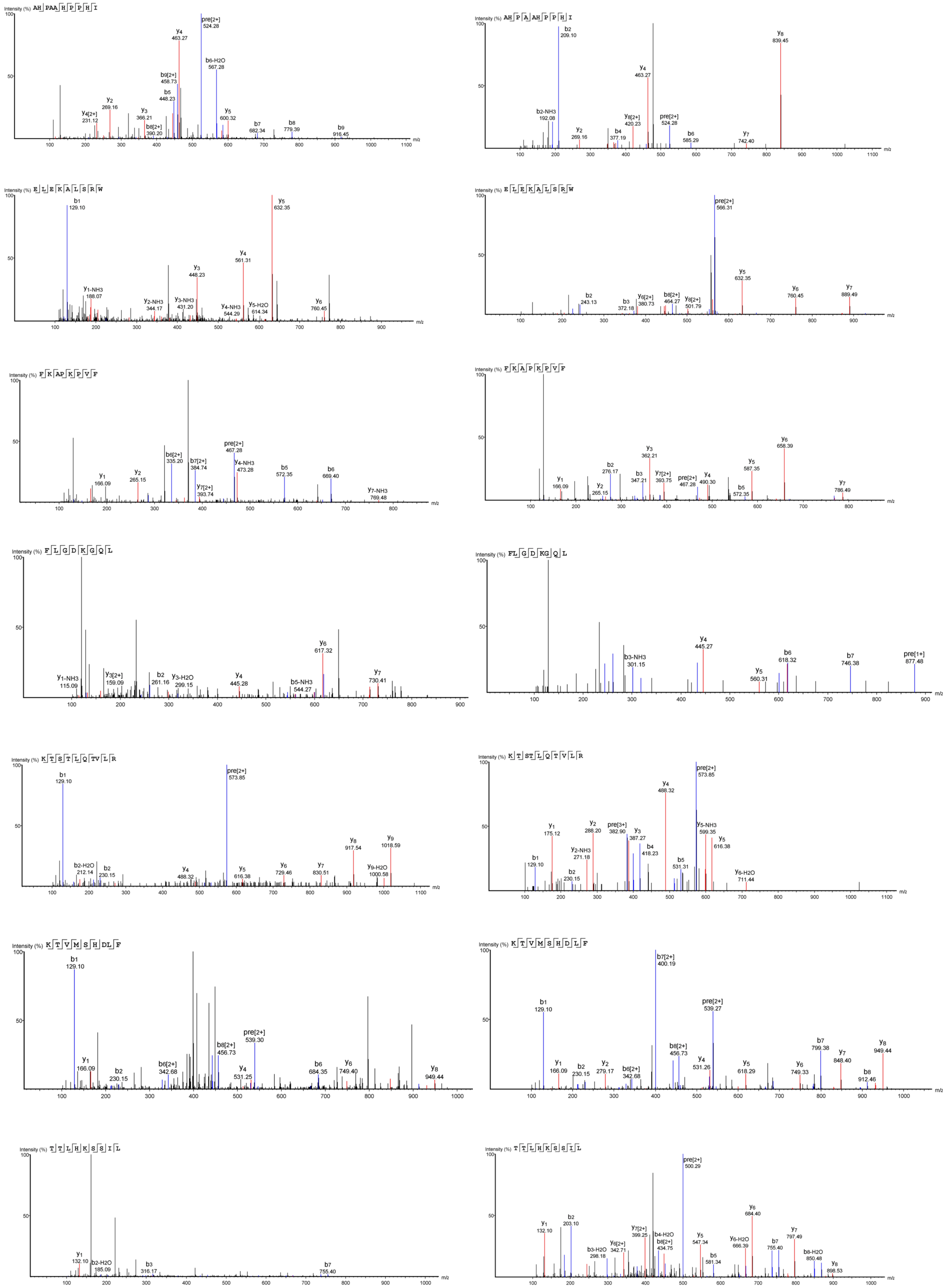
Intensity (%) AHPAAHPPHI
y4 463.27
pre[2+] 524.28
b6-H2O 567.28
b9[2+] 458.73
b5 448.23
y2 269.16
y3 366.21
y4[2+] 231.12
b8[2+] 390.20
y5 600.32
b7 682.34
b8 779.39
b9 916.45
Intensity (%) AHPAAHPPHI
b2 209.10
y8 839.45
y4 463.27
b2-NH3 192.08
y8[2+] 420.23
pre[2+] 524.28
y2 269.16
b4 377.19
b6 585.29
y7 742.40
Intensity (%) ELEKALSRW
b1 129.10
y5 632.35
y4 561.31
y3 448.23
y1-NH3 188.07
y2-NH3 344.17
y3-NH3 431.20
y4-NH3 544.29
y5-H2O 614.34
y6 760.45
Intensity (%) ELEKALSRW
pre[2+] 566.31
y5 632.35
y6 760.45
y7 889.49
b2 243.13
b3 372.18
y6[2+] 380.73
b8[2+] 464.27
y8[2+] 501.79
Intensity (%) FKAPKPVF
pre[2+] 467.28
b6[2+] 335.20
b7[2+] 384.74
y4-NH3 473.28
y1 166.09
y2 265.15
y7[2+] 393.74
b5 572.35
b6 669.40
y7-NH3 769.48
Intensity (%) FKAPKPVF
y6 658.39
y3 362.21
b2 276.17
y5 587.35
y1 166.09
b3 347.21
y7[2+] 393.75
pre[2+] 467.28
y4 490.30
y2 265.15
b5 572.35
y7 786.49
Intensity (%) FLGDKGQL
y6 617.32
y7 730.41
y1-NH3 115.09
y3[2+] 159.09
b2 261.16
y3-H2O 299.15
y4 445.28
b5-NH3 544.27
Intensity (%) FLGDKGQL
y4 445.27
b3-NH3 301.15
b6 618.32
b7 746.38
pre[1+] 877.48
y5 560.31
Intensity (%) KTSTLQTVLR
b1 129.10
pre[2+] 573.85
y8 917.54
y9 1018.59
b2-H2O 212.14
b2 230.15
y4 488.32
y5 616.38
y6 729.46
y7 830.51
y9-H2O 1000.58
Intensity (%) KTSTLQTVLR
pre[2+] 573.85
y4 488.32
y1 175.12
y2 288.20
pre[3+] 382.90
y3 387.27
y5-NH3 599.35
y5 616.38
y2-NH3 271.18
b4 418.23
b1 129.10
b2 230.15
b5 531.31
y6-H2O 711.44
Intensity (%) KTVMSHDLF
b1 129.10
pre[2+] 539.30
b8[2+] 456.73
y1 166.09
b2 230.15
b6[2+] 342.68
y4 531.25
b6 684.35
y6 749.40
y8 949.44
Intensity (%) KTVMSHDLF
b7[2+] 400.19
pre[2+] 539.27
b1 129.10
b8[2+] 456.73
y4 531.26
b7 799.38
y7 848.40
y8 949.44
y1 166.09
b2 230.15
y2 279.17
b6[2+] 342.68
y5 618.29
y6 749.33
b8 912.46
Intensity (%) TTLHKSSIL
y1 132.10
b2-H2O 185.09
b3 316.17
b7 755.40
Intensity (%) TTLHKSSIL
pre[2+] 500.29
y6 684.40
y1 132.10
b2 203.10
y7[2+] 399.25
b4-H2O 416.23
b8[2+] 434.75
y5 547.34
y6-H2O 666.39
y7 797.49
b3-H2O 298.18
y6[2+] 342.71
b5 581.34
b7 755.40
b8-H2O 850.48
y8 898.53
m/z

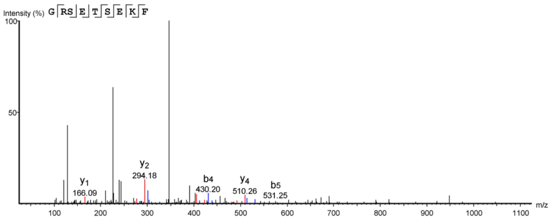
Intensity (%) G RS E T S E K F
y1 166.09
y2 294.18
b4 430.20
y4 510.26
b5 531.25
m/z

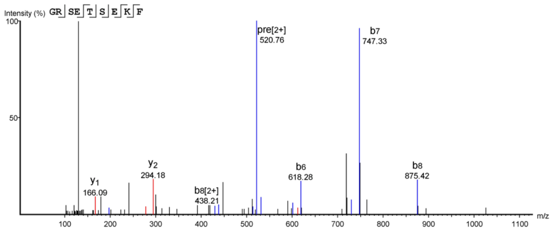
Intensity (%) GR SE T S E K F
pre[2+] 520.76
b7 747.33
y1 166.09
y2 294.18
b8[2+] 438.21
b6 618.28
b8 875.42
m/z

C*03:03

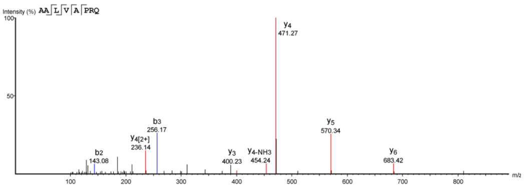
Intensity (%) AA L V A PRQ
y4 471.27
b3 256.17
y4[2+] 236.14
b2 143.08
y3 400.23
y4-NH3 454.24
y5 570.34
y6 683.42
m/z

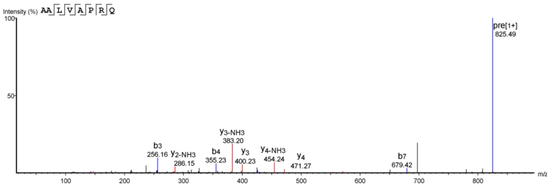
Intensity (%) AA L V A P R Q
pre[1+] 825.49
b3 256.16
y2-NH3 286.15
y3-NH3 383.20
b4 355.23
y3 400.23
y4-NH3 454.24
y4 471.27
b7 679.42
m/z

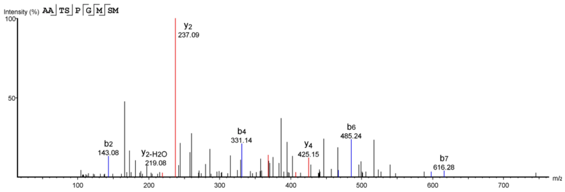
Intensity (%) AA TS P G M SM
y2 237.09
b2 143.08
y2-H2O 219.08
b4 331.14
y4 425.15
b6 485.24
b7 616.28
m/z

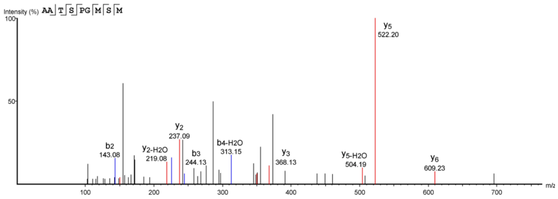
Intensity (%) AA T S PG M S M
y5 522.20
b2 143.08
y2-H2O 219.08
y2 237.09
b3 244.13
b4-H2O 313.15
y3 368.13
y5-H2O 504.19
y6 609.23
m/z

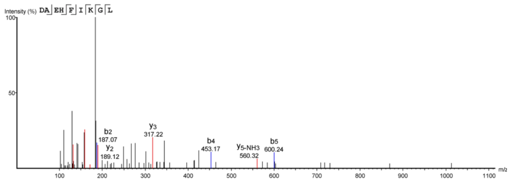
Intensity (%) DA EH F I K G L
b2 187.07
y2 189.12
y3 317.22
b4 453.17
y5-NH3 560.32
b5 600.24
m/z

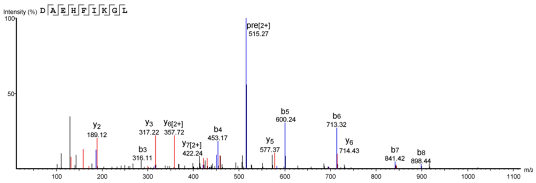
Intensity (%) D A E H F I K G L
pre[2+] 515.27
y2 189.12
y3 317.22
y6[2+] 357.72
b3 316.11
y7[2+] 422.24
b4 453.17
b5 600.24
y5 577.37
b6 713.32
y6 714.43
b7 841.42
b8 898.44
m/z

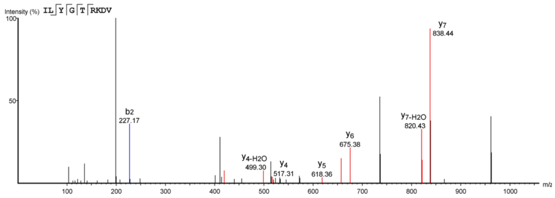
Intensity (%) IL Y G T RKDV
y7 838.44
b2 227.17
y7-H2O 820.43
y6 675.38
y4-H2O 499.30
y4 517.31
y5 618.36
m/z

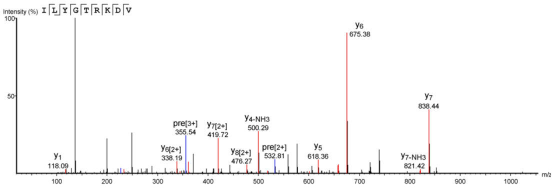
Intensity (%) I L Y G T R K D V
y6 675.38
y7 838.44
y4-NH3 500.29
pre[3+] 355.54
y7[2+] 419.72
y6[2+] 338.19
y8[2+] 476.27
pre[2+] 532.81
y5 618.36
y1 118.09
y7-NH3 821.42
m/z

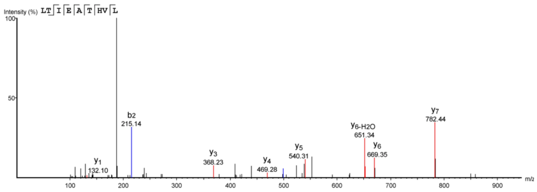
Intensity (%) LT I E A T HV L
b2 215.14
y6-H2O 651.34
y6 669.35
y7 782.44
y1 132.10
y3 368.23
y4 469.28
y5 540.31
m/z

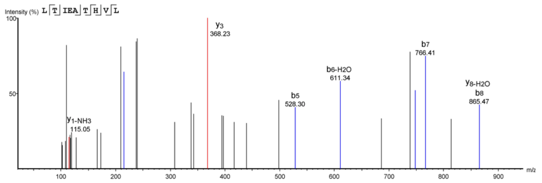
Intensity (%) L T IEA T H V L
y3 368.23
b7 766.41
b6-H2O 611.34
y8-H2O b8 865.47
b5 528.30
y1-NH3 115.05
m/z

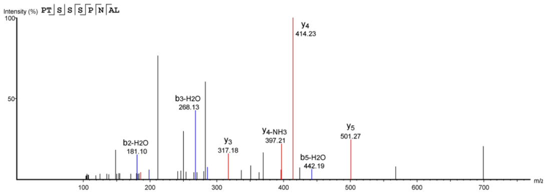
Intensity (%) PT S S P N AL
y4 414.23
b3-H2O 268.13
b2-H2O 181.10
y3 317.18
y4-NH3 397.21
b5-H2O 442.19
y5 501.27
m/z

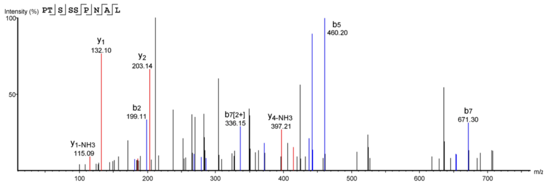
Intensity (%) PT S SS P N A L
b5 460.20
y1 132.10
y2 203.14
b2 199.11
b7[2+] 336.15
y4-NH3 397.21
y1-NH3 115.09
b7 671.30
m/z

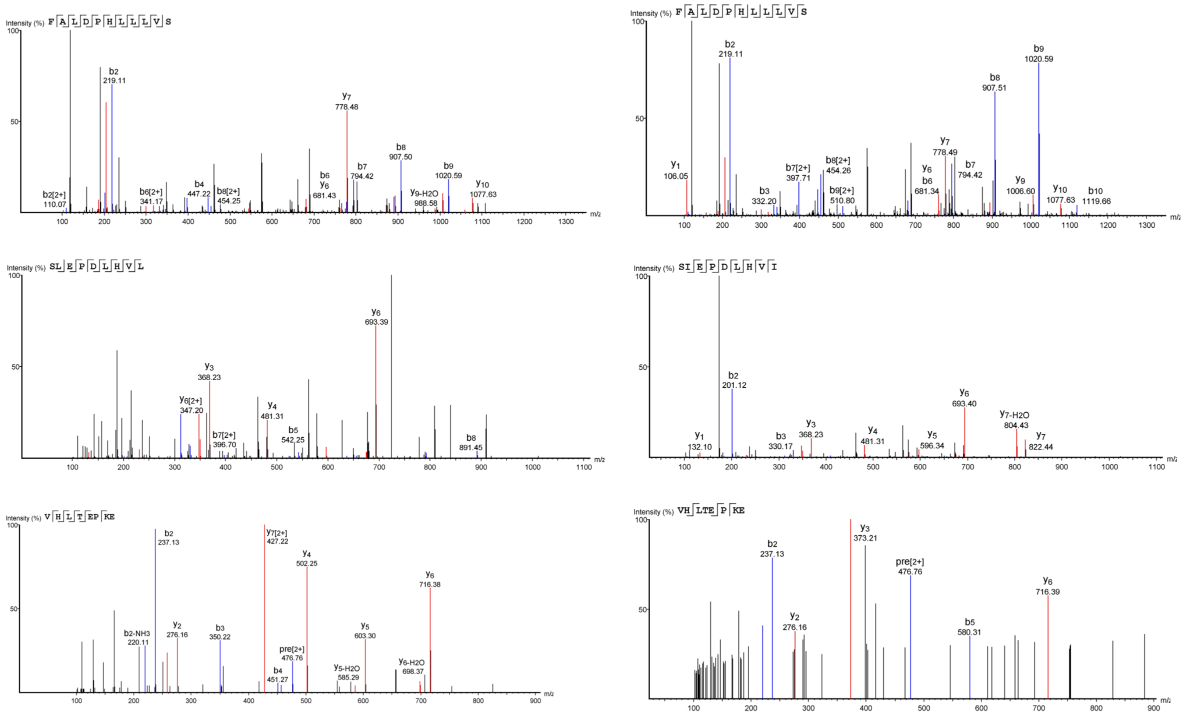
FALDPHLLLVS
FALDPHLLLVS
SLEPDLHVL
SIEPDLHVI
VHLTEPKE
VHLTEPKE

C8166

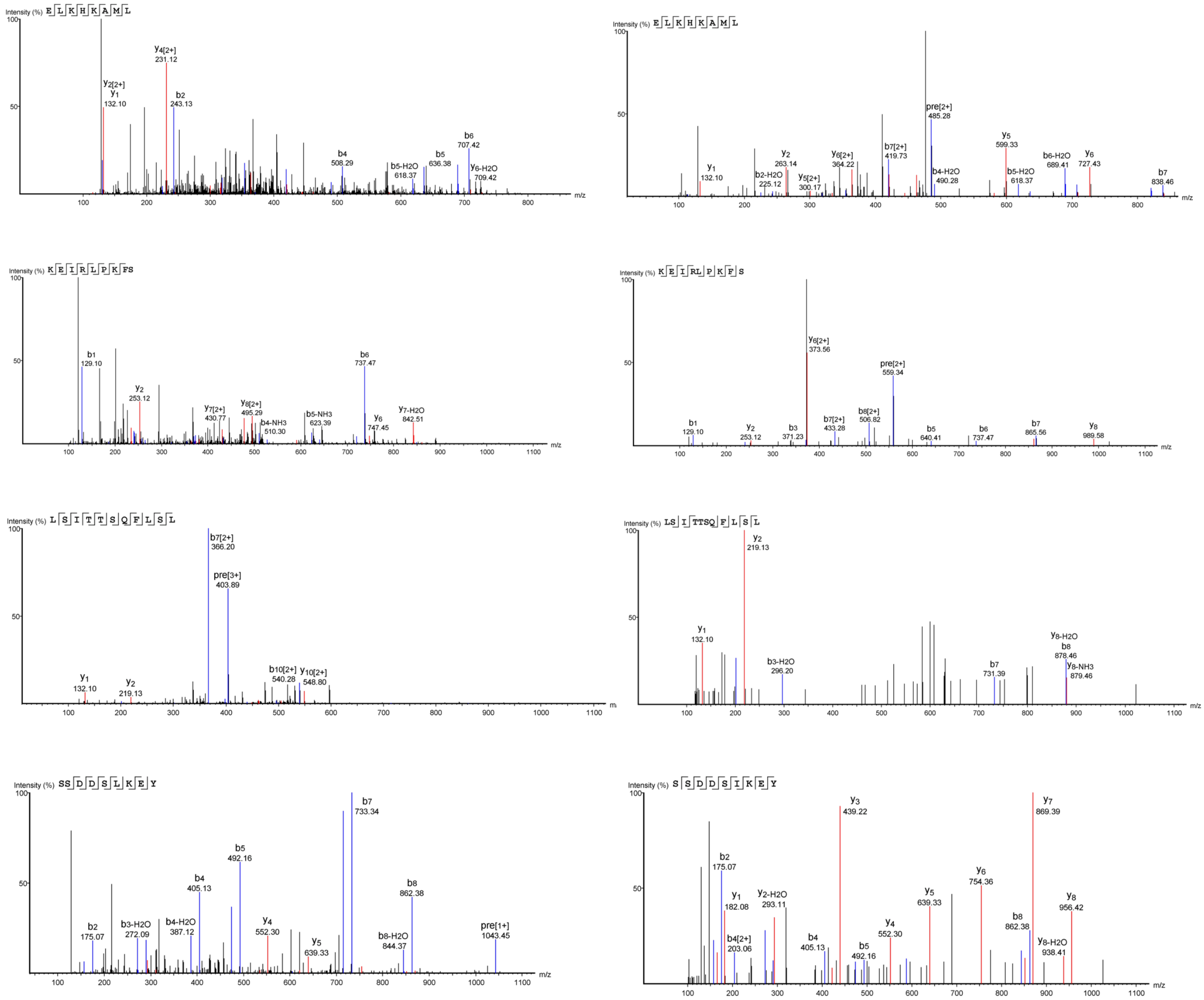
ELKHKAML
ELKHKAML
KEIRLPKFS
KEIRLPKFS
LSITTSQFLSL
LSITTSQFLSL
SSDDSLKEY
SSDDSIKEY

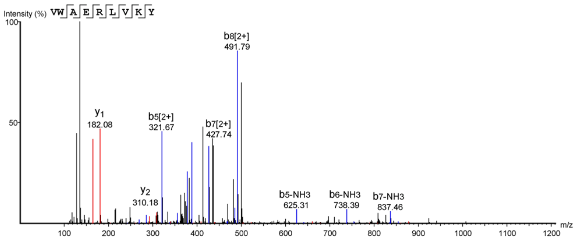
Intensity (%)
VWAERLVKY
100
50
y1
182.08
y2
310.18
b5[2+]
321.67
b7[2+]
427.74
b8[2+]
491.79
b5-NH3
625.31
b6-NH3
738.39
b7-NH3
837.46
100 200 300 400 500 600 700 800 900 1000 1100 1200
m/z

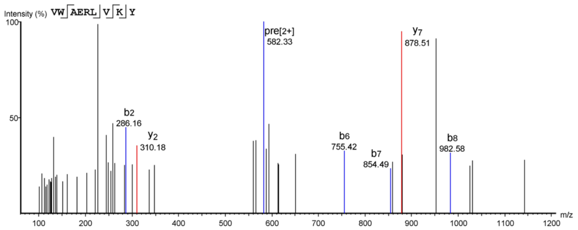
Intensity (%)
VWAERLVKY
100
50
b2
286.16
y2
310.18
pre[2+]
582.33
b6
755.42
b7
854.49
y7
878.51
b8
982.58
100 200 300 400 500 600 700 800 900 1000 1100 1200
m/z

ALC 80%-99% non-spliced – spectral matches

A*11:01

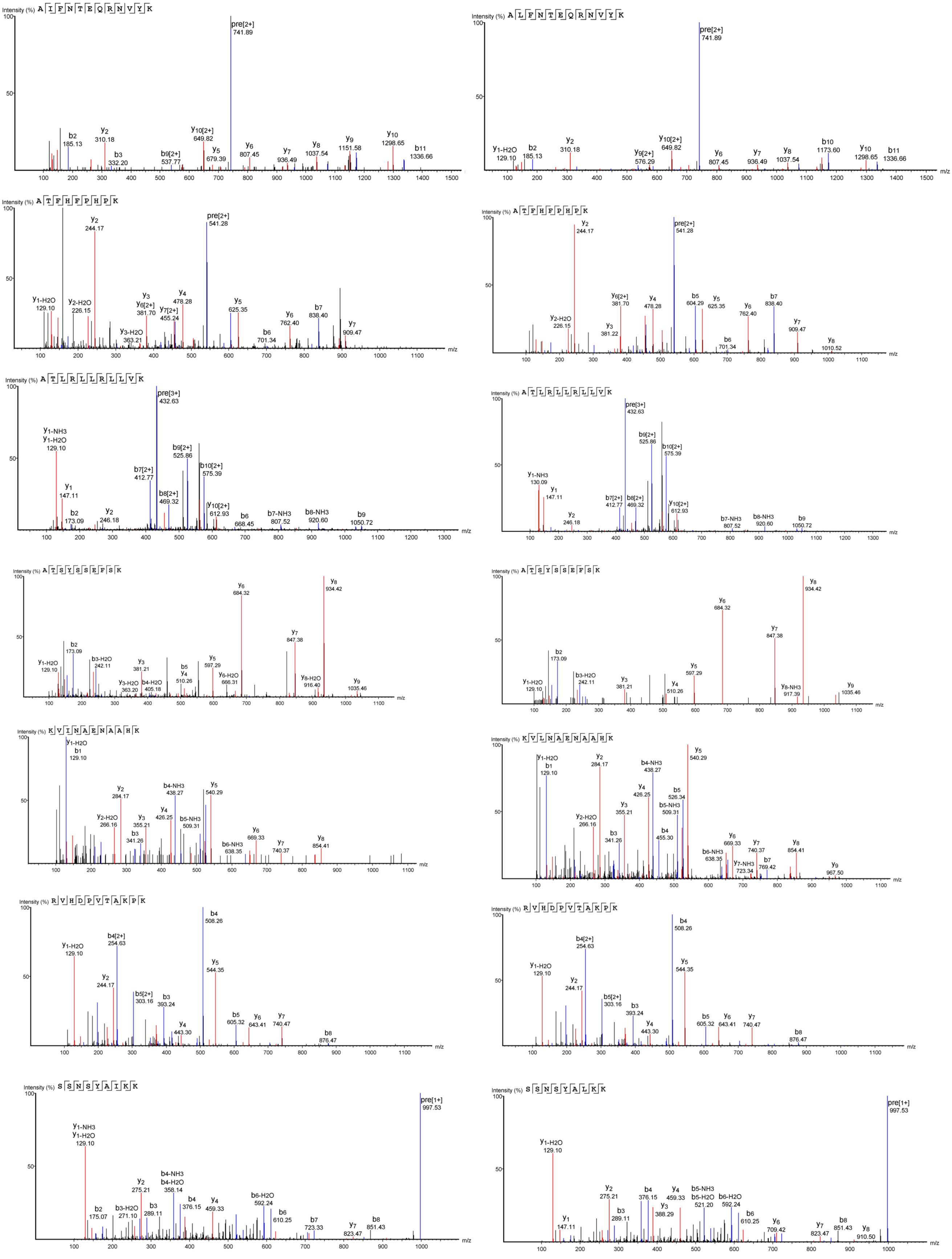

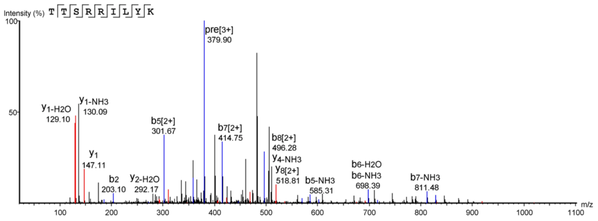
Intensity (%) T T S R R I L Y K
pre[3+] 379.90
y1-H2O 129.10
y1-NH3 130.09
y1 147.11
b2 203.10
y2-H2O 292.17
b5[2+] 301.67
b7[2+] 414.75
b8[2+] 496.28
y4-NH3
y8[2+] 518.81
b5-NH3 585.31
b6-H2O
b6-NH3 698.39
b7-NH3 811.48
m/z

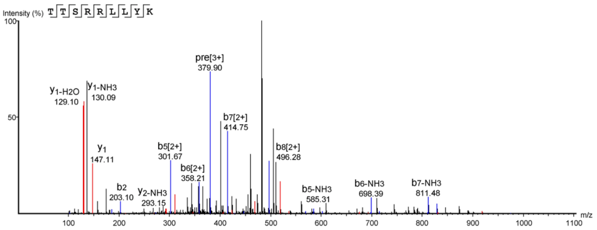
Intensity (%) T T S R R L L Y K
pre[3+] 379.90
y1-H2O 129.10
y1-NH3 130.09
y1 147.11
b2 203.10
y2-NH3 293.15
b5[2+] 301.67
b6[2+] 358.21
b7[2+] 414.75
b8[2+] 496.28
b5-NH3 585.31
b6-NH3 698.39
b7-NH3 811.48
m/z

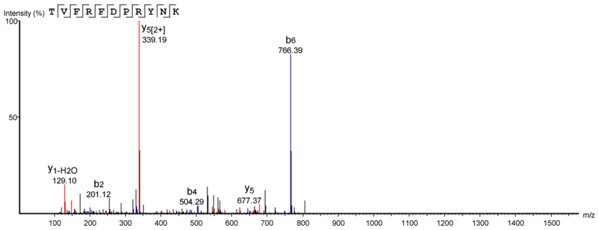
Intensity (%) T V F R F D P R Y N K
y5[2+] 339.19
b6 766.39
y1-H2O 129.10
b2 201.12
b4 504.29
y5 677.37
m/z

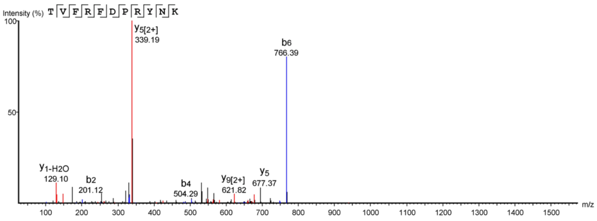
Intensity (%) T V F R F D P R Y N K
y5[2+] 339.19
b6 766.39
y1-H2O 129.10
b2 201.12
b4 504.29
y9[2+] 621.82
y5 677.37
m/z

B*57:03

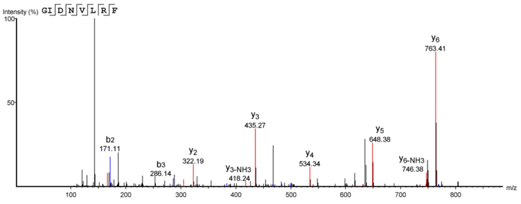
Intensity (%) GI D N V L R F
y6 763.41
y3 435.27
y5 648.38
b2 171.11
y2 322.19
y4 534.34
b3 286.14
y3-NH3 418.24
y6-NH3 746.38
m/z

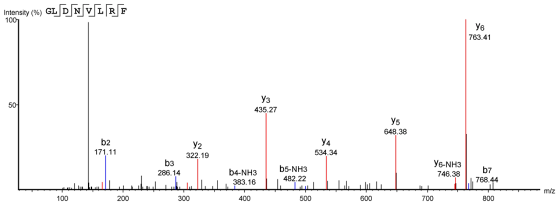
Intensity (%) GL D N V L R F
y6 763.41
y3 435.27
y5 648.38
b2 171.11
y2 322.19
y4 534.34
b3 286.14
b4-NH3 383.16
b5-NH3 482.22
y6-NH3 746.38
b7 768.44
m/z

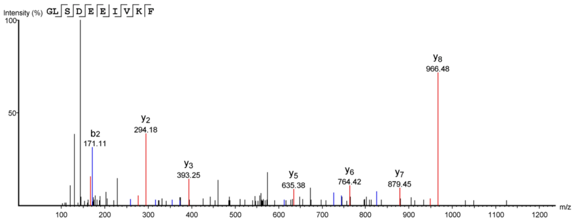
Intensity (%) GL S D E E I V K F
y8 966.48
y2 294.18
b2 171.11
y3 393.25
y5 635.38
y6 764.42
y7 879.45
m/z

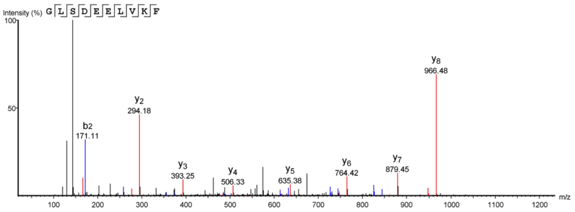
Intensity (%) G L S D E E L V K F
y8 966.48
y2 294.18
b2 171.11
y3 393.25
y4 506.33
y5 635.38
y6 764.42
y7 879.45
m/z

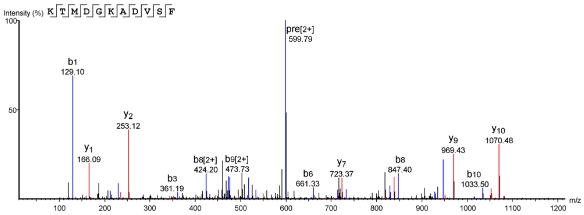
Intensity (%) K T M D G K A D V S F
pre[2+] 599.79
b1 129.10
y2 253.12
y1 166.09
b8[2+] 424.20
b9[2+] 473.73
b3 361.19
b6 661.33
y7 723.37
b8 847.40
y9 969.43
y10 1070.48
b10 1033.50
m/z

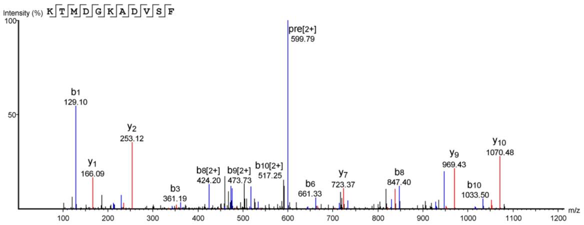
Intensity (%) K T M D G K A D V S F
pre[2+] 599.79
b1 129.10
y2 253.12
y1 166.09
b3 361.19
b8[2+] 424.20
b9[2+] 473.73
b10[2+] 517.25
b6 661.33
y7 723.37
b8 847.40
y9 969.43
y10 1070.48
b10 1033.50
m/z

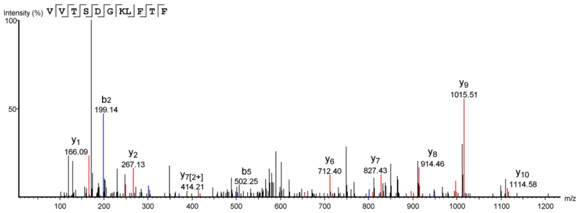
Intensity (%) V V T S D G KL F T F
b2 199.14
y9 1015.51
y1 166.09
y2 267.13
y7[2+] 414.21
b5 502.25
y6 712.40
y7 827.43
y8 914.46
y10 1114.58
m/z

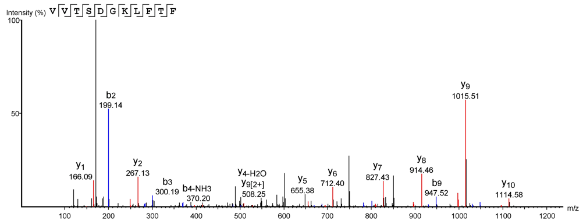
Intensity (%) V V T S D G K L F T F
b2 199.14
y9 1015.51
y1 166.09
y2 267.13
b3 300.19
b4-NH3 370.20
y4-H2O
y9[2+] 508.25
y5 655.38
y6 712.40
y7 827.43
y8 914.46
b9 947.52
y10 1114.58
m/z

C*03:03

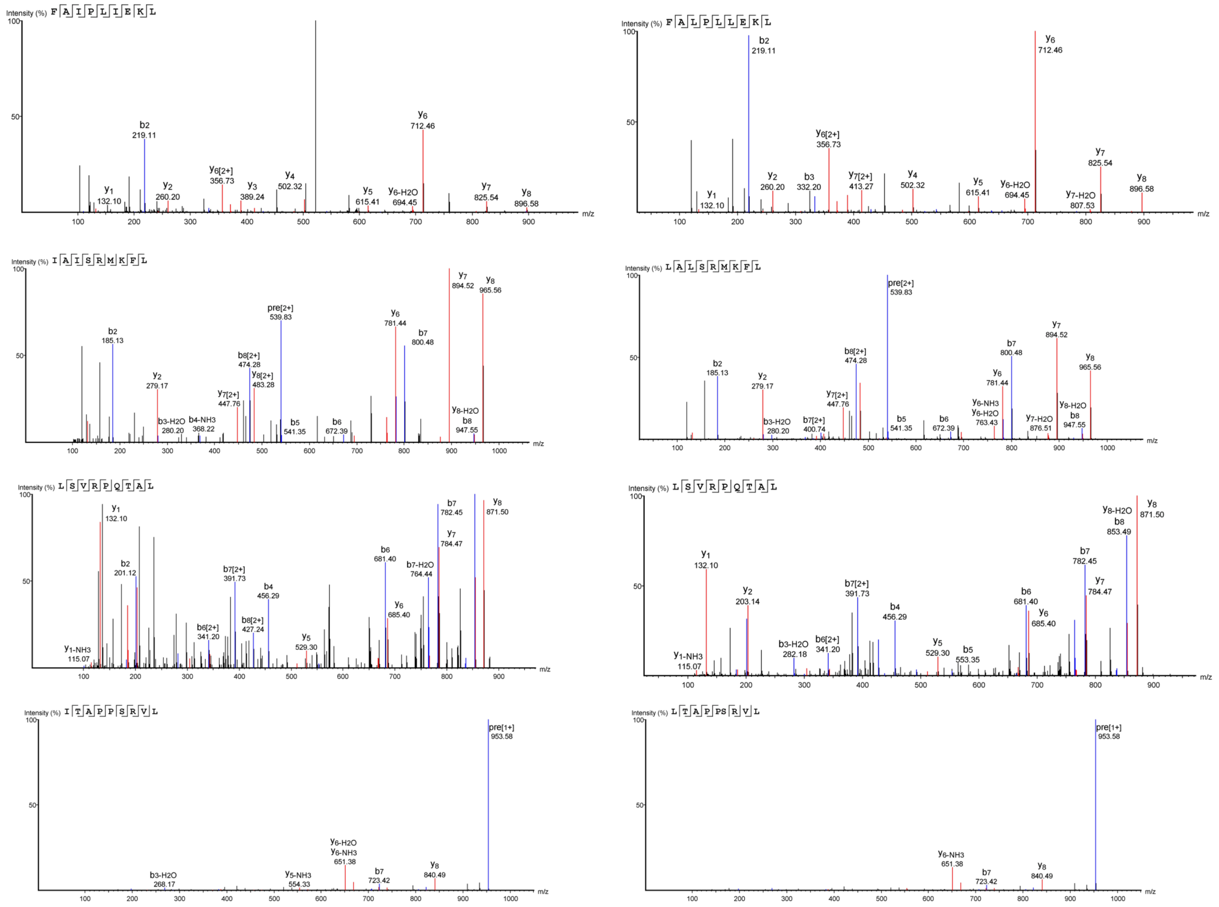
Intensity (%)
m/z
FAIPLIEKL
FALPLLEKL
IAISRMKFL
LALSRMKFL
LSVRPQTAL
LSVRPQTAL
ITAPPSRVL
LTAPPSRVL

C8166

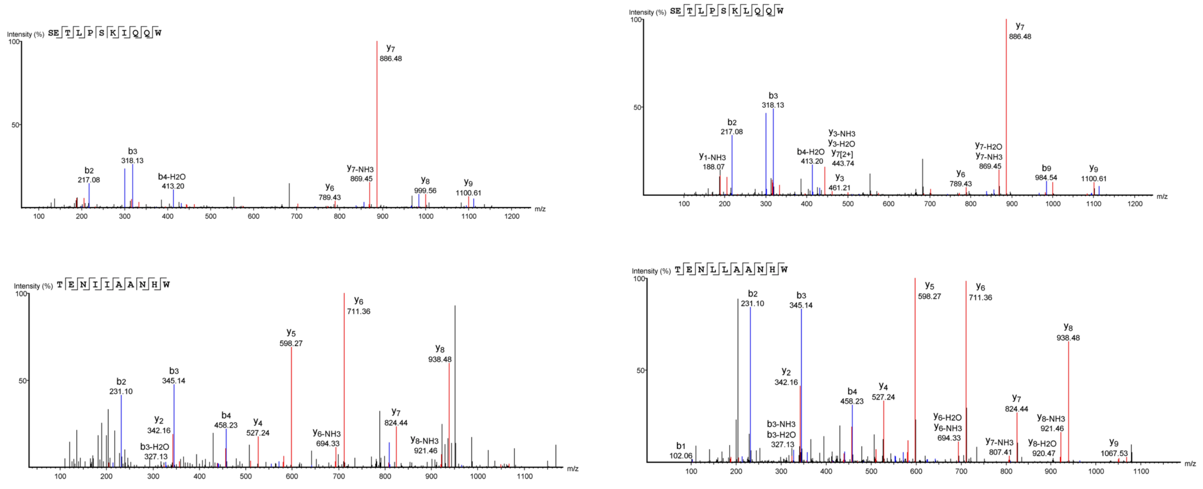
Intensity (%)
m/z
SETLPSKIQQW
SETLPSKLQQW
TENIIAANHW
TENLLAANHW

ALC 80%-99% non-spliced - non-matched

A*11:01

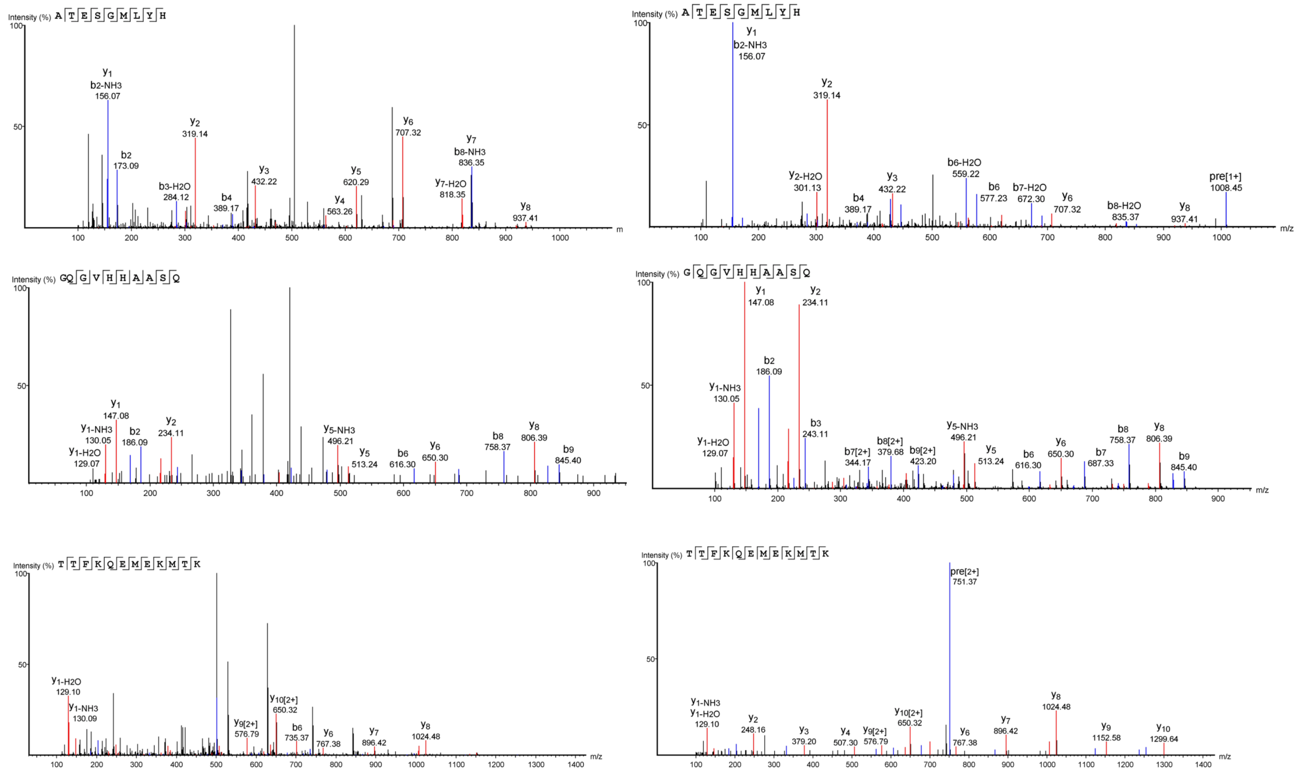

B*57:03

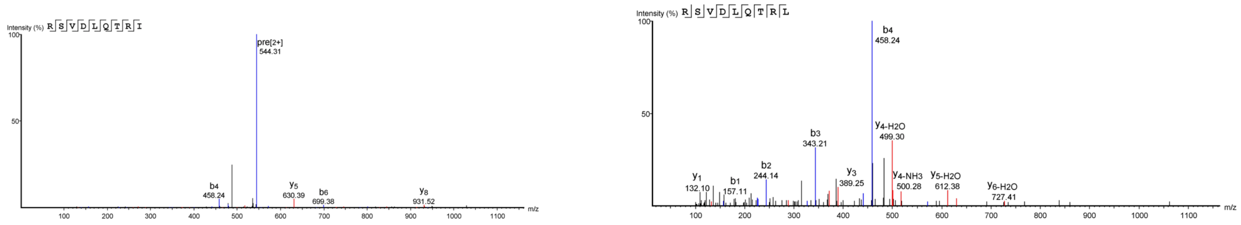

C*03:03

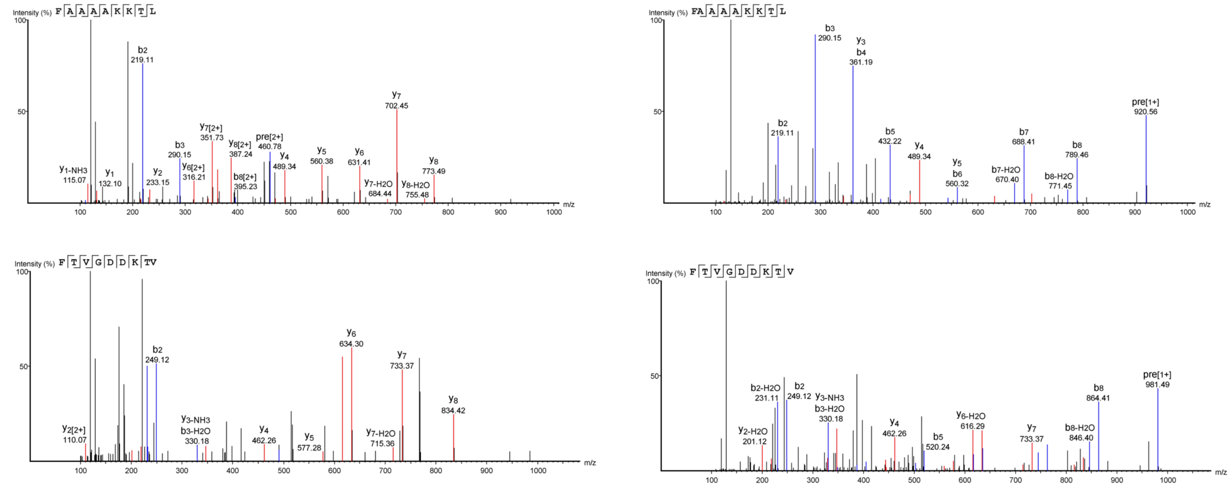

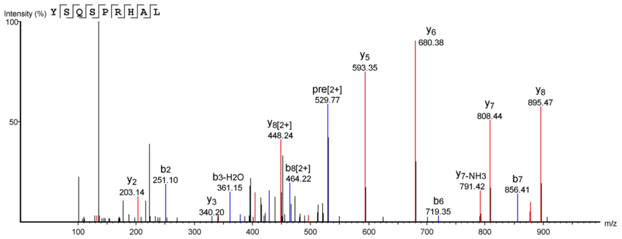

Intensity (%) YSQSPRHAL
100
50
y2 203.14
b2 251.10
y3 340.20
b3-H2O 361.15
y8[2+] 448.24
b8[2+] 464.22
pre[2+] 529.77
y5 593.35
y6 680.38
b6 719.35
y7-NH3 791.42
y7 808.44
b7 856.41
y8 895.47
100 200 300 400 500 600 700 800 900
m/z

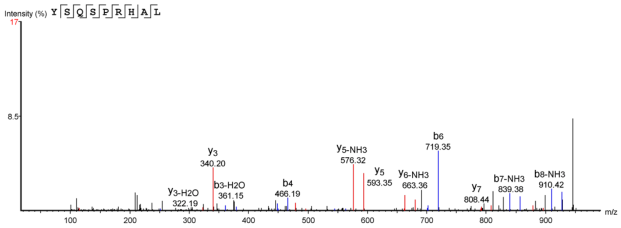

Intensity (%) YSQSPRHAL
17
8.5
y3-H2O 322.19
y3 340.20
b3-H2O 361.15
b4 466.19
y5-NH3 576.32
y5 593.35
y6-NH3 663.36
b6 719.35
y7 808.44
b7-NH3 839.38
b8-NH3 910.42
100 200 300 400 500 600 700 800 900
m/z

C8166

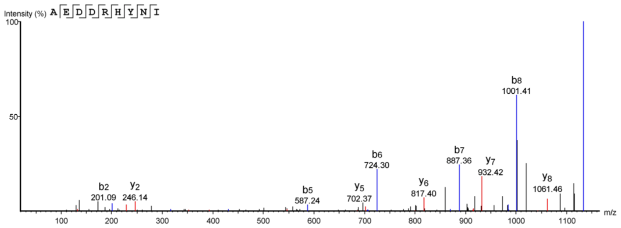

Intensity (%) AEDDRHYNI
100
50
b2 201.09
y2 246.14
b5 587.24
y5 702.37
b6 724.30
y6 817.40
b7 887.36
y7 932.42
b8 1001.41
y8 1061.46
100 200 300 400 500 600 700 800 900 1000 1100
m/z

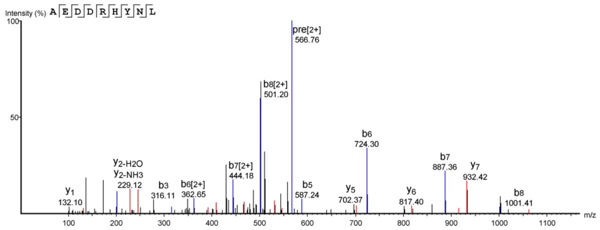

Intensity (%) AEDDRHYNL
100
50
y1 132.10
y2-H2O
y2-NH3 229.12
b3 316.11
b6[2+] 362.65
b7[2+] 444.18
b8[2+] 501.20
pre[2+] 566.76
b5 587.24
y5 702.37
b6 724.30
y6 817.40
b7 887.36
y7 932.42
b8 1001.41
100 200 300 400 500 600 700 800 900 1000 1100
m/z

ALC 50%-79% non-spliced – spectral matches

A*11:01

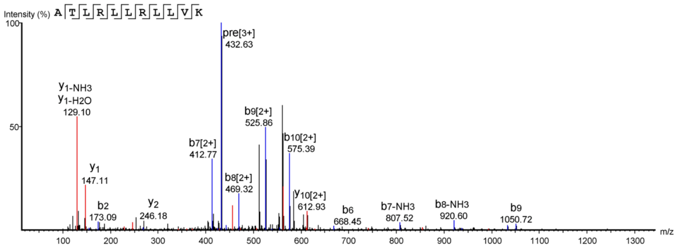

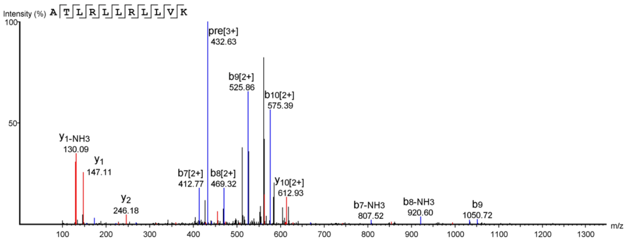

B*57:03

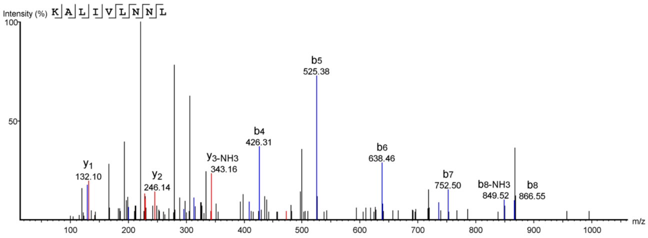

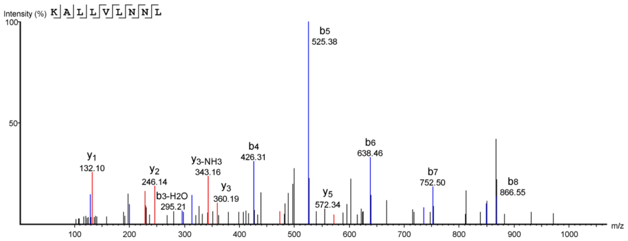

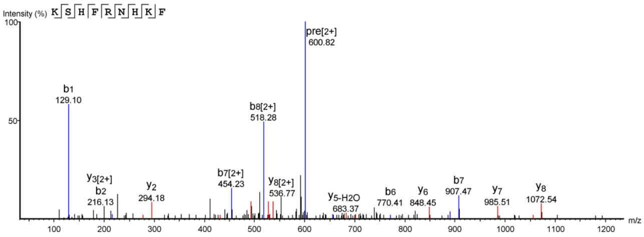

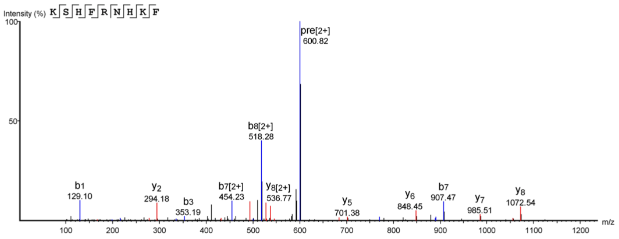

C*03:03

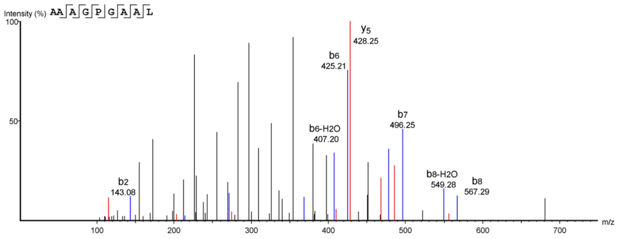

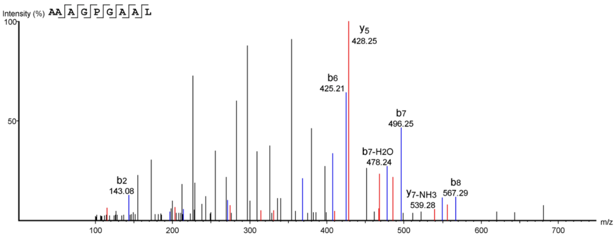

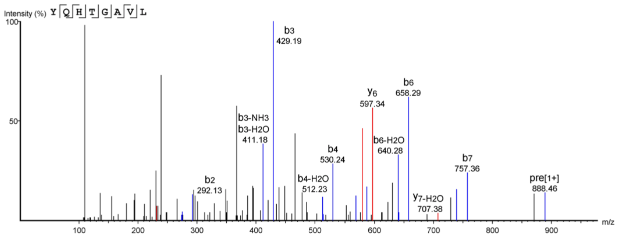

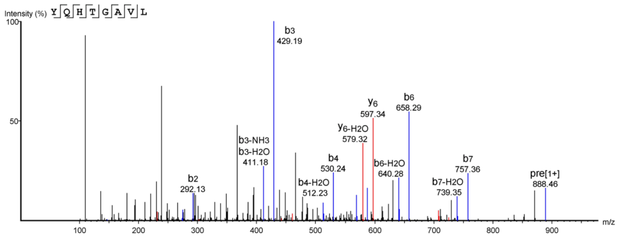

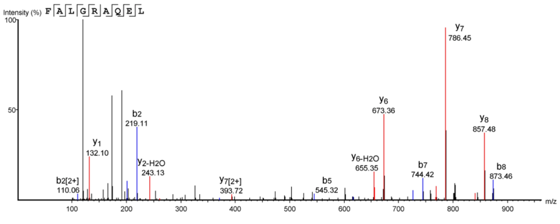

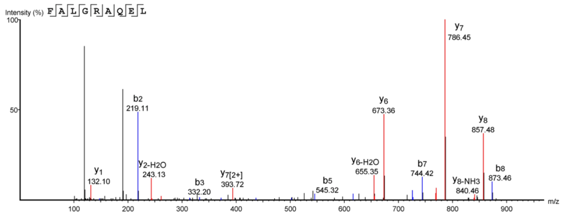

C8166

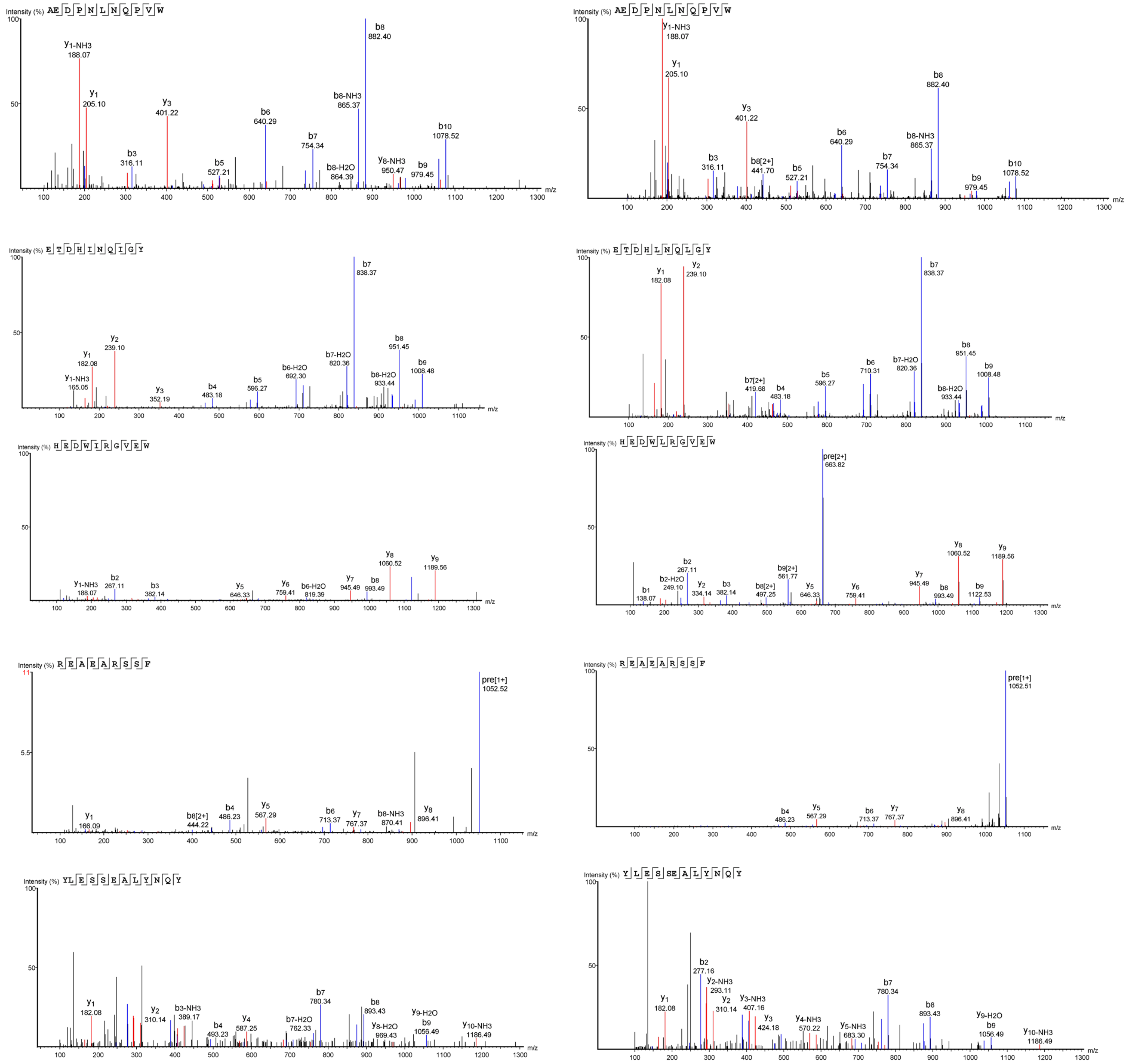
Intensity (%) AEDPNLNQPVW
Intensity (%) AEDPNLNQPVW
Intensity (%) ETDHINQIGY
Intensity (%) ETDHLNQLGY
Intensity (%) HEDWIRGVEW
Intensity (%) HEDWLRGVEW
Intensity (%) REAEARSSF
Intensity (%) REAEARSSF
Intensity (%) YLESSEALYNQY
Intensity (%) YLESSEALYNQY
m/z

ALC 50%-79% non-spliced – non-matched

A*11:01

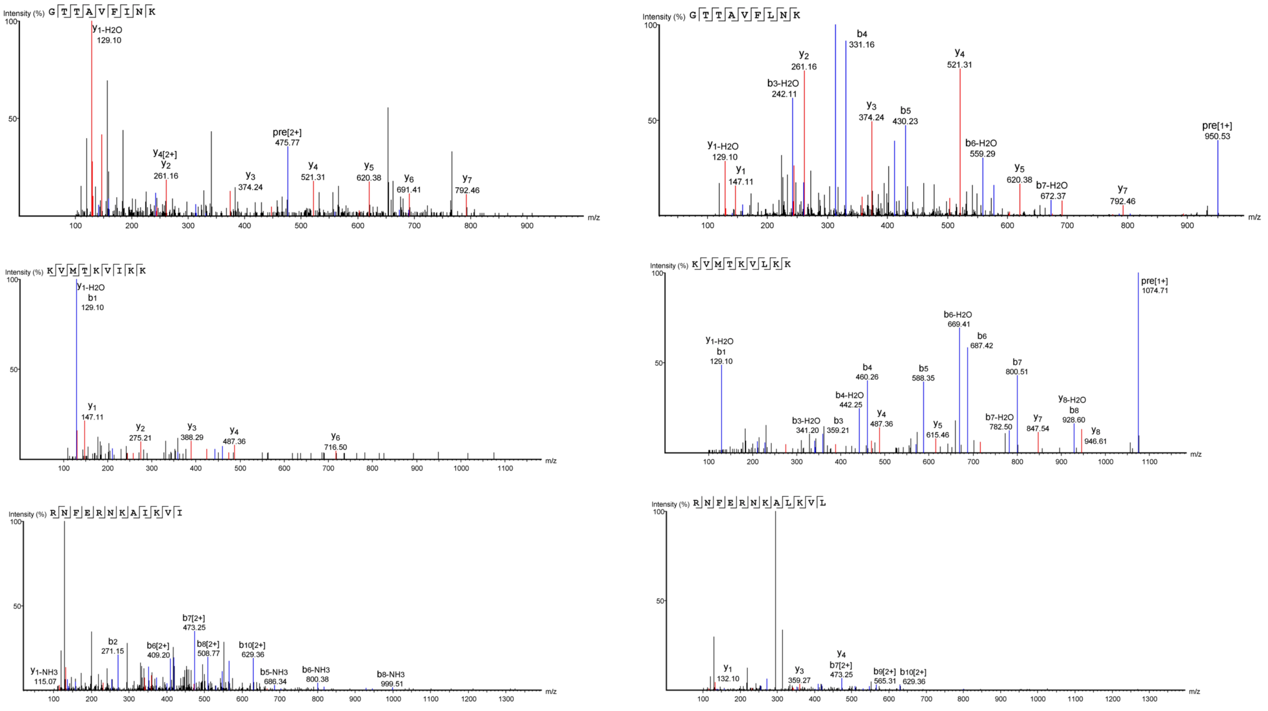

B*57:03

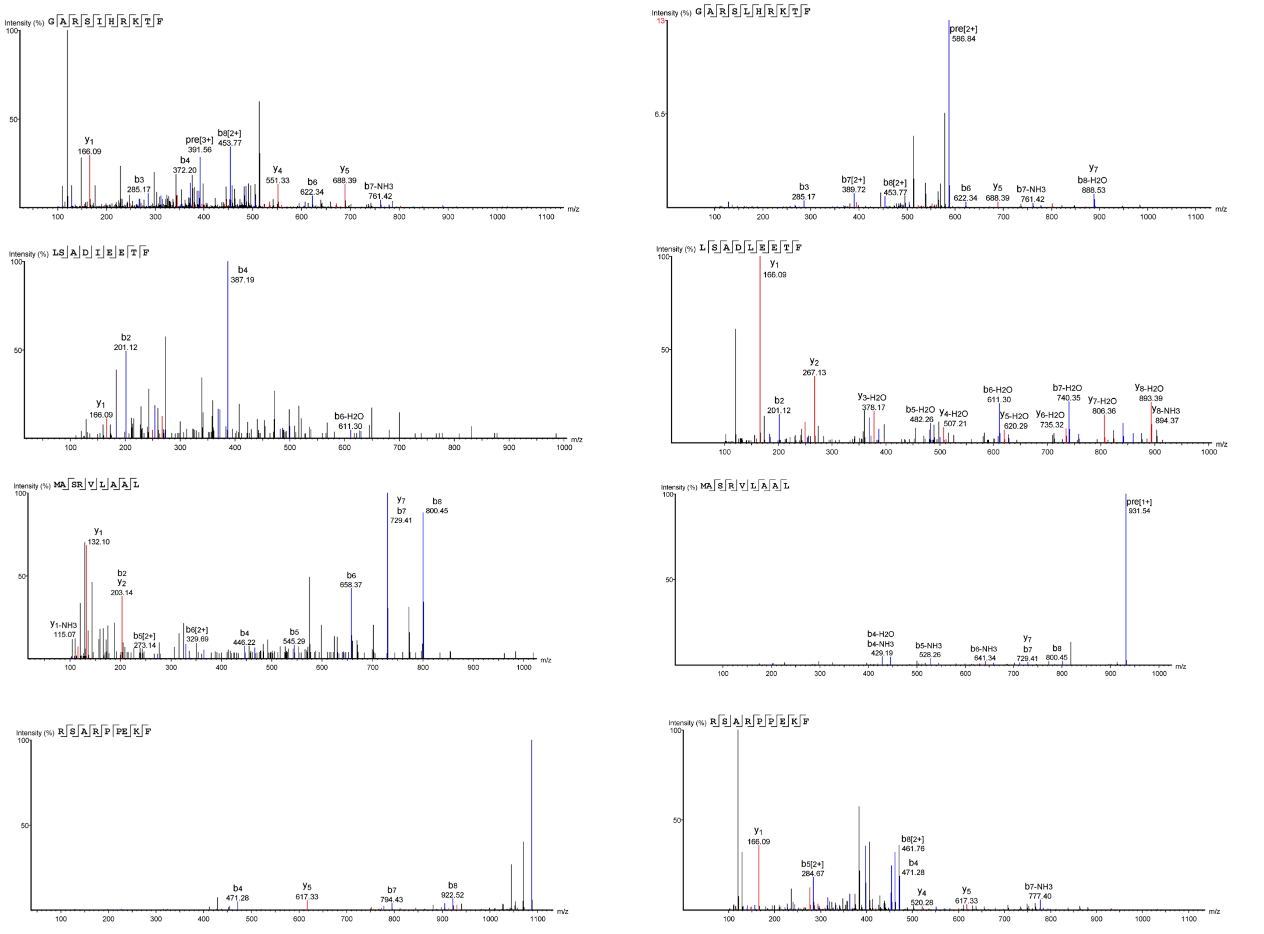

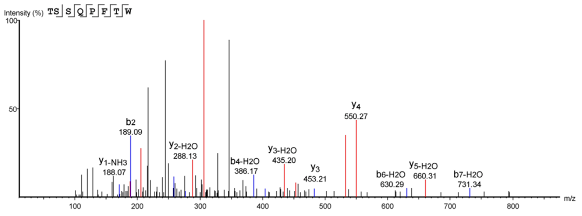
Intensity (%) TSSQPFTW
y1-NH3 188.07
b2 189.09
y2-H2O 288.13
b4-H2O 386.17
y3-H2O 435.20
y3 453.21
y4 550.27
b6-H2O 630.29
y5-H2O 660.31
b7-H2O 731.34
m/z

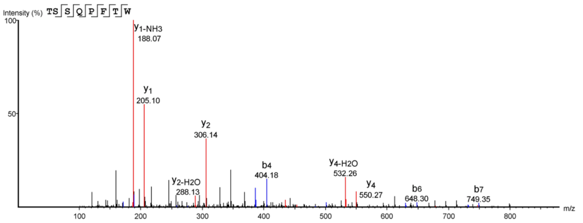
Intensity (%) TSSQPFTW
y1-NH3 188.07
y1 205.10
y2 306.14
y2-H2O 288.13
b4 404.18
y4-H2O 532.26
y4 550.27
b6 648.30
b7 749.35
m/z

C*03:03

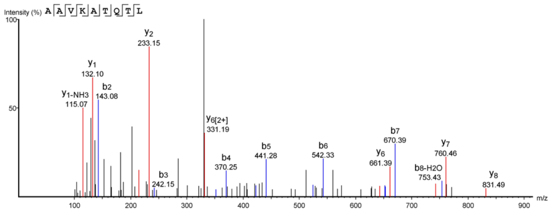
Intensity (%) AAVKATQTL
y2 233.15
y1 132.10
y1-NH3 115.07
b2 143.08
y6[2+] 331.19
b3 242.15
b4 370.25
b5 441.28
b6 542.33
y6 661.39
b7 670.39
y7 760.46
b8-H2O 753.43
y8 831.49
m/z

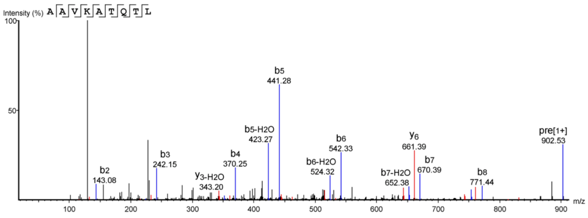
Intensity (%) AAVKATQTL
b5 441.28
b5-H2O 423.27
b6 542.33
y6 661.39
pre[1+] 902.53
b3 242.15
b4 370.25
b2 143.08
y3-H2O 343.20
b6-H2O 524.32
b7 670.39
b7-H2O 652.38
b8 771.44
m/z

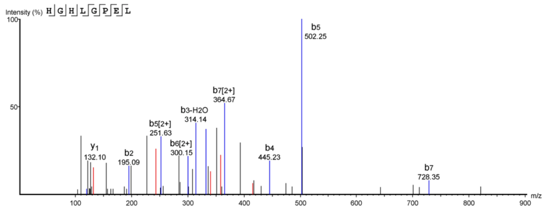
Intensity (%) HGHLGPEL
b5 502.25
b7[2+] 364.67
b3-H2O 314.14
b5[2+] 251.63
b6[2+] 300.15
y1 132.10
b2 195.09
b4 445.23
b7 728.35
m/z

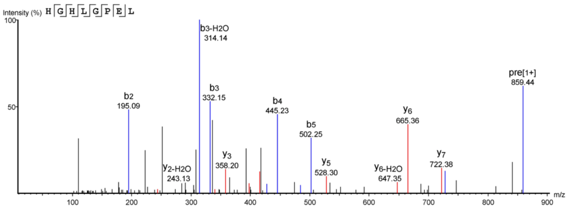
Intensity (%) HGHLGPEL
b3-H2O 314.14
pre[1+] 859.44
b2 195.09
b3 332.15
b4 445.23
y6 665.36
b5 502.25
y3 358.20
y7 722.38
y2-H2O 243.13
y5 528.30
y6-H2O 647.35
m/z

C8166

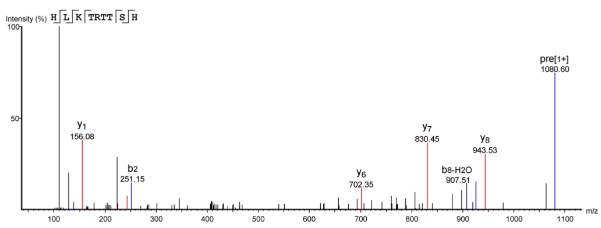
Intensity (%) HLKTRTTSH
pre[1+] 1080.60
y1 156.08
y7 830.45
y8 943.53
b2 251.15
y6 702.35
b8-H2O 907.51
m/z

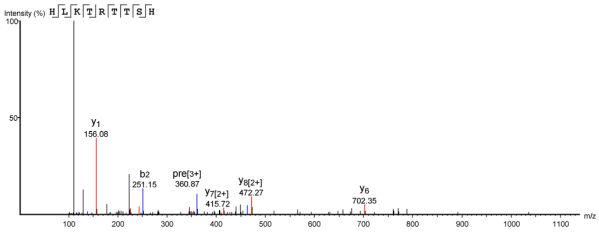
Intensity (%) HLKTRTTSH
y1 156.08
b2 251.15
pre[3+] 360.87
y7[2+] 415.72
y8[2+] 472.27
y6 702.35
m/z

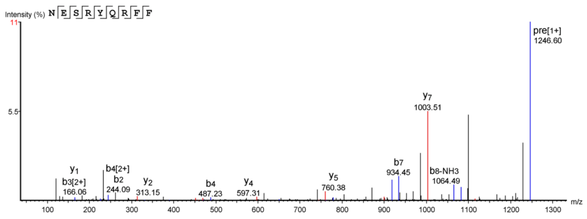
Intensity (%) NESRYQRFF
pre[1+] 1246.60
y7 1003.51
y1
b3[2+] 166.06
b4[2+]
b2 244.09
y2 313.15
b4 487.23
y4 597.31
y5 760.38
b7 934.45
b8-NH3 1064.49
m/z

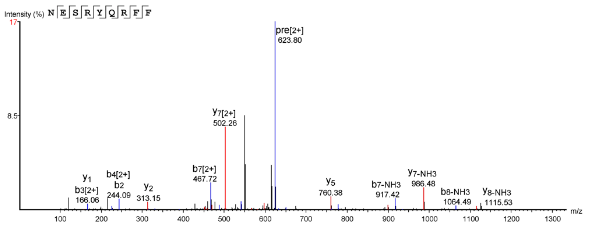
Intensity (%) NESRYQRFF
pre[2+] 623.80
y7[2+] 502.26
b7[2+] 467.72
y1
b3[2+] 166.06
b4[2+]
b2 244.09
y2 313.15
y5 760.38
b7-NH3 917.42
y7-NH3 986.48
b8-NH3 1064.49
y8-NH3 1115.53
m/z

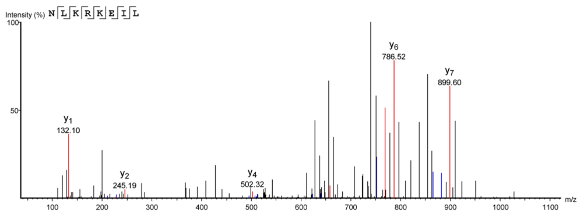
Intensity (%) NLKRKEIL
y6 786.52
y7 899.60
y1 132.10
y2 245.19
y4 502.32
m/z

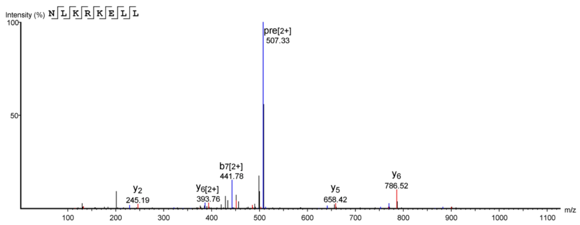
Intensity (%) NLKRKELL
pre[2+] 507.33
b7[2+] 441.78
y6[2+] 393.76
y2 245.19
y5 658.42
y6 786.52
m/z
